# Identification of QTNs, QTN-by-environment interactions, and their candidate genes for grain size traits in main crop and ratoon rice

**DOI:** 10.3389/fpls.2023.1119218

**Published:** 2023-02-02

**Authors:** Qiong Zhao, Xiao-Shi Shi, Tian Wang, Ying Chen, Rui Yang, Jiaming Mi, Ya-Wen Zhang, Yuan-Ming Zhang

**Affiliations:** ^1^ College of Plant Science and Technology, Huazhong Agricultural University, Wuhan, China; ^2^ National Key Laboratory of Crop Genetic Improvement and National Centre of Plant Gene Research (Wuhan), Huazhong Agricultural University, Wuhan, China

**Keywords:** rice, grain size, QTN, QTN-by-environment interaction, ratoon rice, 3VmrMLM

## Abstract

Although grain size is an important quantitative trait affecting rice yield and quality, there are few studies on gene-by-environment interactions (GEIs) in genome-wide association studies, especially, in main crop (MC) and ratoon rice (RR). To address these issues, the phenotypes for grain width (GW), grain length (GL), and thousand grain weight (TGW) of 159 accessions of MC and RR in two environments were used to associate with 2,017,495 SNPs for detecting quantitative trait nucleotides (QTNs) and QTN-by-environment interactions (QEIs) using 3VmrMLM. As a result, 64, 71, 67, 72, 63, and 56 QTNs, and 0, 1, 2, 2, 2, and 1 QEIs were found to be significantly associated with GW in MC (GW-MC), GL-MC, TGW-MC, GW-RR, GL-RR, and TGW-RR, respectively. 3, 4, 7, 2, 2, and 4 genes were found to be truly associated with the above traits, respectively, while 2 genes around the above QEIs were found to be truly associated with GL-RR, and one of the two known genes was differentially expressed under two soil moisture conditions. 10, 7, 1, 8, 4, and 3 candidate genes were found by differential expression and GO annotation analysis to be around the QTNs for the above traits, respectively, in which 6, 3, 1, 2, 0, and 2 candidate genes were found to be significant in haplotype analysis. The gene *Os03g0737000* around one QEI for GL-MC was annotated as salt stress related gene and found to be differentially expressed in two cultivars with different grain sizes. Among all the candidate genes around the QTNs in this study, four were key, in which two were reported to be truly associated with seed development, and two (*Os02g0626100* for GL-MC and *Os02g0538000* for GW-MC) were new. Moreover, 1, 2, and 1 known genes, along with 8 additional candidate genes and 2 candidate GEIs, were found to be around QTNs and QEIs for GW, GL, and TGW, respectively in MC and RR joint analysis, in which 3 additional candidate genes were key and new. Our results provided a solid foundation for genetic improvement and molecular breeding in MC and RR.

## Introduction

Rice (*Oryza sativa* L.) is the principal food for more than half of the population in the world (Rosegrant and Cline, 2003). Effective panicle number per plant, grain number per panicle, and thousand-grain weight (TGW) are three main yield component factors (Xing and Zhang, 2010). Thus, increasing grain weight is an effective way to increase rice yield. TGW is mainly determined by grain size and grouting degree, in which the grain size is determined by grain length (GL), width (GW), and grain thickness (GT). These grain size-related traits are quantitative traits. In addition, grain size not only affects the rice yield but also affects its taste and appearance (Lou et al., 2009; Zhao et al., 2018). Therefore, it is necessary to investigate genetic mechanisms of GL, GW, and TGW.

With the completion of rice genome sequencing, more than 400 quantitative trait nucleotides (QTNs) for rice grain size in different genetic populations have been identified in previous studies (Huang et al., 2013). Among these loci, some of them have been fine-mapped, such as *gw9.1* (Xie et al., 2008), *qGL7* (Bai et al., 2010), *qGL3-2* (Liang et al., 2021), and *qGSN5* (Yuan et al., 2022a). At present, at least 22 QTLs/genes for grain size traits in rice have been cloned and functionally identified (Jiang et al., 2022), for example, *GW2* (Song et al., 2007), *GS2* (Duan et al., 2015), *GS5* (Li et al., 2011), *GS9* (Zhao et al., 2018), *GS3* (Fan et al., 2006), *GL3.1* (Qi et al., 2012), *GL3.3* (Xia et al., 2018), *qGL3* (Zhang et al., 2012), *qTGW2* (Ruan et al., 2020) and *qTGW3* (Hu et al., 2018) were mined by map-based cloning, while *GSE5* (Duan et al., 2017) and *OsSPL13* (Si et al., 2016) were detected by GWAS. Clearly, most were identified by map-based cloning, being a time-consuming work in developing near-isogenic lines. Moreover, it has been shown that the grain size is affected by environmental factors in many previous studies (Arshad et al., 2017; Bahuguna et al., 2017; Wu et al., 2022). However, few QTL-by-environment interactions (QEIs) have been identified in rice grain size. Although many QEIs have been detected in other rice traits in recent years, such as *qGT9* (Rahimsoroush et al., 2021), *qPC6*, *qPC7*, and *qGLU6* (Fiaz et al., 2021), they were identified by linkage analysis rather than genome-wide association studies (GWAS).

Ratoon rice has been considered as an efficient, green, and cost-saving rice cultivation mode, which has been popularized in many countries (Firouzi et al., 2018; Ziska et al., 2018; Wang et al., 2020). Compared with main crop, lower temperature after heading stage affects grain filling to reduce yield and improve quality of ratoon rice (Huang et al., 2020). However, QEIs for grain size between main crop and ratoon rice were rarely reported in previous studies, although main crop is used to identify QTNs and their candidate genes for grain size traits. More importantly, at present, most GWAS report only stable QTNs rather than QEIs, owing to the lack of feasible methodology of QEI detection in multiple environments (Kang et al., 2010; Zhang et al., 2010; Zhou and Stephens, 2012; Jiang et al., 2019b). To address this issue, Li et al. (2022a) and Li et al. (2022b) established a new compressed variance component mixed model method, namely 3VmrMLM, to identify QTNs, QEIs, and QTN-by-QTN interactions under controlling all the possible polygenic backgrounds.

To address the above issues, single environment analysis and two-environment joint analysis *via* 3VmrMLM (Li et al., 2022b) were used to identify QTNs and QEIs for GW, GL, and TGW in main crop (MC) and ratoon rice (RR) of 159 rice accessions with 2,017,495 SNPs. Previously reported genes around QTNs and QEIs for the three traits were mined and their candidate genes were predicted by comparative genomics and confirmed by gene haplotype analysis. In this study, we identified 202 QTNs and 3 QEIs in MC and 191 QTNs and 5 QEIs in RR, 18 previously reported genes around QTNs and two previously reported genes around QEIs were found to be truly associated with grain size in previous studies, and one of two genes around QEIs had the evidence of environmental interaction. Among 25 candidate genes identified by GO annotation and differential expression analysis, 12 were further confirmed by gene haplotype analysis, especially, four candidate genes and one candidate GEI for grain size are more important. In addition, the MC and RR datasets were jointly analyzed as well using 3VmrMLM, as a result, one, two, and one known genes were found to be around QTNs for GW, GL and TGW, respectively, 8 additional candidate genes and 2 candidate GEIs were also mined, in which 3 additional candidate genes are new and key in rice grain size related traits.

## Material and methods

### Plant materials and phenotyping of grain size related traits

All the 159 *indica* rice accessions were planted, with a randomized complete block design, in Wuhan in 2021. This experiment was replicated two times in different fields, namely environments 1 and 2. Each material was planted in one plot with 10 seedlings, row spacing was 16.7 cm × 20 cm, and one line empty between cells. At yellow ripening stage, GW (mm), GL (mm), and TGW (g) for each accession in MC and RR were measured for three times, and their averages were regarded as their trait phenotypes. GL in MC is abbreviated as GL-MC, and it is true for other traits.

### Statistical analysis for the phenotypic data

The minimum, maximum, mean, standard deviation (SD), kurtosis, skewness (S_k_), and coefficient of variation (CV), along with broad-sense heritability $\left(H_{B}^{2}\right)$ , for all the above traits were calculated by R software lme4 v1.1.28. The $H_{B}^{2}$ for each trait was calculated by $H_{B}^{2}=\frac{\sigma_{g}^{2}}{\sigma_{g}^{2}+\sigma_{ge}^{2}/l+\sigma_{e}^{2}/rl}\times 100\%$ , where $\sigma_{g}^{2}$ is genetic variance, $\sigma_{e}^{2}$ is residual variance, $\sigma_{ge}^{2}$ is the variance of genotype-by-environment interaction, *l* is the number of environments, and *r* is the number of replicates. The analysis of variance (ANOVA) for phenotypic data was conducted using the R function *aov.* Normal distribution test for phenotypic data was conducted using the R function *shapiro.test*.

### Genotyping data

The genotypic data of the 159 rice accessions used in this study consisted of two parts. The genotypic datasets of 134 accessions were derived from RiceVarMap database (http://ricevarmap.ncpgr.cn/), and the DNAs of leaves were extracted to conduct 1K Genobaits to verify their authenticity. The genotypic datasets of twenty-five modern breeding cultivars were obtained by double-terminal sequencing with coverage of approximate 10× based on illumina’s Hiseq 4000 technology sequencing platform at Novogene Technology Company. Then, extract the common SNPs from the genotype dataset of 134 public database accessions and 25 modern breeding cultivars to obtain new genotypic datasets with 2,019,008 SNPs. The software plink v1.90 was used to filter all the 2,019,008 SNPs based on minimum allele frequencies (MAFs) < 0.05 and all variants with missing call rates > 10%, where sliding window distance, step length, and R^2^ were set as 1000 kb, 1, and 0.3, respectively. As a result, a total of 2,017,495 SNPs were used in subsequent GWAS.

### Linkage disequilibrium decay and population structure

All the 2,017,495 SNPs were used to conduct linkage disequilibrium (LD) analysis using popLDdecay (https://github.com/BGIShenzhen/PopLDdecay). The LD decay was determined by plotting the r^2^ values against the genetic distance of a pair of loci (kb) for each chromosome. G-matrix and cluster analysis for all the 159 accessions were performed using the 2,017,495 SNPs by R package sommer v4.2.0 and amap v0.8.19, respectively. Principal component analysis (PCA) was analyzed using R function *prcomp*, and the first two principal components were plotted using the R package ggplot2 v3.3.6. ADMIXTURE v1.3.0 (http://dalexander.github.io/admixture) was used to determine population structure (Alexander et al., 2009), where the number of subgroups (K) was set from 1 to 10, and the K value corresponding to the minimum CV error is the most likely subgroup number.

### Multi-locus genome-wide association studies for grain size related traits

A total of 2,017,495 SNPs of 159 rice accessions were used to associate with GW, GL, and TGW in two environments in MC and RR using the 3VmrMLM method and its IIIVmrMLM software (https://github.com/YuanmingZhang65/IIIVmrMLM; Li et al., 2022a; Li et al., 2022b). All parameters were set as default values. Population structure adopts the first three principal components. The K matrix was calculated using the IIIVmrMLM software. The probability threshold was set at 0.05/*m* = 2.48e-08 for significant QTNs and QEIs, where *m* was the number of markers. To reduce the loss of important candidate genes, some insignificant QTNs and QEIs with LOD score ≥ 3.0 were regarded as suggested QTNs and QEIs (Li et al., 2022a; Li et al., 2022b).

### Identification of candidate genes for grain size related traits in rice

Candidate genes for grain size traits were mined based on the below steps. First, all the genes were found in the 200 kb regions of upstream and downstream around each significant QTN without previously reported gene, because the LD decay distance was 150 kb using popLDdecay. Then, RNA-seq datasets of Zhenshan 97 and Minghui 63 at endosperm 7, 14, and 21 days after pollination (https://www.ncbi.nlm.nih.gov/geo/query/acc.cgi?acc=GSE19024) were used to conduct differential expression analysis using NCBI (https://www.ncbi.nlm.nih.gov) GEO2R online tool, and the thresholds of significant difference were set as p-value < 0.05 and | Log2FC | > 1. Finally, all the differentially expressed genes (DEGs) were further analyzed by GO annotation using AgBase (https://agbase.arizona.edu), and the significant E-value was set as 10e-50. If biological process is related to the reported molecular mechanisms of grain size, the DEGs in the biological process were regarded as candidate genes.

### Haplotype analysis of candidate genes

The software plink v1.90 was used to extract all the significant SNP information (P < 0.05) after single marker genome scanning within one candidate gene and its upstream 2 kb, R v4.1.3 was used to calculate its haplotypes of the candidate gene, and the 159 rice accessions were grouped based on these haplotypes. Thus, ANOVA was performed using R function *aov* to test the significance of the QTN-associated trait across these haplotypes at a 5% probability level.

## Result

### Phenotypic variation

**Figure 1:**
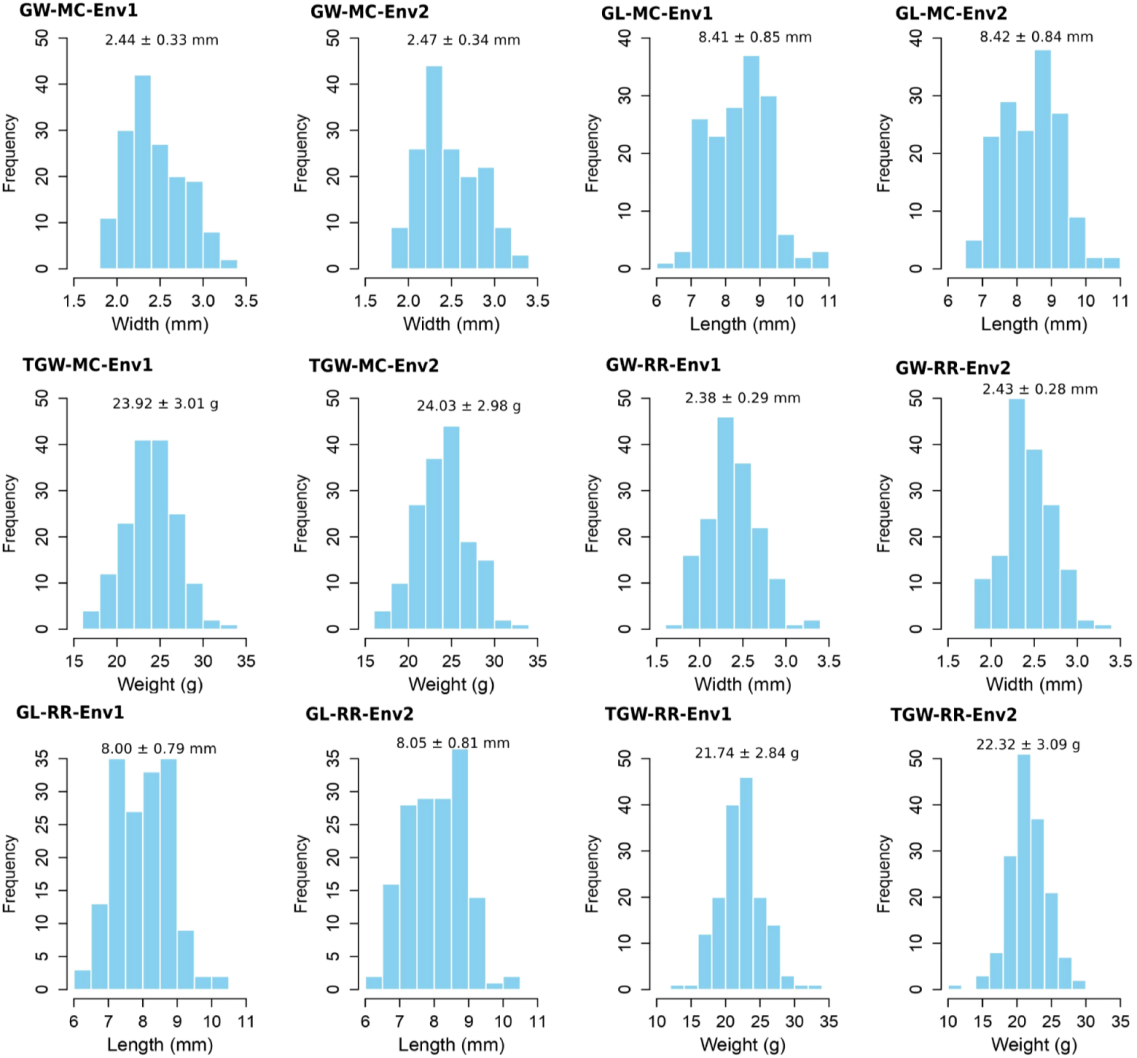
Phenotypic distributions for grain length (GL), grain width (GW), and thousand grain weight (TGW) of 159 accessions of main crop and ratoon rice in two environments.

The averages plus standard deviations of GW-MC, GL-MC, TGW-MC, GW-RR, GL-RR, and TGW-RR in 159 rice accessions in two environments were 2.44 ± 0.33 ~ 2.47 ± 0.34 (mm), 8.41 ± 0.85 ~ 8.42 ± 0.84 (mm), 23.92 ± 3.01 ~ 24.03 ± 2.98 (g), 2.38 ± 0.29 ~ 2.43 ± 0.28 (mm), 8.00 ± 0.79 ~ 8.05 ± 0.81 (mm), 21.74 ± 2.84 ~ 22.32 ± 3.09 (g), and their coefficients of variation (CV) were 13.57 ~ 13.70, 9.91 ~ 10.12, 12.40 ~ 12.58, 11.50 ~ 12.17, 9.91 ~ 10.08, and 13.04 ~ 13.83 (%), respectively, having large phenotypic variations (**Supplementary Table S1**). The analysis of variance was conducted and the results were listed in **Supplementary Table S2**. As a result, genotypes, environments, and their interactions for all the three traits in MC and RR were significant at the 0.05 probability level (**Supplementary Table S2**), and the $H_{B}^{2}$ of GW, GL, and TGW ranged from 96.39% to 99.07% in MC and from 90.21% to 98.37% in RR, indicating large genetic variations (**Supplementary Table S1**). In addition, main crop had higher trait averages than ratoon rice, especially for GL and TGW (**Figure 1**). The phenotypes of TGW-MC and GW-RR in two environments, GL-RR in environment 2, and TGW-RR in environment 1 were found to obey normal distribution, while GW-MC and GL-MC in two environments, and GL-RR and TGW-RR in environment 1 were found to approximately obey normal distribution (**Figure 1**; **Supplementary Table S1**).

### Population structure and linkage disequilibrium analysis

**Figure 2:**
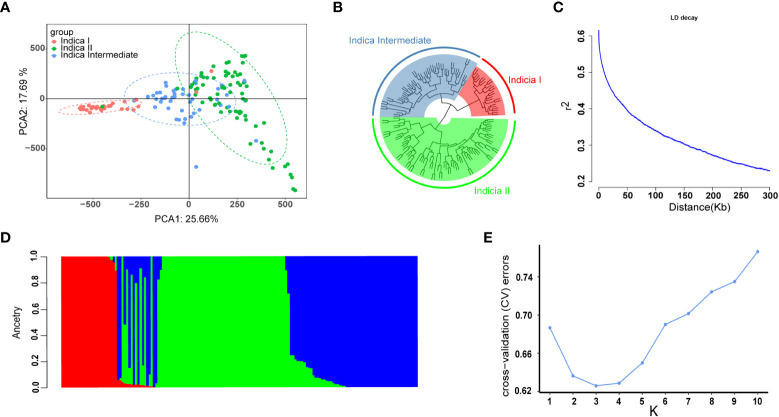
Population structure and LD decay of 159 rice accessions. **(A)** Principal component analysis (PCA) of the association panel. **(B)** Cluster analysis results of 159 rice accessions with 2,017,495 SNPs. **(C)** The entire genome LD decay of the population. **(D)** Population structure estimates (K = 3), the areas of the three colors illustrate the proportion of each subgroup. **(E)** cross-validation (CV) error line graph of subgroups (K = 3).

To determine the LD decay distance, LD decay analysis was performed using all the 2,017,495 SNP markers. The r^2^ gradually decreases with the increase of distance. When it drops to half of the maximum value, the corresponding distance is regarded as the average distance of LD decay. In this study the LD decay distance was 150 kb, when r^2^ dropped to half of its maximum value (r^2^ = 0.3) (**Figure 2C**).

The number of sub-populations was determined by principal component analysis (PCA), population structure analysis, and cluster analysis. The results were showed in **Figure 2**. In PCA, the first two principal components separated all the 159 accessions into three subgroups: indica I, indica II, and indicia Intermediate (**Figure 2A**). In population structure analysis *via* the ADMIXTURE software, cross-validation (CV) error is the lowest when the number of subgroups is three (**Figures 2D, E**), which is consistent with that in cluster analysis (**Figure 2B**). Thus, the first three principal components were used in genome-wide association studies.

### Identification of QTNs and QEIs in main crop and ratoon rice

#### Identification of QTNs and QEIs when two environments in MC or RR were separately and jointly analyzed *via* 3VmrMLM

The 3VmrMLM method, implemented by its IIIVmrMLM software, was used to identify QTNs and QEIs for the three traits in this study. As a result, we identified 64, 71, and 67 QTNs for GW, GL and TGW in main crop, respectively, and 72, 63, and 56 QTNs for GW, GL, and TGW in ratoon rice, respectively (**Supplementary Tables S3–S20** **)**. Among these QTNs for the above three traits in MC, there were 18, 17, and 13 significant QTNs and 2, 1, and 3 suggested QTNs in environment 1, there were 10, 18, and 17 significant QTNs and 2, 2, and 1 suggested QTNs in environment 2 (**Supplementary Tables S3–S5, S9–S11**), and there were 27, 32, and 27 significant QTNs and 5, 1, and 3 suggested QTNs detected in multi-environment joint analysis (**Supplementary Tables S15–S17**). In ratoon rice, there were 16, 13, and 16 significant QTNs and 4, 3, and 2 suggested QTNs in environment 1, there were 20, 13, and 12 significant QTNs and 0, 3, and 4 suggested QTNs in environment 2 **(** **Supplementary Tables S6–S8, S12–S14** **)**, and there were 29, 29, and 20 significant QTNs and 3, 2, and 2 suggested QTNs in multi-environment joint analysis (**Supplementary Tables S18–S20**). More importantly, one GL and two TGW QEIs were detected in main crop, and two GW, two GL, and one TGW QEIs were detected in ratoon rice (**Supplementary Tables S15–S20**).

#### Identification of QTNs and QEIs when the MC and RR datasets were jointly analyzed in each environment *via* 3VmrMLM

The MC and RR datasets in each environment were jointly analyzed using the IIIVmrMLM software. As a result, 34, 35, and 42 QTNs and 7, 1, and 14 QEIs were identified for GW, GL, and TGW, respectively (**Supplementary Tables S29–S34**). Among these QTNs and QEIs, there were 32, 30, and 37 significant QTNs, 2, 5, and 5 suggested QTNs, 4, 0, and 10 significant QEIs, and 3, 1, and 4 suggested QEIs for GW, GL, and TGW, respectively.

### Known genes around QTNs and QEIs

Known genes were searched within 200 kb upstream and downstream regions of QTNs and QEIs. Among the QTNs and QEIs, 3, 4, and 7 known genes were found in main crop to be truly associated with the above three traits, respectively, and 2, 4, and 4 known genes were found in ratoon rice to be truly associated with the above three traits, respectively. Among these known genes, 4 were simultaneously found in main crop and ratoon rice, and 3 were found across multiple traits. 3, 5, and 9 known genes were found to be around significant QTNs and QEIs for the above three traits, respectively, and 0, 1, and 2 known genes were found to be around suggested QTNs for the above three traits, respectively (**Tables 1**, **2**).

**Table 1:** Known genes around QTNs for grain length (GL), grain width (GW), and thousand grain weight (TGW) in main crop (MC) and ratoon rice (RR).

Trait	MC/RR	No.	Chr	Posi (bp)	LOD scores of QTN detection in two environments			r^2^ (%)	Significance	Comparative genomics analysis		Reference
					I	II	I + II			Known genes	Distance (kb)	
GW	Both	1	3	13768754~13863861	14.58		34.25	0.92~1.57	Significant	*VLN2*	8.379~75.176	Wu et al., 2015
	Both	2	5	5357438~5361276	28.87~32.99	21.43~44.21	55.83~93.69	3.75~17.29	Significant	*GW5*	3.846~7.684	Liu et al., 2017
	MC	3	7	24771358		18.09		1.96	Significant	*GW7*	102.037	Wang et al., 2015a
GL	Both	1	3	16708508~16845802	32.72~40.25	47.94	22.1~36.57	2.02~13.09	Significant	*GS3*	11.033~110.693	Mao et al., 2010
	MC	2	3	35504491		5.28		0.68	Suggested	*qTGW3*	112.509	Ying et al., 2018
	MC	3	5	5357676~5456085		10.46	33.37	0.73~1.41	Significant	*GW5*	7.446~89.384	Liu et al., 2017
	Both	4	7	24533051~24800887	10.73		12.68	0.15~0.88	Significant	*GW7*	131.277~136.719	Wang et al., 2015a
TGW	MC	1	1	800544		5.97		0.88	Significant	*SPL33*	129.340	Wang et al., 2017
	MC	2	2	8196020		13.19		2.24	Significant	*GW2*	74.369	Song et al., 2007
	RR	3	2	26049877	17.19			2.10	Significant	*OsVPE3*	148.959	Lu et al., 2016
	RR	4	2	28749717	15.39			1.74	Significant	*GS2*	113.557	Hu et al., 2015
	MC	5	3	35437797			11.11	0.43	Significant	*qTGW3*	45.815	Ying et al., 2018
	RR	6	4	4570606	24.24			4.63	Significant	*ETR2*	167.769	Wuriyanghan et al., 2009
	MC	7	5	5356835			31.77	3.19	Significant	*GW5*	8.287	Liu et al., 2017
	MC	8	6	1540336	4.83			1.91	Suggested	*SSG6*	89.442	Matsushima et al., 2016
	MC	9	7	7640833		16.51		2.47	Significant	*SSH1*	90.919	Jiang et al., 2019a
	RR	10	8	6110721	6.11			2.25	Suggested	*UAP1*	126.728	Wang et al., 2015b
	MC	11	8	25154283	13.02			3.19	Significant	*OsSPL14*	120.258	Jiao et al., 2010

I and II: QTN detection in environments I and II, respectively; I + II: joint analysis of datasets in environments I and II. The same is true for **Table 3**.

**Table 2:** Two known genes around QEIs for rice grain size traits in ratoon rice (RR) and the evidence of gene-by-environment interactions.

No.	Trait	QEI					Known gene	Evidence for environmental interaction genes			Reference
		Chr	Posi (bp)	LOD	r^2^(%)	Significance		Environment	Indicator	Difference of indicator under various environments	
1	GL-RR	4	20228091	15.7721	0.7362	Significant	*OsACOT*	Moderate soil drying	Expression level	The expression of *OsACOT* increased after MD treatment	Zhao et al., 2019; Teng et al., 2022
2	GL-RR	6	26752211	21.2258	1.0259	Significant	*GW6a*				Song et al., 2015

GW, grain width; GL, grain length; TGW, thousand grain weight.

**Figure 3:**
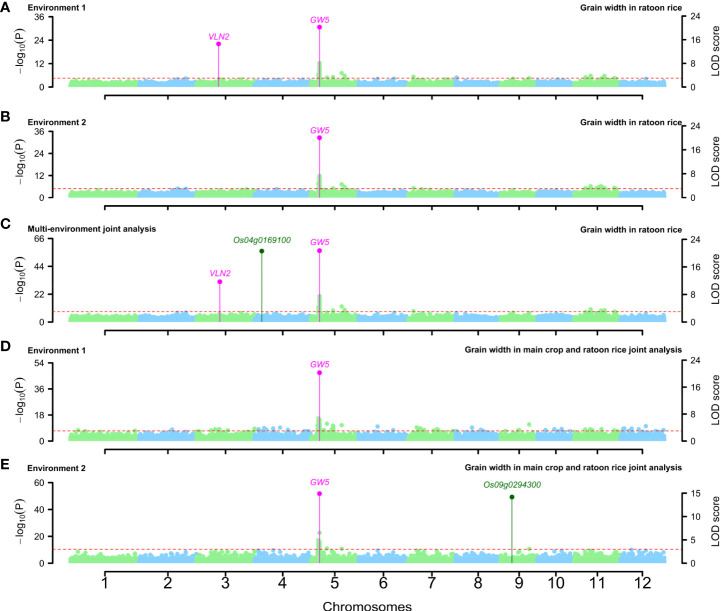
Manhattan plots for grain width in ratoon rice **(A–C)** and grain width in the joint analysis of main crop and ratoon rice **(D, E)**. Known genes around QTNs were marked with magenta color, and candidate genes around QTNs were marked with dark green color.

Around the above QTNs, some known genes were simultaneously mined in single-environment analysis and two-environment joint analysis. *GW5* was identified to be associated with GW-MC and GW-RR in two single-environment analyses and two-environment joint analysis, *VLN2* was identified to be associated with GW-MC in two-environment joint analysis and GW-RR in the first environment analysis (**Figures 3A–C**; **Supplementary Figure 1**), *GS3* was found to be associated with GL-MC and GL-RR in two single-environment analysis and two-environment joint analysis, and *GW5* was found to be associated with GL-MC in the second environment analysis and two-environment joint analysis (**Supplementary Figures 2A–C, 3**). For TGW, all known genes were separately detected in a single-environment analysis or two-environment joint analysis (**Supplementary Figures 4A–C, 5**).

Around the above QEIs, two known genes, *OsACOT* and *GW6a*, for GL-RR were mined (**Table 2**). Among the two known genes, *OsACOT* was found to be interacted with environments. In detail, its expression level under moderate soil drying treatment was higher than that under well-watered control (Teng et al., 2022) (**Table 2**).

In the joint analysis of the MC and RR datasets, 1, 2, and 1 known genes were found to be around significant QTNs and to be truly associated with GW, GL, and TGW, respectively (**Supplementary Tables S35**; **Figures 3D, E**; **Supplementary Figures 2, 4D, E**). Among these known genes, most of them were consistent with the above known genes, such as *GW5, GS3*, and *qTGW3*, but *PGL2* was found only in the MC and RR joint analysis.

### Prediction of candidate genes

Around other QTNs without known genes, all the genes within 200 kb upstream and downstream regions were used to conduct differential expression analysis. All the differential expression genes (DEGs) were used to conduct gene annotation analysis. In gene annotation analysis, the significant biological processes were mainly included the below categories: cytokinin, abscisic acid and other plant hormone metabolism (e.g., *Os02g0197600*, *Os02g0621300*, *Os02g0626100*, and *Os02g0178800*), protein ubiquitination (*Os07g0166800*), sucrose starch metabolism (*Os04g0169100*, *Os12g0112500*, and *Os08g0205900*), protein phosphorylation (*Os02g0126400*, and *Os03g0717700*), and endosperm development (*Os02g0538000*, *Os12g0277500*, and *Os01g0280500*) (**Supplementary Tables S21, S22**), which are highly consistent with the previously reported regulatory pathways in Zuo and Li (2014); Cai et al. (2018), and Li et al. (2019). These genes were regarded as candidate genes. As a result, there were 10, 7 and 1 candidate genes for GW, GL and TGW in main crop, respectively, and 8, 4, and 3 candidate genes for GW, GL and TGW in ratoon rice, respectively (**Supplementary Tables S21, S22**).

For candidate genes for GW, *Os02g0126400* and *Os03g0717700* were predicted to be related to protein phosphorylation, *Os02g0178800* and *Os03g0592500* were predicted to respond to abscisic acid, *Os02g0197600* was found to be related to cytokinin, *Os07g0166800* was found to be related to the process of protein ubiquitination, *Os02g0538000* and *Os12g0277500* were found to be associated with embryonic development at the end of seed dormancy, and *Os08g0205900* and *Os04g0169100* were predicted to be related to sucrose metabolism and starch synthesis metabolism, respectively.

For candidate genes for GL, *Os02g0197600*, *Os02g0621300*, *Os02g0626100, Os04g0514800*, and *Os03g0108600* were predicted to respond to cytokinin, abscisic acid, gibberellin, auxin, and ethylene, respectively, *Os01g0280500* and *Os02g0538000* were found to affect embryonic development, and *Os04g0169100* and *Os12g0112500* were predicted to be related to starch synthesis. In Wuriyanghan et al. (2009), *Os04g0169100* was reported to affect the ethylene sensitivity of seeds to substantially enhance TGW of mutant. Here there is one issue pending, that is, whether the TGW increase is caused by the GL increase.

For candidate genes for TGW, *Os02g0621300* was predicted to be related to response to abscisic acid, *Os03g0411500* was predicted to be related to photosynthesis, *Os03g0607400* was predicted to be related to positive regulation of unidimensional cell growth, and *Os05g0445900* was predicted to participate in DNA demethylation, which is consistent with that in Zhou et al. (2021), in detail, *Os05g0445900* encodes rice DNA glycosylase, and the mutation of *DNG701* can lead to embryo retardation or abortion of part seeds (Zhou et al., 2021).

For the DEG *Os03g0737000* (P=7.77E-03, log2FC=-1.29) around the QEI of chr3-30340995 for GL-MC, *Os03g0737000* was predicted to be related to “response to salt stress” (**Table 3**). In the future, new experiments are necessary to explore these novel gene-trait and GEI-trait associations.

**Table 3:** Key candidate genes and gene-by-environment interactions for grain size related traits in rice.

QTN/GEI	No.	Trait	Locus		LOD scores			r^2^ (%)	Gene differential expression analysis			P-value in haplotype analysis	GO annotation analysis			
			Chr	Posi (bp)	II	I + II	MC+RR		Gene_ID	log2(Fold Change)	P-value		GO_ID	GO_name	E-value	Reference
QTN	1	GW-MC	2	20073320		11.87		0.47	*Os02g0538000*	1.23	6.47E-03	1.50E-06	GO:0009793	embryo development ending in seed dormancy	0	
QTN	2	GL-MC	2	24992114	13.56			0.75	*Os02g0626100*	-1.27	9.33E-03	7.30E-03	GO:0009739	response to gibberellin	0	
QTN	3	GW-RR	4	4591488		49.91		0.97	*Os04g0169100*	-1.20	2.76E-03	4.06E-15	GO:2000904	regulation of starch metabolic process	0	Wuriyanghan et al., 2009
QTN	4	TGW-MC	5	22017452		10.27		1.82	*Os05g0445900*	1.19	1.48E-02	1.32E-03	GO:0080111	DNA demethylation	0	Zhou et al., 2021
QTN	5	TGW	8	17687290			11.62	1.71	*Os08g0379300*	-1.18	2.72E-02	7.20E-03	GO:0005983	starch catabolic process	0	
QTN	6	GW	9	6986114			14.17	0.79	*Os09g0294300*	-1.6	3.46E-03	1.70E-05	GO:0016567	protein ubiquitination	2.71E-288	
QTN	7	GL	12	22906272			12.45	1.52	*Os12g0557800*	1.79	4.95E-03	1.30E-06	GO:0009737	response to abscisic acid	5.65E-216	
GEI	1	GL-MC	3	30340995		6.39		0.20	*Os03g0737000*	-1.29	7.77E-03		GO:0009651	response to salt stress	0	
GEI	2	TGW	6	2928548			13.19	2.91	*Os06g0154200*	-1.22	1.11E-02		GO:1902584	positive regulation of response to water deprivation	0	
GEI	3	TGW	11	22930659			5.21	1.08	*Os11g0600900*	-1.14	2.01E-02		GO:0009642	response to light intensity	0	

GW, grain width; GL, grain length; TGW, thousand grain weight; MC, main crop; RR, ratoon rice.

In the joint analysis of the MC and RR datasets, there were 22 candidate genes around QTNs to be responsible for the above three traits, but only two were consistent with the above 25 candidate genes (**Supplementary Table S36**). The significant biological processes of these candidate genes were mainly included the below categories: plant hormone metabolic pathway (e.g., *Os02g0126400*, *Os05g0563400*, and *Os12g0288000*), protein phosphorylation (*Os03g0838100, Os08g0200500*, and *Os05g0514200*), embryo development (*Os02g0538000* and *Os08g0428100*), and protein ubiquitination (*Os09g0294300* and *Os12g0111500*). In addition, we also mined two additional DEGs for TGW around QEIs, among which *Os06g0154200* was predicted to be related to “positive regulation of response to water deprivation” and *Os11g0600900* was predicted to be related to “response to light intensity” (**Table 3**).

### Haplotype analysis

**Figure 4:**
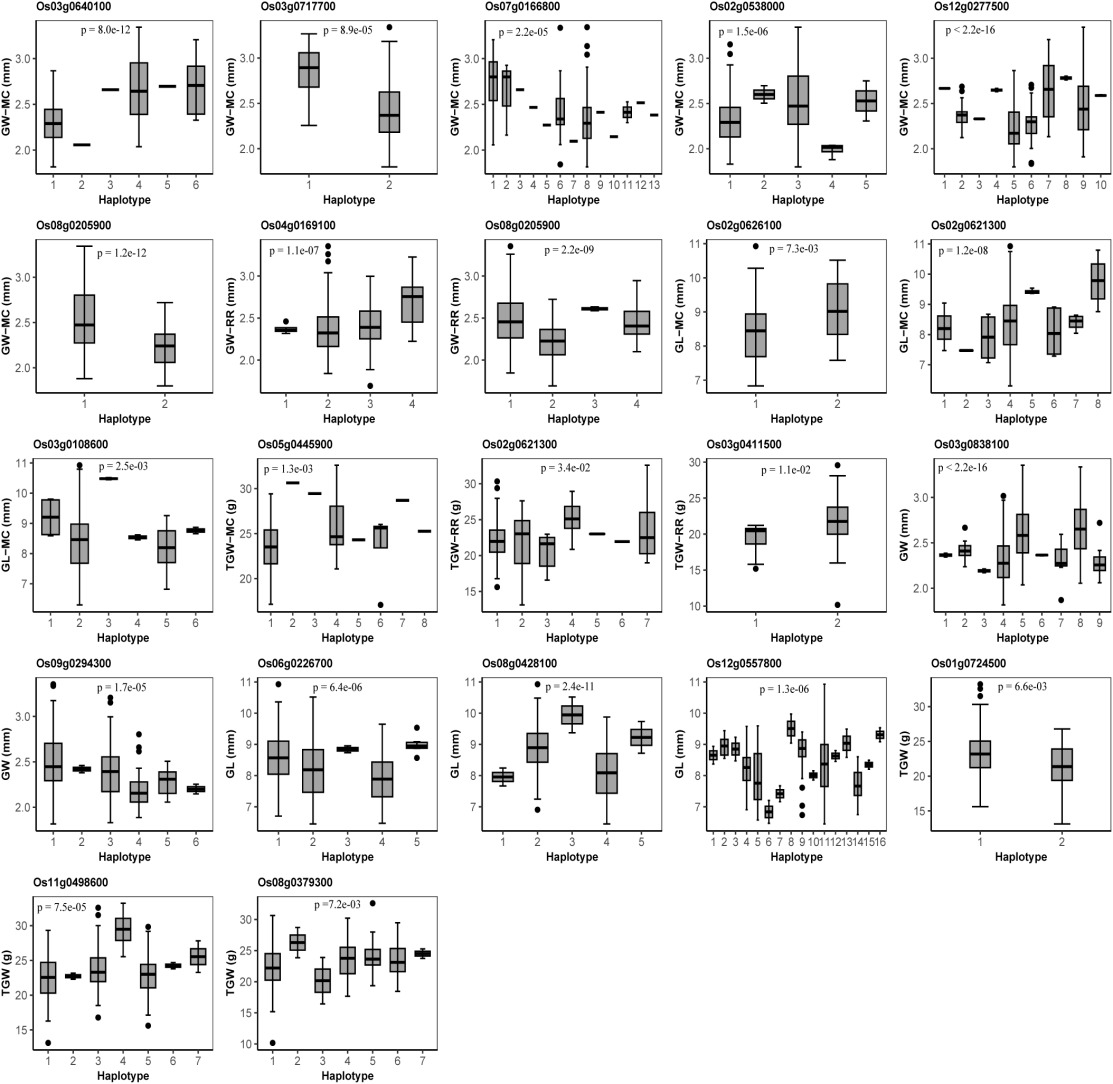
Haplotype analysis for candidate genes for grain width (GW), grain length (GL) and thousand grain weight (TGW) in main crop (MC), ratoon rice (RR), and the joint analyses of MC and RR. The P-values indicate the significance of trait averages across gene haplotypes for GW, GL, and TGW in one-way ANOVA.

To further verify the reliability of candidate genes, we conducted haplotype analysis. As a result, 12 of the above 25 candidate genes had significant differences among the phenotypes of the traits corresponding to the haplotypes of each gene (**Figure 4**). Among the 12 significant candidate genes, 7, 3, and 3 were found to be associated with GW, GL, and TGW, respectively, of which there are 6, 3, and 1 significant candidate genes in main crop and 2, 0, and 2 significant candidate genes in ratoon rice (**Figure 4**). *Os08g0205900* for GW was mined in both MC and RR, and *Os02g0621300* was found in both GL and TGW (**Figure 4**). It should be noted that 8 of 22 candidate genes, which were mined in the MC and RR joint analysis, were significant in haplotype analysis, and the eight genes were different from the 12 significant candidate genes in the above haplotype analysis (**Figure 4**).

## Discussion

To address the studies on gene-by-environmental interactions, especially, across main crop and ratoon rice, in this study we conducted genome-wide association studies for GW, GL, and TGW using 3VmrMLM. As a result, a total of 202 QTNs and 3 QEIs in main crop, and 191 QTNs and 5 QEIs in ratoon rice were identified. Around these QTNs and QEIs, 18 and 2 known genes were found to be truly associated with the grain size related traits, in which 4 were common across main crop and ratoon rice, and 12 candidate genes were mined through differential expression analysis, GO annotation, and haplotype analysis, in which one was common across main crop and ratoon rice. More importantly, four key candidate genes around QTNs were predicted, in which two were new and all identified in main crop. In addition, we identified a new candidate GEI *Os03g0737000*, which was predicted to be related “response to salt stress”. In the joint analysis of the MC and RR datasets, furthermore, 8 additional candidate genes and two additional GEIs were minded, and 3 of 8 additional candidate genes were new and key for grain size related traits in this study.

### Comparison of QTNs, QEIs, known genes, and candidate genes across main crop and ratoon rice

Ratoon rice is a new mode of rice planting, which can effectively save costs and increase benefits (Shen et al., 2021). Although it is generally accepted that RR has higher quality than MC, there are still many controversies in the studies on grain size related traits (Alizadeh and Habibi, 2016; Huang et al., 2020; Yuan et al., 2022b).

In this study, 64, 71, and 64 QTNs and 0, 1 and, 2 QEIs in MC, and 72, 63 and 56 QTNs and 2, 2, and 1 QEIs in RR were identified to be associated with GW, GL and TGW, respectively. Among these QTNs, 4 known genes were commonly detected in main crop and ratoon rice to be truly associated with grain size related traits, including *GW5* and *VLN2* for GW, and *GS3* and *GW7* for GL (**Table 1**). Some known genes were found only in main crop or ratoon rice, such as *qTGW3*, *SPL33*, and *OsSPL14* were detected only in main crop, and *OsVPE3*, *GS2*, *ETR2*, and *UPA1* were found only in the ratoon rice (**Table 1**). Among all the candidate genes, one was commonly found in main crop and ratoon rice, and 12 were detected only in main crop or ratoon rice (**Figure 4**). No common QEIs were detected between main crop and ratoon rice.

Based on the above results, main crop can detect more known genes (14), candidate genes (10) and candidate GEIs (1) than ratoon rice (8, 4, and 0). Although some known and candidate genes can be commonly found in main crop and ratoon rice, there are still some specific candidate genes in main crop or ratoon rice. In the independent and joint analyses of the MC and RR datasets, most candidate genes and candidate GEIs were different across the two analyses. This indicated that more known and candidate genes and GEIs can be identified while the datasets in main crop and ratoon rice are simultaneously or jointly analyzed.

### Key candidate genes for GW, GL, and TGW in rice

The candidate genes were mined by expression and GO annotation analysis, and further validated through haplotype analysis. In this study we identified five new and key candidate genes that were predicted to be closely related to the three traits, among which 3 were mined to be around QTNs in the MC and RR joint analysis (**Table 3**), the evidence was as below.

*Os02g0626100* for GL-MC, and *Os02g0538000* for GW-MC were differentially expressed. In GO annotation analysis, the two genes were annotated as “response to gibberellin”, and “embryo development ending in seed dormancy”, respectively, in which these biological processes are highly consistent with metabolic pathways of important grain size traits in rice (Li et al., 2018; Li et al., 2020; Jiang et al., 2022). In haplotype analysis, significant GL/GW differences were observed across 2 and 5 haplotypes from 1 and 11 significant SNPs within the two genes and their 2 kb upstream. Thus, the two genes may be important candidate genes for GL/GW.

In the same way, *Os08g0379300* for TGW, *Os09g0294300* for GW, and *Os12g0557800* for GL were found to be DEGs around the QTNs in the MC and RR joint analysis. In GO annotation analysis, the three gene were predicted to be related to “starch catabolic process”, “protein ubiquitination”, and “response to abscisic acid”, respectively, which have been confirmed to be important regulatory pathways of rice grain size (Li et al., 2008; Choi et al., 2018; Gao et al., 2021). In haplotype analysis, significant TGW/GW/GL difference was observed across 7, 6, and 16 haplotypes from 6, 6, and 16 significant SNPs within the genes and their 2 kb upstream. Thus, the three genes may be important candidate genes for TGW/GW/GL.

In addition, two candidate genes have been reported to be related to rice seed development. In Wuriyanghan et al. (2009), *Os04g0169100*, identified for GW-RR in this study, significantly increased TGW of mutants by increasing the sensitivity of seeds to ethylene. In Zhou et al. (2021), the mutant of *Os05g0445900*, identified for TGW-MC in this study, participated in DNA methylation process causing the endosperm of some seeds to be stunted or aborted.

### Identification of known and candidate GEIs for grain size traits in rice

Around the QEIs, *OsACOT* for GL-RR has been confirmed to be differentially expressed under two soil moisture treatments (Teng et al., 2022; **Table 2**).

Around QEIs in the independent analysis of MC or RR, *Os03g0737000* for GL-MC was found to be differentially expressed, and its biological process in GO annotation was predicted to be related to salt stress. We speculate that *Os03g0737000* may be affected by environmental factors, such as different salt treatments. Around QEIs detected in the MC and RR joint analysis, *Os06g0154200* and *Os11g0600900* for TGW were found to be differentially expressed, and the biological processes in their GO annotations were predicted to be related to water deprivation and light intensity, respectively. Thus, we speculate that *Os06g0154200* may be affected by the moisture content of the environment and *Os11g0600900* may be affected by the intensity of external light. The molecular functions of above three candidate GEIs need to be verified by subsequent molecular biology experiments.

### Comparison of known genes across two types of interval lengths

To investigate the effect of interval length on mining known genes, two types of interval lengths were compared. One was 200 kb upstream and downstream regions of QTNs and QEIs, which was determined based on LD decay distance, while another was 1000 kb for QTNs and 1500 kb for QEIs. The results are listed in **Table 1** and **Supplementary Table S37**. As a result, 3, 4, 7, 2, 2, and 4 known genes around QTNs and 0, 0, 0, 0, 2, and 0 known genes around QEIs were found to be located on their corresponding 200 kb upstream and downstream regions and to be truly associated with GW-MC, GL-MC, TGW-MC, GW-RR, GL-RR, and TGW-RR, respectively, while 6, 7, 21, 7, 6, and 16 known genes for the above six traits were found to be located on 1000 kb upstream and downstream regions of QTNs, and 0, 0, 1, 2, 2, and 0 known genes for the above six traits were found to be located on 1500 kb upstream and downstream regions of QEIs. This indicates that large intervals can find more known genes. Thus, it is very important to determine a suitable interval length in mining known genes.

### Comparison of QTNs, QEIs, and known genes across various population structures

To investigate the effect of population structure on genome-wide association studies, we compared the results from evolutionary population structure (Liu et al., 2020), Q matrix, and PCA in this study. As a result, 323, 283, and 393 QTNs, 9, 6, 8 QEIs, and 11, 12, and 20 known genes were identified from evolutionary population, Q matrix and PCA, respectively **(** **Supplementary Tables S23–S28** **)**. Clearly, the PCA result is the best, followed by evolutionary population, and the worst is the Q matrix result in this study. Thus, population structure is an important parameter in genome-wide association study.

## Data availability statement

The genotypic datasets of 134 rice accessions can be downloaded from the NCBI under accession numbers PRJNA171289 and PRJEB6180, the phenotype values of grain size traits can be downloaded from figshare (https://doi.org/10.6084/m9.figshare.21957449.v1), RNA-seq data from endosperm tissue of Zhenshan 97 and Minghui 63 are available in the NCBI Gene Expression Omnibus (GEO) database under the accession number GSE19024, and further inquiries can be directed to JMM (mjm@mail.hzau.edu.cn).

## Author contributions

Y-WZ and JMM managed the research. JMM was in charge of research experiments. TW and RY measured trait phenotypes and marker genotypes. QZ, X-SS, and YC analyzed datasets and mined candidate genes. QZ wrote the draft. Y-MZ, Y-WZ, and J-MM revised the manuscript. All authors contributed to the article and approved the submitted version.
