# Supplementary material for: Identification of QTNs, QTN-by-environment interactions, and their candidate genes for grain size traits in main crop and ratoon rice
Source: Front Plant Sci. 2023 Feb 2;14:1119218. doi: 10.3389/fpls.2023.1119218 (PMC9933869; doi:10.3389/fpls.2023.1119218)
Supplement: Supplementary file 1 [file DataSheet_1.docx]

Supplementary Material

Identification of QTNs, QTN-by-environment interactions, and their candidate genes for grain size traits in main crop and ratoon rice

Qiong Zhao#, Xiao-Shi Shi#, Tian Wang, Ying Chen, Rui Yang, Yuan-Ming Zhang

*** Correspondence:** Jiaming Mi: [mjm@mail.hzau.edu.cn](mailto:mjm@mail.hzau.edu.cn), Ya-Wen Zhang: yawen@mail.hzau.edu.cn

# Supplementary Figures and Tables

## 1.1 Supplementary Figures


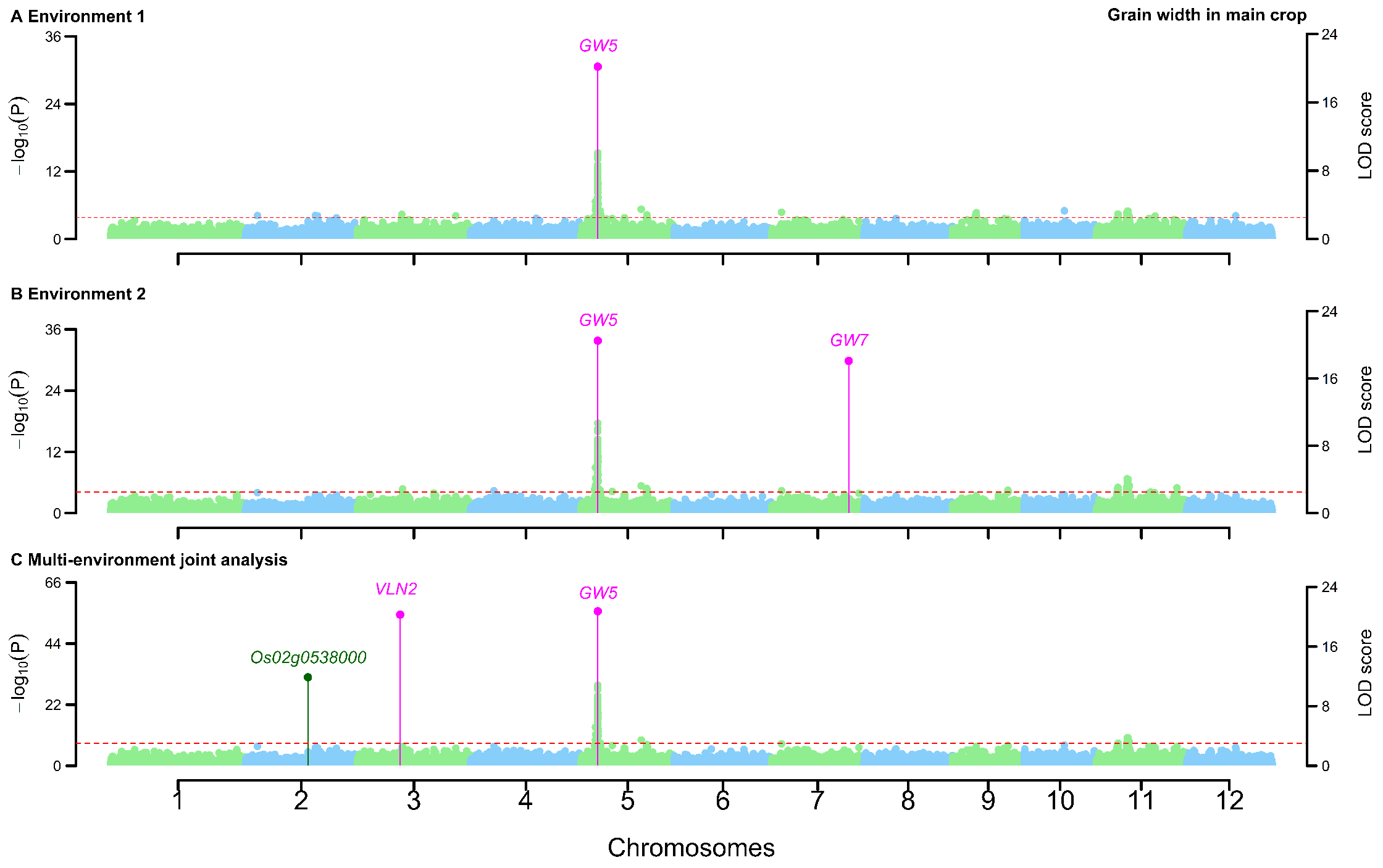


**Supplementary Figure 1.** **Manhattan plots for the GWAS for grain width in main crop.** Known genes around QTNs were marked with magenta color, and candidate gene around QTN was marked with dark green color.

**
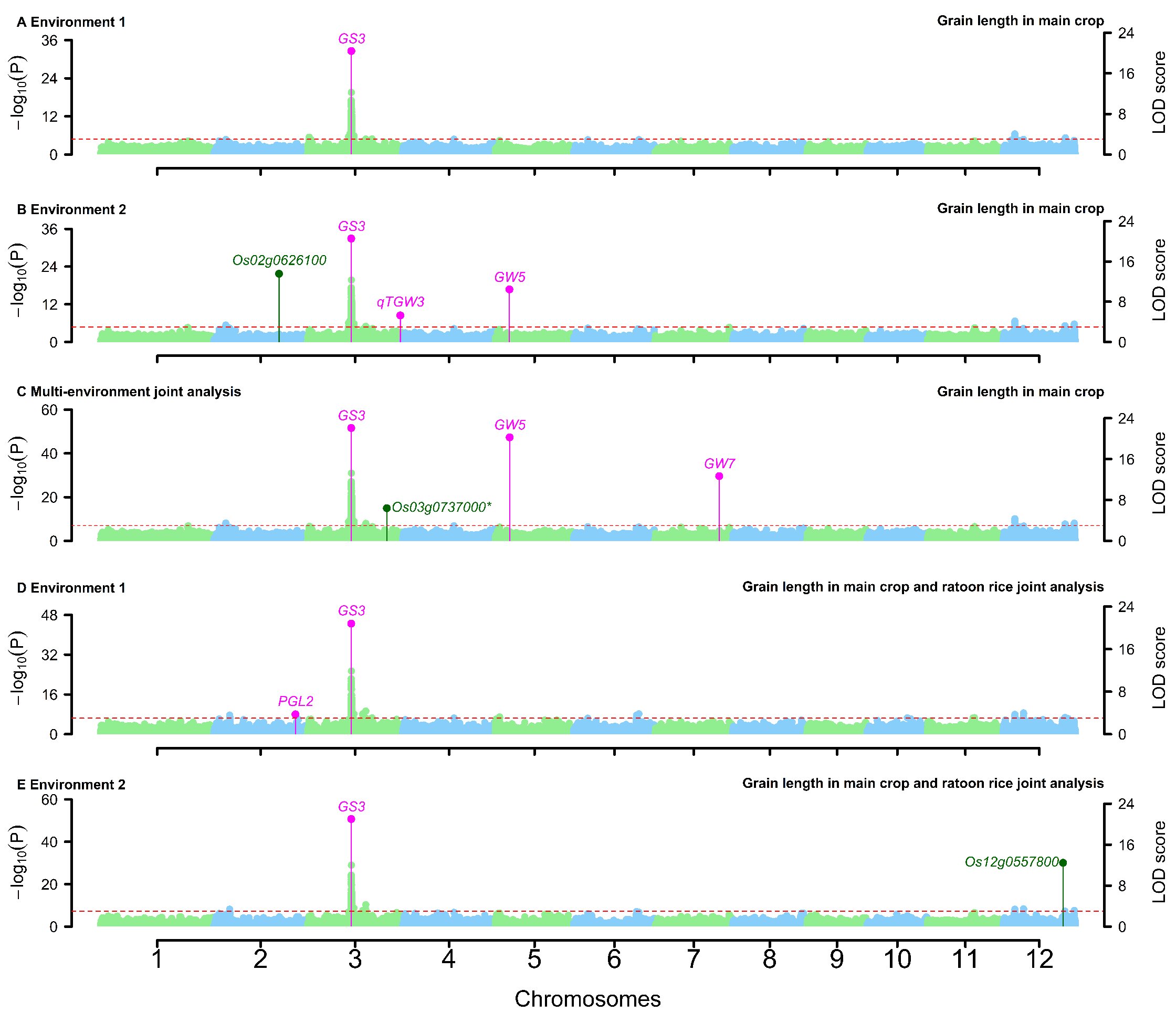
**

**Supplementary Figure 2. Manhattan plots for grain length in main crop (A-C) and grain width in the joint analysis of main crop and ratoon rice (D-E).** Known genes around QTNs were marked with magenta color, candidate genes around QTN was marked with dark green, and candidate gene around QEI was marked with dark green and stat (*).


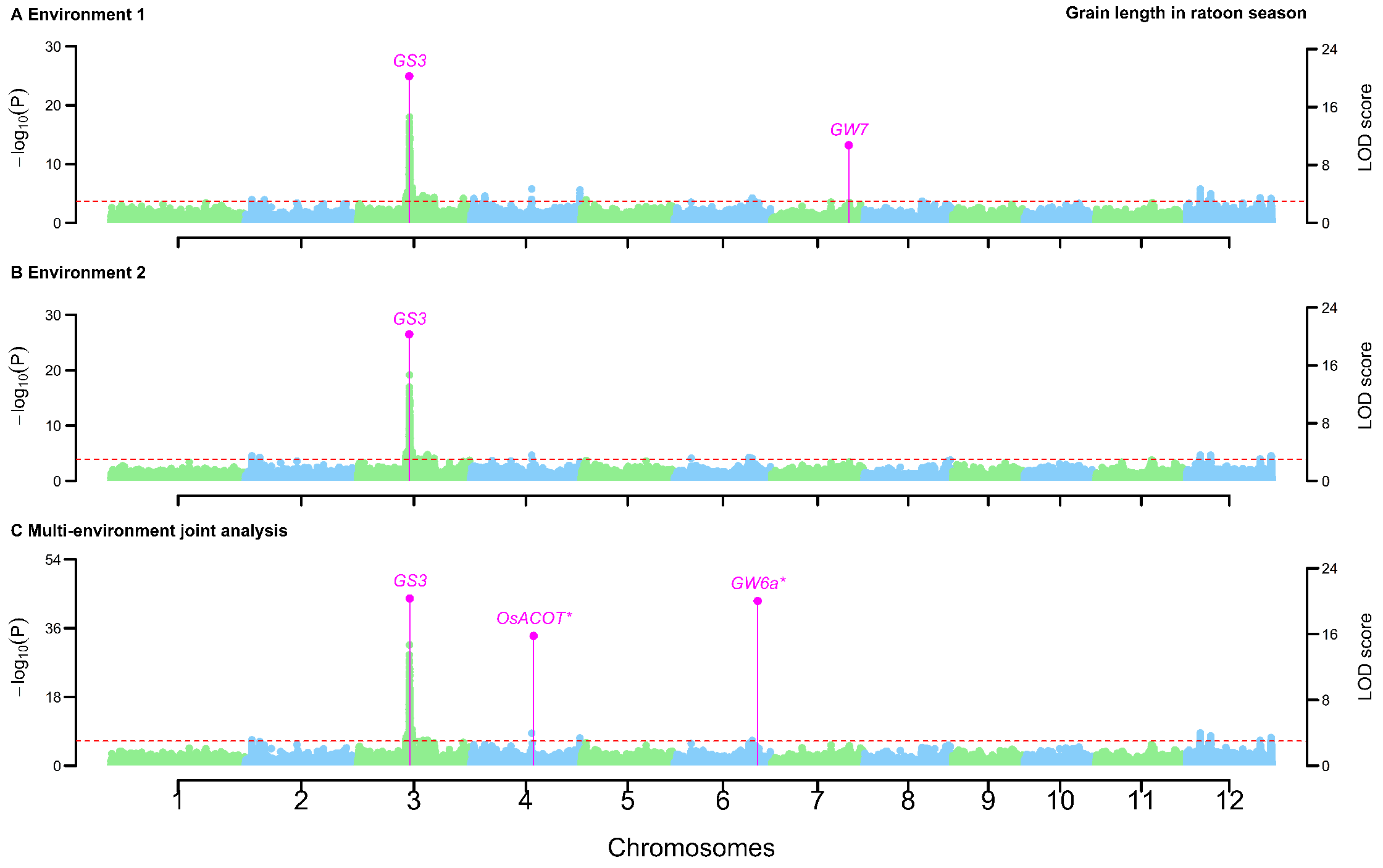


**Supplementary Figure 3.** **Manhattan plots for grain length in ratoon rice.** Known genes around QTNs were marked with magenta color, known genes around QEIs were marked with magenta color and star (*).


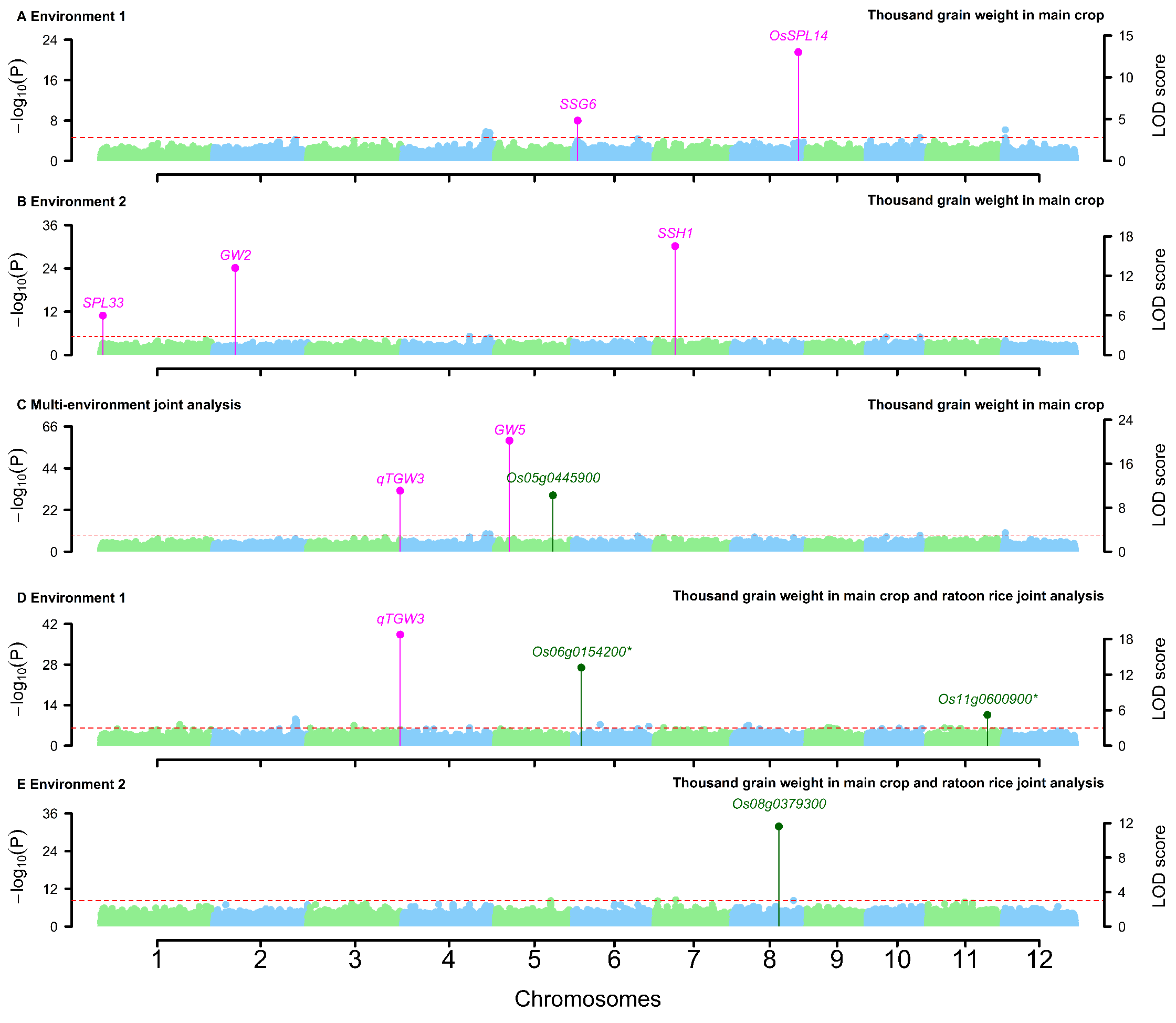


**Supplementary Figure 4.** **Manhattan plots for thousand grain weight in main crop (A-C) and grain width in the joint analysis of main crop and ratoon rice (D-E).** Known genes around QTNs were marked with magenta color, candidate gene around QTN was marked with dark green color, and candidate gene around QEI was marked with dark green color and star (*).


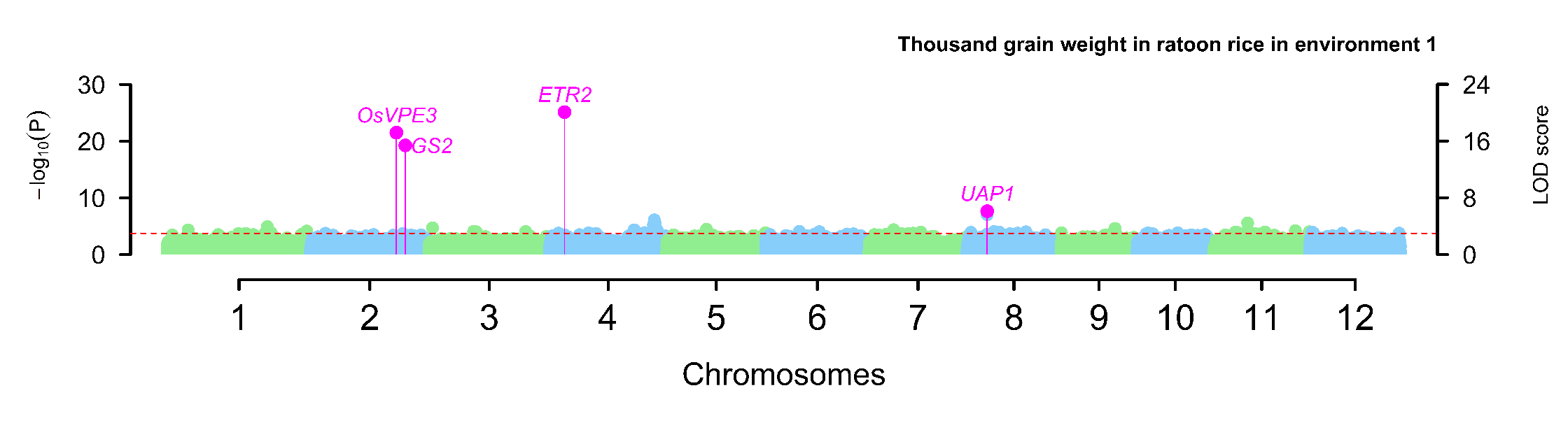


**Supplementary Figure 5.** **Manhattan plots for the GWAS for thousand grain weight in ratoon rice.** Known genes around QTNs were marked with magenta color.

## 1.2 Supplementary Tables

**Table S1 Descriptive statistics and normal distribution test of grain size in 156 rice accessions in 2 environments**

| **Trait** | **Location** | **Ave** | **Max** | **Min** | **Std. dev** | **Kurtosis** | **Skewness** | **CV (%)** | ***h2B*(%)** | **W** | **P-value** |
| --- | --- | --- | --- | --- | --- | --- | --- | --- | --- | --- | --- |
| GW-MC | Env1 | 2.44 | 3.34 | 1.80 | 0.33 | -0.47 | 0.38 | 13.57 | 98.94 | 0.9785 | 0.01394 |
|  | Env2 | 2.47 | 3.34 | 1.82 | 0.34 | -0.52 | 0.41 | 13.70 |  | 0.97318 | 0.003452 |
| GL-MC | Env1 | 8.41 | 10.80 | 6.30 | 0.85 | -0.15 | 0.18 | 10.12 | 99.07 | 0.98091 | 0.02691 |
|  | Env2 | 8.42 | 10.93 | 6.83 | 0.84 | -0.35 | 0.19 | 9.91 |  | 0.98176 | 0.03398 |
| TGW-MC | Env1 | 23.92 | 33.23 | 16.27 | 3.01 | 0.17 | 0.04 | 12.58 | 96.39 | 0.99457 | 0.8235 |
|  | Env2 | 24.03 | 32.60 | 17.08 | 2.98 | -0.05 | 0.03 | 12.40 |  | 0.99345 | 0.6923 |
| GW-RR | Env1 | 2.38 | 3.26 | 1.69 | 0.29 | 0.05 | 0.31 | 12.17 | 97.66 | 0.98778 | 0.1803 |
|  | Env2 | 2.43 | 3.36 | 1.85 | 0.28 | 0.05 | 0.32 | 11.50 |  | 0.9879 | 0.1865 |
| GL-RR | Env1 | 8.00 | 10.26 | 6.35 | 0.79 | -0.36 | 0.20 | 9.91 | 98.37 | 0.98265 | 0.0435 |
|  | Env2 | 8.05 | 10.36 | 6.45 | 0.81 | -0.48 | 0.12 | 10.08 |  | 0.99109 | 0.4225 |
| TGW-RR | Env1 | 22.32 | 32.58 | 13.13 | 3.09 | 0.62 | 0.23 | 13.83 | 90.21 | 0.98576 | 0.1034 |
|  | Env2 | 21.74 | 29.57 | 10.17 | 2.84 | 1.26 | -0.20 | 13.04 |  | 0.9785 | 0.01394 |

GW: grain width; GL: grain length; TGW: thousand grain weight; Env1: environment1; Env2: environment 2

**Table S2 Analysis of variance for grain size traits of 159 rice accessions in two environments**

| **Traits** | **Source of variation** | **df** | **Main crop** | | |  | **Ratoon rice** | | |
| --- | --- | --- | --- | --- | --- | --- | --- | --- | --- |
|  |  |  | **MS** | **F** | **Pr(>F)** |  | **MS** | **F** | **Pr(>F)** |
| GW | Genotypes (G) | 158 | 0.668 | 488.96 | <2E-16 |  | 0.437 | 146.69 | <2E-16 |
|  | Environment (E) | 1 | 0.202 | 146.92 | <2E-16 |  | 0.491 | 164.94 | <2E-16 |
|  | G×E interactions | 158 | 0.007 | 5.16 | <2E-16 |  | 0.011 | 3.55 | <2E-16 |
| GL | Genotypes (G) | 158 | 4.223 | 442.71 | <2E-16 |  | 3.646 | 181.77 | <2E-16 |
|  | Environment (E) | 1 | 0.062 | 6.48 | 1.11E-2 |  | 0.531 | 26.47 | 3.67E-07 |
|  | G×E interactions | 158 | 0.039 | 4.14 | <2E-16 |  | 0.059 | 2.95 | <2E-16 |
| TGW | Genotypes (G) | 158 | 51.950 | 70.96 | <2E-16 |  | 45.100 | 19.35 | <2E-16 |
|  | Environment (E) | 1 | 2.560 | 3.50 | 6.18E-2 |  | 74.740 | 32.07 | 2.35E-08 |
|  | G×E interactions | 158 | 1.880 | 2.57 | <2E-16 |  | 4.490 | 1.93 | 2.03E-08 |

GW: grain width; GL: grain length; TGW: thousand grain weight

**Table S3 Main-effect QTN for grain width in main crop of 159 rice accessions in environment 1**

| **No.** | **Marker** | **Chr** | **Position (bp)** | **LOD score** | **Additive** | **Variance** | **r^2^(%)** | **P-value** | **Significance** |
| --- | --- | --- | --- | --- | --- | --- | --- | --- | --- |
| ***Significant QTNs at the critical P-value of 2.48e-08 (=0.05/m, where m is the number of markers)*** | | | | | | | | | |
| 1 | 204944049 | 2 | 4944049 | 9.7150 | 0.0403 | 0.0016 | 1.4413 | 2.253E-11 | SIG |
| 2 | 302248120 | 3 | 2248120 | 12.5121 | -0.0471 | 0.0021 | 1.9516 | 3.1823E-14 | SIG |
| 3 | 316321816 | 3 | 16321816 | 9.1294 | 0.0397 | 0.0015 | 1.3923 | 8.9395E-11 | SIG |
| 4 | 328864309 | 3 | 28864309 | 16.0879 | -0.0553 | 0.0023 | 2.0914 | 8.1781E-17 | SIG |
| 5 | 331987897 | 3 | 31987897 | 8.9685 | -0.0393 | 0.0015 | 1.3932 | 1.0761E-09 | SIG |
| 6 | 505361276 | 5 | 5361276 | 28.8742 | -0.0813 | 0.0058 | 5.2635 | 9.1988E-31 | SIG |
| 7 | 506052088 | 5 | 6052088 | 7.5556 | -0.0349 | 0.0012 | 1.0923 | 3.6658E-09 | SIG |
| 8 | 508302651 | 5 | 8302651 | 10.2630 | -0.0415 | 0.0014 | 1.2897 | 6.2126E-12 | SIG |
| 9 | 707540563 | 7 | 7540563 | 15.6636 | -0.0545 | 0.0013 | 1.1757 | 2.014E-17 | SIG |
| 10 | 713191307 | 7 | 13191307 | 23.4228 | 0.0733 | 0.0012 | 1.0935 | 3.7853E-24 | SIG |
| 11 | 724208049 | 7 | 24208049 | 24.8446 | 0.0732 | 0.0020 | 1.7910 | 1.0601E-26 | SIG |
| 12 | 907639381 | 9 | 7639381 | 27.2761 | 0.0792 | 0.0024 | 2.1483 | 3.749E-29 | SIG |
| 13 | 1004780228 | 10 | 4780228 | 11.1093 | 0.0435 | 0.0015 | 1.3368 | 8.5215E-13 | SIG |
| 14 | 1117401993 | 11 | 17401993 | 29.6568 | -0.0832 | 0.0015 | 1.3662 | 1.4979E-31 | SIG |
| 15 | 1122278809 | 11 | 22278809 | 18.5012 | -0.0595 | 0.0015 | 1.3516 | 2.6992E-20 | SIG |
| 16 | 1126036677 | 11 | 26036677 | 19.5043 | -0.0619 | 0.0033 | 2.9915 | 2.6117E-21 | SIG |
| 17 | 1128029610 | 11 | 28029610 | 30.5384 | 0.0896 | 0.0009 | 0.8092 | 2.9022E-31 | SIG |
| 18 | 1211103678 | 12 | 11103678 | 14.0670 | 0.0502 | 0.0023 | 2.0812 | 8.3808E-16 | SIG |
| ***Suggested QTNs with the LOD score ≥ 3.0 but the P-value > 0.05/m, where m is the number of markers*** | | | | | | | | | |
| 1 | 1005603387 | 10 | 5603387 | 3.3402 | 0.0225 | 0.0004 | 0.3348 | 8.78323E-05 | SUG |
| 2 | 1005711159 | 10 | 5711159 | 3.3402 | 0.0225 | 0.0004 | 0.3348 | 8.78323E-05 | SUG |

**Table S4 Main-effect QTN for grain length in main crop of 159 rice accessions in environment 1**

| **No.** | **Marker** | **Chr** | **Position (bp)** | **LOD score** | **Additive** | **Variance** | **r^2^(%)** | **P-value** | **Significance** |
| --- | --- | --- | --- | --- | --- | --- | --- | --- | --- |
| ***Significant QTNs at the critical P-value of 2.48e-08 (=0.05/m, where m is the number of markers)*** | | | | | | | | | |
| 1 | 119393842 | 1 | 19393842 | 11.3069 | -0.1109 | 0.0065 | 0.901 | 5.35991E-13 | SIG |
| 2 | 134736531 | 1 | 34736531 | 16.2889 | 0.0529 | 0.0127 | 1.7513 | 5.14851E-17 | SIG |
| 3 | 202085514 | 2 | 2085514 | 14.7434 | 0.1301 | 0.0084 | 1.1566 | 1.72565E-16 | SIG |
| 4 | 300616193 | 3 | 616193 | 37.0447 | 0.2480 | 0.0186 | 2.5656 | 5.49642E-39 | SIG |
| 5 | 316179400 | 3 | 16179400 | 18.3827 | 0.1494 | 0.0072 | 0.9907 | 3.55737E-20 | SIG |
| 6 | 316746142 | 3 | 16746142 | 40.2497 | 0.2648 | 0.0671 | 9.2713 | 3.29183E-42 | SIG |
| 7 | 401054013 | 4 | 1054013 | 24.4785 | -0.1845 | 0.0128 | 1.7622 | 2.4809E-26 | SIG |
| 8 | 605597086 | 6 | 5597086 | 14.9649 | -0.1317 | 0.0165 | 2.283 | 1.08559E-15 | SIG |
| 9 | 724964429 | 7 | 24964429 | 26.2230 | -0.1906 | 0.0110 | 1.5165 | 4.31965E-28 | SIG |
| 10 | 822125056 | 8 | 22125056 | 11.7954 | -0.1163 | 0.0121 | 1.6667 | 1.7053E-13 | SIG |
| 11 | 915356676 | 9 | 15356676 | 18.6014 | 0.1551 | 0.0132 | 1.8236 | 2.50761E-19 | SIG |
| 12 | 1101936516 | 11 | 1936516 | 17.8416 | 0.1519 | 0.0038 | 0.5279 | 1.44243E-18 | SIG |
| 13 | 1120929766 | 11 | 20929766 | 14.4766 | -0.1294 | 0.0044 | 0.6032 | 3.21804E-16 | SIG |
| 14 | 1126213773 | 11 | 26213773 | 25.7754 | -0.1890 | 0.0350 | 4.8415 | 1.68087E-26 | SIG |
| 15 | 1204438901 | 12 | 4438901 | 10.6687 | -0.1072 | 0.0099 | 1.3697 | 2.39604E-12 | SIG |
| 16 | 1222587924 | 12 | 22587924 | 16.8888 | 0.1423 | 0.0111 | 1.5373 | 1.29356E-17 | SIG |
| 17 | 1227081846 | 12 | 27081846 | 7.8580 | 0.0904 | 0.0069 | 0.9546 | 1.7934E-09 | SIG |
| ***Suggested QTNs with the LOD score ≥ 3.0 but the P-value > 0.05/m, where m is the number of markers*** | | | | | | | | | |
| 1 | 1200776662 | 12 | 776662 | 5.4371 | 0.0736 | 0.0052 | 0.7120 | 5.622E-07 | SUG |

**Table S5 Main-effect QTN for thousand grain weight in main crop of 159 rice accessions in environment 1**

| **No.** | **Marker** | **Chr** | **Position (bp)** | **LOD score** | **Additive** | **Variance** | **r^2^(%)** | **P-value** | **Significance** |
| --- | --- | --- | --- | --- | --- | --- | --- | --- | --- |
| ***Significant QTNs at the critical P-value of 2.48e-08 (=0.05/m, where m is the number of markers)*** | | | | | | | | | |
| 1 | 221664294 | 2 | 21664294 | 14.7882 | -0.8023 | 0.3102 | 3.4237 | 1.5543E-16 | SIG |
| 2 | 231404034 | 2 | 31404034 | 12.6891 | 0.7266 | 0.3474 | 3.8333 | 2.1029E-14 | SIG |
| 3 | 431939665 | 4 | 31939665 | 26.4905 | -1.1749 | 0.4169 | 4.6008 | 2.3215E-28 | SIG |
| 4 | 501479400 | 5 | 1479400 | 10.4807 | 0.6493 | 0.3422 | 3.7766 | 3.7261E-12 | SIG |
| 5 | 513854833 | 5 | 13854833 | 9.2282 | 0.6035 | 0.3625 | 4.0004 | 7.0824E-11 | SIG |
| 6 | 603189214 | 6 | 3189214 | 9.6893 | -0.6285 | 0.1851 | 2.0426 | 2.3932E-11 | SIG |
| 7 | 701146652 | 7 | 1146652 | 14.0739 | -0.7784 | 0.4525 | 4.9932 | 8.2467E-16 | SIG |
| 8 | 702651150 | 7 | 2651150 | 31.3033 | -1.3373 | 0.4292 | 4.7361 | 3.292E-33 | SIG |
| 9 | 825154283 | 8 | 25154283 | 13.0226 | 0.7380 | 0.2887 | 3.1858 | 9.6371E-15 | SIG |
| 10 | 1001426946 | 10 | 1426946 | 21.0852 | -1.0067 | 0.3078 | 3.3972 | 6.6004E-23 | SIG |
| 11 | 1103505182 | 11 | 3505182 | 11.7410 | 0.6983 | 0.1584 | 1.7482 | 1.9376E-13 | SIG |
| 12 | 1122137790 | 11 | 22137790 | 11.7666 | -0.6970 | 0.1364 | 1.5049 | 1.8245E-13 | SIG |
| 13 | 1215799876 | 12 | 15799876 | 8.6810 | -0.5830 | 0.1801 | 1.9878 | 2.5708E-10 | SIG |
| ***Suggested QTNs with the LOD score ≥ 3.0 but the P-value > 0.05/m, where m is the number of markers*** | | | | | | | | | |
| 1 | 230953174 | 2 | 30953174 | 6.3746 | 0.4957 | 0.2279 | 2.5148 | 6.026E-08 | SUG |
| 2 | 601540336 | 6 | 1540336 | 4.8293 | 0.4225 | 0.1733 | 1.9127 | 2.4071E-06 | SUG |
| 3 | 1118164358 | 11 | 18164358 | 6.1153 | 0.2351 | 0.2281 | 2.5169 | 7.6716E-07 | SUG |

**Table S6 Main-effect QTN for grain width in ratoon rice of 159 rice accessions in environment 1**

| **No.** | **Marker** | **Chr** | **Position (bp)** | **LOD score** | **Additive** | **Variance** | **r^2^(%)** | **P-value** | **Significance** |
| --- | --- | --- | --- | --- | --- | --- | --- | --- | --- |
| ***Significant QTNs at the critical P-value of 2.48e-08 (=0.05/m, where m is the number of markers)*** | | | | | | | | | |
| 1 | 204994560 | 2 | 4994560 | 8.5292 | -0.0340 | 0.0012 | 1.3679 | 3.6779E-10 | SIG |
| 2 | 205481928 | 2 | 5481928 | 16.4204 | -0.0500 | 0.0021 | 2.4642 | 3.8033E-17 | SIG |
| 3 | 229241853 | 2 | 29241853 | 10.8223 | 0.0398 | 0.0007 | 0.7846 | 1.6711E-12 | SIG |
| 4 | 313863861 | 3 | 13863861 | 14.5771 | -0.0465 | 0.0013 | 1.5694 | 2.5451E-16 | SIG |
| 5 | 327973483 | 3 | 27973483 | 22.7161 | -0.0634 | 0.0005 | 0.6190 | 1.9262E-23 | SIG |
| 6 | 335262383 | 3 | 35262383 | 12.4362 | 0.0429 | 0.0018 | 2.1607 | 3.802E-14 | SIG |
| 7 | 429978124 | 4 | 29978124 | 21.9022 | 0.0602 | 0.0007 | 0.8262 | 1.255E-22 | SIG |
| 8 | 505357438 | 5 | 5357438 | 32.9949 | -0.0816 | 0.0057 | 6.7909 | 6.5258E-35 | SIG |
| 9 | 906924345 | 9 | 6924345 | 10.9581 | 0.0392 | 0.0006 | 0.7318 | 1.215E-12 | SIG |
| 10 | 1003262163 | 10 | 3262163 | 30.8766 | 0.0785 | 0.0006 | 0.7324 | 1.332E-31 | SIG |
| 11 | 1107066369 | 11 | 7066369 | 13.5828 | -0.0445 | 0.0007 | 0.8037 | 2.5993E-15 | SIG |
| 12 | 1107818816 | 11 | 7818816 | 8.1079 | -0.0331 | 0.0009 | 1.0146 | 9.9384E-10 | SIG |
| 13 | 1116768397 | 11 | 16768397 | 34.4062 | -0.084 | 0.0019 | 2.2670 | 3.9359E-35 | SIG |
| 14 | 1118748365 | 11 | 18748365 | 12.9745 | -0.0433 | 0.0014 | 1.7036 | 1.0785E-14 | SIG |
| 15 | 1125125749 | 11 | 25125749 | 8.7264 | -0.0344 | 0.0007 | 0.8369 | 2.3103E-10 | SIG |
| 16 | 1212716690 | 12 | 12716690 | 19.1870 | 0.0556 | 0.0028 | 3.3706 | 5.4671E-21 | SIG |
| ***Suggested QTNs with the LOD score ≥ 3.0 but the P-value > 0.05/m, where m is the number of markers*** | | | | | | | | | |
| 1 | 3002278621 | 3 | 850101 | 4.4152 | -0.0240 | 0.0004 | 0.5229 | 6.5104E-06 | SUG |
| 2 | 505323603 | 5 | 5323603 | 7.4433 | 0.0322 | 0.0010 | 1.2248 | 3.606E-08 | SUG |
| 3 | 1004502295 | 10 | 4502295 | 6.7386 | 0.0303 | 0.0007 | 0.8832 | 2.5389E-08 | SUG |
| 4 | 1203761337 | 12 | 3761337 | 6.4363 | 0.0292 | 0.0007 | 0.8495 | 5.2039E-08 | SUG |

**Table S7 Main-effect QTN for grain length in ratoon rice of 159 rice accessions in environment 1**

| **No.** | **Marker** | **Chr** | **Position (bp)** | **LOD score** | **Additive** | **Variance** | **r^2^(%)** | **P-value** | **Significance** |
| --- | --- | --- | --- | --- | --- | --- | --- | --- | --- |
| ***Significant QTNs at the critical P-value of 2.48e-08 (=0.05/m, where m is the number of markers)*** | | | | | | | | | |
| 1 | 125212877 | 1 | 25212877 | 15.0073 | -0.1469 | 0.0074 | 1.179 | 9.3192E-17 | SIG |
| 2 | 316708508 | 3 | 16708508 | 32.7223 | 0.2495 | 0.0599 | 9.5155 | 1.2274E-34 | SIG |
| 3 | 334087282 | 3 | 34087282 | 16.4269 | 0.1549 | 0.0046 | 0.7291 | 3.3938E-18 | SIG |
| 4 | 401015786 | 4 | 1015786 | 14.1433 | -0.1412 | 0.0091 | 1.4534 | 7.0114E-16 | SIG |
| 5 | 403785259 | 4 | 3785259 | 23.2110 | -0.1949 | 0.0107 | 1.6956 | 4.7141E-25 | SIG |
| 6 | 425746858 | 4 | 25746858 | 15.3770 | -0.1491 | 0.0222 | 3.5259 | 3.9313E-17 | SIG |
| 7 | 518384394 | 5 | 18384394 | 12.3576 | -0.1310 | 0.0158 | 2.5045 | 4.5694E-14 | SIG |
| 8 | 616645152 | 6 | 16645152 | 7.7001 | -0.0998 | 0.0088 | 1.4037 | 2.6047E-09 | SIG |
| 9 | 724800887 | 7 | 24800887 | 10.7332 | -0.1202 | 0.0056 | 0.8823 | 2.0594E-12 | SIG |
| 10 | 1018229059 | 10 | 18229059 | 10.4415 | -0.1179 | 0.0092 | 1.4545 | 4.0852E-12 | SIG |
| 11 | 1211562787 | 12 | 11562787 | 9.7869 | -0.1147 | 0.0054 | 0.8538 | 1.9024E-11 | SIG |
| 12 | 1214087199 | 12 | 14087199 | 14.2623 | 0.1510 | 0.0122 | 1.9349 | 5.4734E-15 | SIG |
| 13 | 1227144058 | 12 | 27144058 | 8.6598 | 0.1086 | 0.0106 | 1.6826 | 2.1904E-09 | SIG |
| ***Suggested QTNs with the LOD score ≥ 3.0 but the P-value > 0.05/m, where m is the number of markers*** | | | | | | | | | |
| 1 | 204612650 | 2 | 4612650 | 6.4293 | -0.0899 | 0.0078 | 1.244 | 3.7237E-07 | SUG |
| 2 | 321188351 | 3 | 21188351 | 6.3821 | -0.0894 | 0.008 | 1.2699 | 5.9189E-08 | SUG |
| 3 | 1200945154 | 12 | 945154 | 5.8410 | 0.0855 | 0.004 | 0.6386 | 2.1452E-07 | SUG |

**Table S8 Main-effect QTN for thousand grain weight in ratoon rice of 159 rice accessions in environment 1**

| **No.** | **Marker** | **Chr** | **Position (bp)** | **LOD score** | **Additive** | **Variance** | **r^2^(%)** | **P-value** | **Significance** |
| --- | --- | --- | --- | --- | --- | --- | --- | --- | --- |
| ***Significant QTNs at the critical P-value of 2.48e-08 (=0.05/m, where m is the number of markers)*** | | | | | | | | | |
| 1 | 132257504 | 1 | 32257504 | 18.2971 | 0.9231 | 0.2314 | 2.4276 | 5.0536E-19 | SIG |
| 2 | 224702111 | 2 | 24702111 | 10.6541 | 0.6407 | 0.1806 | 1.8943 | 2.48E-12 | SIG |
| 3 | 226049877 | 2 | 26049877 | 17.1879 | -0.8747 | 0.2003 | 2.1016 | 6.4969E-18 | SIG |
| 4 | 228749717 | 2 | 28749717 | 15.3904 | 0.8012 | 0.1663 | 1.7447 | 3.8098E-17 | SIG |
| 5 | 231234763 | 2 | 31234763 | 10.9198 | 0.6500 | 0.296 | 3.105 | 1.3293E-12 | SIG |
| 6 | 316766278 | 3 | 16766278 | 7.7666 | 0.5386 | 0.2663 | 2.7935 | 2.226E-09 | SIG |
| 7 | 404570606 | 4 | 4570606 | 24.2396 | -1.0752 | 0.4415 | 4.6318 | 4.3209E-26 | SIG |
| 8 | 431723084 | 4 | 31723084 | 29.7296 | -1.2454 | 0.3995 | 4.1909 | 1.2651E-31 | SIG |
| 9 | 613946862 | 6 | 13946862 | 9.0655 | -0.6048 | 0.2332 | 2.4468 | 8.6072E-10 | SIG |
| 10 | 819614457 | 8 | 19614457 | 8.0206 | -0.5562 | 0.2143 | 2.2482 | 9.5429E-09 | SIG |
| 11 | 1000463104 | 10 | 463104 | 16.0027 | -0.8516 | 0.2053 | 2.1535 | 9.9509E-17 | SIG |
| 12 | 1010606927 | 10 | 10606927 | 13.5255 | 0.7403 | 0.4212 | 4.4191 | 2.9722E-15 | SIG |
| 13 | 1103384367 | 11 | 3384367 | 9.1131 | 0.5875 | 0.1257 | 1.3183 | 9.2873E-11 | SIG |
| 14 | 1110422612 | 11 | 10422612 | 12.5886 | -0.7091 | 0.108 | 1.1333 | 2.6607E-14 | SIG |
| 15 | 1113977759 | 11 | 13977759 | 9.6315 | 0.5779 | 0.3646 | 3.8243 | 2.3383E-10 | SIG |
| 16 | 1117131649 | 11 | 17131649 | 13.3134 | 0.7316 | 0.1738 | 1.8235 | 4.8649E-14 | SIG |
| ***Suggested QTNs with the LOD score ≥ 3.0 but the P-value > 0.05/m, where m is the number of markers*** | | | | | | | | | |
| 1 | 505895833 | 5 | 5895833 | 6.3175 | 0.4775 | 0.2265 | 2.376 | 6.9026E-08 | SUG |
| 2 | 806110721 | 8 | 6110721 | 6.1099 | -0.4669 | 0.2149 | 2.2541 | 7.7686E-07 | SUG |

**Table S9 Main-effect QTN for grain width in main crop of 159 rice accessions in environment 2**

| **No.** | **Marker** | **Chr** | **Position (bp)** | **LOD score** | **Additive** | **Variance** | **r^2^(%)** | **P-value** | **Significance** |
| --- | --- | --- | --- | --- | --- | --- | --- | --- | --- |
| ***Significant QTNs at the critical P-value of 2.48e-08 (=0.05/m, where m is the number of markers)*** | | | | | | | | | |
| 1 | 105991947 | 1 | 5991947 | 13.1902 | 0.0669 | 0.0027 | 2.3651 | 6.4611E-14 | SIG |
| 2 | 223208718 | 2 | 23208718 | 20.8751 | -0.0846 | 0.0017 | 1.4798 | 1.076E-22 | SIG |
| 3 | 324648339 | 3 | 24648339 | 9.1433 | -0.0520 | 0.0026 | 2.2792 | 8.6515E-11 | SIG |
| 4 | 505358771 | 5 | 5358771 | 44.2078 | -0.1497 | 0.0199 | 17.2873 | 6.2205E-45 | SIG |
| 5 | 703682234 | 7 | 3682234 | 11.3492 | -0.0582 | 0.0010 | 0.841 | 4.4789E-12 | SIG |
| 6 | 724771358 | 7 | 24771358 | 18.0897 | 0.0768 | 0.0023 | 1.9638 | 7.0391E-20 | SIG |
| 7 | 915748352 | 9 | 15748352 | 21.2544 | 0.0894 | 0.0014 | 1.2543 | 5.5766E-22 | SIG |
| 8 | 1118748365 | 11 | 18748365 | 15.0925 | -0.0686 | 0.0036 | 3.1333 | 7.6382E-17 | SIG |
| 9 | 1126036677 | 11 | 26036677 | 7.5017 | -0.0459 | 0.0018 | 1.5758 | 4.1643E-09 | SIG |
| 10 | 1211103678 | 12 | 11103678 | 11.9365 | 0.0597 | 0.0032 | 2.8166 | 1.2253E-13 | SIG |
| ***Suggested QTNs with the LOD score ≥ 3.0 but the P-value > 0.05/m, where m is the number of markers*** | | | | | | | | | |
| 1 | 140426419 | 1 | 40426419 | 6.1663 | -0.0409 | 0.0017 | 1.4472 | 6.8215E-07 | SUG |
| 2 | 335144023 | 3 | 35144023 | 6.9298 | 0.0446 | 0.0017 | 1.4528 | 1.1761E-07 | SUG |

**Table S10 Main-effect QTN for grain length in main crop of 159 rice accessions in environment 2**

| **No.** | **Marker** | **Chr** | **Position (bp)** | **LOD score** | **Additive** | **Variance** | **r^2^(%)** | **P-value** | **Significance** |
| --- | --- | --- | --- | --- | --- | --- | --- | --- | --- |
| ***Significant QTNs at the critical P-value of 2.48e-08 (=0.05/m, where m is the number of markers)*** | | | | | | | | | |
| 1 | 109828171 | 1 | 9828171 | 25.5362 | -0.1864 | 0.0067 | 0.9579 | 2.13E-27 | SIG |
| 2 | 119393842 | 1 | 19393842 | 12.5261 | -0.1174 | 0.0073 | 1.0479 | 3.08E-14 | SIG |
| 3 | 130176332 | 1 | 30176332 | 15.6156 | -0.1351 | 0.0067 | 0.9585 | 2.25E-17 | SIG |
| 4 | 212917223 | 2 | 12917223 | 13.1978 | -0.0626 | 0.0138 | 1.9770 | 6.35E-14 | SIG |
| 5 | 224992114 | 2 | 24992114 | 13.5568 | -0.1235 | 0.0052 | 0.7519 | 2.76E-15 | SIG |
| 6 | 316708508 | 3 | 16708508 | 47.9439 | 0.3079 | 0.0912 | 13.0860 | 6.11E-50 | SIG |
| 7 | 400960933 | 4 | 960933 | 13.6822 | -0.1238 | 0.0055 | 0.7958 | 2.06E-15 | SIG |
| 8 | 505357676 | 5 | 5357676 | 10.4595 | 0.1059 | 0.0098 | 1.4074 | 3.92E-12 | SIG |
| 9 | 606503404 | 6 | 6503404 | 11.6161 | 0.1123 | 0.0105 | 1.5058 | 2.60E-13 | SIG |
| 10 | 724206745 | 7 | 24206745 | 10.2968 | -0.1050 | 0.0083 | 1.1954 | 5.74E-12 | SIG |
| 11 | 901068639 | 9 | 1068639 | 7.7969 | -0.0908 | 0.0048 | 0.6839 | 2.07E-09 | SIG |
| 12 | 915356676 | 9 | 15356676 | 13.5136 | 0.1245 | 0.0073 | 1.0410 | 3.07E-14 | SIG |
| 13 | 1117830860 | 11 | 17830860 | 9.6088 | 0.1006 | 0.0096 | 1.3802 | 2.89E-11 | SIG |
| 14 | 1204438901 | 12 | 4438901 | 10.2763 | -0.1045 | 0.0094 | 1.3513 | 6.02E-12 | SIG |
| 15 | 1214201832 | 12 | 14201832 | 10.5301 | -0.1077 | 0.0091 | 1.3005 | 3.32E-12 | SIG |
| 16 | 1218051198 | 12 | 18051198 | 27.4850 | -0.1958 | 0.0139 | 1.9912 | 2.31E-29 | SIG |
| 17 | 1224188617 | 12 | 24188617 | 7.4421 | -0.0871 | 0.0071 | 1.0150 | 4.79E-09 | SIG |
| 18 | 1227064916 | 12 | 27064916 | 8.6186 | 0.0948 | 0.0075 | 1.0782 | 2.98E-10 | SIG |
| ***Suggested QTNs with the LOD score ≥ 3.0 but the P-value > 0.05/m, where m is the number of markers*** | | | | | | | | | |
| 1 | 335504491 | 3 | 35504491 | 5.2844 | -0.0727 | 0.0048 | 0.6834 | 8.0969E-07 | SUG |
| 2 | 1223254141 | 12 | 23254141 | 5.9459 | 0.0770 | 0.0034 | 0.4930 | 1.6708E-07 | SUG |

**Table S11 Main-effect QTN for thousand grain weight in main crop of 159 rice accessions in environment 2**

| **No.** | **Marker** | **Chr** | **Position (bp)** | **LOD score** | **Additive** | **Variance** | **r^2^(%)** | **P-value** | **Significance** |
| --- | --- | --- | --- | --- | --- | --- | --- | --- | --- |
| ***Significant QTNs at the critical P-value of 2.48e-08 (=0.05/m, where m is the number of markers)*** | | | | | | | | | |
| 1 | 113552450 | 1 | 13552450 | 6.8499 | 0.4231 | 0.1666 | 1.8761 | 1.95E-08 | SIG |
| 2 | 130072714 | 1 | 30072714 | 13.1581 | 0.6015 | 0.0853 | 0.9603 | 7.02E-15 | SIG |
| 3 | 139719661 | 1 | 39719661 | 13.5463 | -0.618 | 0.1707 | 1.9224 | 2.83E-15 | SIG |
| 4 | 208196020 | 2 | 8196020 | 13.1920 | 0.6082 | 0.1991 | 2.2418 | 6.48E-15 | SIG |
| 5 | 225079124 | 2 | 25079124 | 17.1510 | -0.7175 | 0.1126 | 1.2678 | 6.27E-19 | SIG |
| 6 | 410654189 | 4 | 10654189 | 14.4739 | 0.6434 | 0.1353 | 1.5229 | 3.24E-16 | SIG |
| 7 | 433256631 | 4 | 33256631 | 22.6513 | -0.8531 | 0.3621 | 4.0762 | 1.73E-24 | SIG |
| 8 | 522017452 | 5 | 22017452 | 10.2685 | -0.5114 | 0.1615 | 1.8179 | 5.39E-11 | SIG |
| 9 | 603189214 | 6 | 3189214 | 18.2820 | -0.7473 | 0.2617 | 2.9461 | 4.50E-20 | SIG |
| 10 | 707640833 | 7 | 7640833 | 16.5078 | -0.6915 | 0.2192 | 2.4683 | 2.81E-18 | SIG |
| 11 | 718517653 | 7 | 18517653 | 35.1149 | -1.1750 | 0.3255 | 3.6644 | 7.70E-36 | SIG |
| 12 | 817265592 | 8 | 17265592 | 19.6740 | 0.7798 | 0.6141 | 6.9137 | 2.12E-20 | SIG |
| 13 | 1009402611 | 10 | 9402611 | 17.7644 | 0.6798 | 0.5194 | 5.8478 | 1.72E-18 | SIG |
| 14 | 1110907638 | 11 | 10907638 | 15.3171 | 0.6749 | 0.4527 | 5.0967 | 4.52E-17 | SIG |
| 15 | 1200424245 | 12 | 424245 | 14.5405 | -0.6355 | 0.4078 | 4.5913 | 2.88E-15 | SIG |
| 16 | 1200591787 | 12 | 591787 | 14.1359 | -0.2648 | 0.2811 | 3.1642 | 7.32E-15 | SIG |
| 17 | 1210698465 | 12 | 10698465 | 17.6456 | -0.7353 | 0.3738 | 4.2085 | 1.98E-19 | SIG |
| ***Suggested QTNs with the LOD score ≥ 3.0 but the P-value > 0.05/m, where m is the number of markers*** | | | | | | | | | |
| 1 | 100800544 | 1 | 800544 | 5.9737 | -0.3838 | 0.0781 | 0.8791 | 1.5636E-07 | SUG |

**Table S12 Main-effect QTN for grain width in ratoon rice of 159 rice accessions in environment 2**

| **No.** | **Marker** | **Chr** | **Position (bp)** | **LOD score** | **Additive** | **Variance** | **r^2^(%)** | **P-value** | **Significance** |
| --- | --- | --- | --- | --- | --- | --- | --- | --- | --- |
| ***Significant QTNs at the critical P-value of 2.48e-08 (=0.05/m, where m is the number of markers)*** | | | | | | | | | |
| 1 | 217616900 | 2 | 17616900 | 11.0079 | 0.0396 | 0.0005 | 0.6547 | 9.829E-12 | SIG |
| 2 | 225565194 | 2 | 25565194 | 22.5009 | -0.0603 | 0.0009 | 1.1252 | 2.455E-24 | SIG |
| 3 | 325165431 | 3 | 25165431 | 13.6068 | 0.0430 | 0.0017 | 2.2313 | 2.4576E-15 | SIG |
| 4 | 335144023 | 3 | 35144023 | 7.9619 | 0.0327 | 0.0009 | 1.202 | 1.0925E-08 | SIG |
| 5 | 404722231 | 4 | 4722231 | 24.4147 | -0.0627 | 0.0018 | 2.3024 | 2.877E-26 | SIG |
| 6 | 505358771 | 5 | 5358771 | 21.4303 | -0.0575 | 0.0029 | 3.754 | 3.7193E-22 | SIG |
| 7 | 506015422 | 5 | 6015422 | 7.5654 | -0.0312 | 0.0010 | 1.228 | 3.5817E-09 | SIG |
| 8 | 513900081 | 5 | 13900081 | 9.2967 | 0.0344 | 0.0012 | 1.499 | 6.0283E-11 | SIG |
| 9 | 521088250 | 5 | 21088250 | 9.3444 | -0.0346 | 0.0006 | 0.8102 | 4.5287E-10 | SIG |
| 10 | 710495471 | 7 | 10495471 | 24.0154 | -0.0624 | 0.0011 | 1.4033 | 7.2735E-26 | SIG |
| 11 | 725284287 | 7 | 25284287 | 29.0213 | 0.0720 | 0.0013 | 1.6967 | 9.544E-30 | SIG |
| 12 | 805984678 | 8 | 5984678 | 17.3524 | 0.0506 | 0.0018 | 2.2875 | 3.9233E-19 | SIG |
| 13 | 808915046 | 8 | 8915046 | 11.3178 | -0.0388 | 0.0014 | 1.7228 | 4.8157E-12 | SIG |
| 14 | 907342896 | 9 | 7342896 | 8.0760 | 0.0320 | 0.0005 | 0.5795 | 1.0717E-09 | SIG |
| 15 | 914471049 | 9 | 14471049 | 11.3938 | 0.0378 | 0.0008 | 1.0372 | 4.0423E-12 | SIG |
| 16 | 1117881763 | 11 | 17881763 | 50.6026 | -0.1134 | 0.0022 | 2.7878 | 2.5078E-51 | SIG |
| 17 | 1125735896 | 11 | 25735896 | 27.5050 | -0.0694 | 0.0026 | 3.3408 | 2.2045E-29 | SIG |
| 18 | 1126041008 | 11 | 26041008 | 13.2197 | 0.0425 | 0.0018 | 2.305 | 6.0763E-15 | SIG |
| 19 | 1203761318 | 12 | 3761318 | 9.2340 | 0.0347 | 0.0010 | 1.2982 | 6.9866E-11 | SIG |
| 20 | 1212532451 | 12 | 12532451 | 11.9395 | 0.0394 | 0.0010 | 1.3327 | 1.1507E-12 | SIG |

**Table S13 Main-effect QTN for grain length in ratoon rice of 159 rice accessions in environment 2**

| **No.** | **Marker** | **Chr** | **Position (bp)** | **LOD score** | **Additive** | **Variance** | **r^2^(%)** | **P-value** | **Significance** |
| --- | --- | --- | --- | --- | --- | --- | --- | --- | --- |
| ***Significant QTNs at the critical P-value of 2.48e-08 (=0.05/m, where m is the number of markers)*** | | | | | | | | | |
| 1 | 119393842 | 1 | 19393842 | 7.9068 | -0.1023 | 0.0055 | 0.8410 | 1.60E-09 | SIG |
| 2 | 125276755 | 1 | 25276755 | 10.0922 | -0.1190 | 0.0108 | 1.6445 | 9.28E-12 | SIG |
| 3 | 204007974 | 2 | 4007974 | 7.7872 | 0.1014 | 0.0097 | 1.466 | 2.12E-09 | SIG |
| 4 | 301294521 | 3 | 1294521 | 25.3655 | 0.2100 | 0.0104 | 1.5767 | 3.16E-27 | SIG |
| 5 | 316708508 | 3 | 16708508 | 35.5423 | 0.2704 | 0.0704 | 10.6726 | 1.78E-37 | SIG |
| 6 | 401015786 | 4 | 1015786 | 14.4574 | -0.1454 | 0.0097 | 1.4701 | 3.37E-16 | SIG |
| 7 | 402223635 | 4 | 2223635 | 17.0373 | 0.1642 | 0.0147 | 2.2351 | 8.18E-19 | SIG |
| 8 | 413696726 | 4 | 13696726 | 10.2757 | -0.1214 | 0.0076 | 1.1484 | 6.03E-12 | SIG |
| 9 | 435129844 | 4 | 35129844 | 12.4884 | -0.1335 | 0.0072 | 1.0917 | 3.36E-14 | SIG |
| 10 | 724214245 | 7 | 24214245 | 9.0692 | -0.1161 | 0.0081 | 1.2245 | 8.53E-10 | SIG |
| 11 | 820719718 | 8 | 20719718 | 14.5311 | -0.1482 | 0.0151 | 2.2922 | 2.83E-16 | SIG |
| 12 | 916419362 | 9 | 16419362 | 16.7360 | 0.1597 | 0.0082 | 1.2501 | 1.65E-18 | SIG |
| 13 | 1108501237 | 11 | 8501237 | 11.7475 | -0.1225 | 0.0075 | 1.1316 | 1.79E-12 | SIG |
| ***Suggested QTNs with the LOD score ≥ 3.0 but the P-value > 0.05/m, where m is the number of markers*** | | | | | | | | | |
| 1 | 606540542 | 6 | 6540542 | 3.6951 | 0.0678 | 0.0046 | 0.6931 | 3.71E-05 | SUG |
| 2 | 1108314878 | 11 | 8314878 | 6.3163 | 0.0903 | 0.0074 | 1.1201 | 6.92E-08 | SUG |
| 3 | 1117830860 | 11 | 17830860 | 4.9318 | 0.0790 | 0.0059 | 0.9008 | 1.88E-06 | SUG |

**Table S14 Main-effect QTN for thousand grain weight in ratoon rice of 159 rice accessions in environment 2**

| **No.** | **Marker** | **Chr** | **Position (bp)** | **LOD score** | **Additive** | **Variance** | **r^2^(%)** | **P-value** | **Significance** |
| --- | --- | --- | --- | --- | --- | --- | --- | --- | --- |
| ***Significant QTNs at the critical P-value of 2.48e-08 (=0.05/m, where m is the number of markers)*** | | | | | | | | | |
| 1 | 225079124 | 2 | 25079124 | 18.2466 | -0.9457 | 0.1956 | 2.4325 | 4.8844E-20 | SIG |
| 2 | 306596936 | 3 | 6596936 | 23.5944 | 1.1080 | 0.3426 | 4.2599 | 1.934E-25 | SIG |
| 3 | 330587749 | 3 | 30587749 | 6.7763 | 0.5210 | 0.1746 | 2.1704 | 2.3219E-08 | SIG |
| 4 | 431939665 | 4 | 31939665 | 12.4589 | -0.7399 | 0.1654 | 2.0559 | 3.6048E-14 | SIG |
| 5 | 501981355 | 5 | 1981355 | 24.5736 | 1.1433 | 0.3100 | 3.8540 | 1.9889E-26 | SIG |
| 6 | 506751886 | 5 | 6751886 | 8.5554 | -0.5990 | 0.2229 | 2.7717 | 3.4572E-10 | SIG |
| 7 | 511152752 | 5 | 11152752 | 12.6864 | -0.7406 | 0.1770 | 2.2001 | 2.0611E-13 | SIG |
| 8 | 611905299 | 6 | 11905299 | 22.5055 | -1.0805 | 0.3906 | 4.8568 | 3.1283E-23 | SIG |
| 9 | 628657023 | 6 | 28657023 | 9.8960 | 0.6441 | 0.4125 | 5.1286 | 1.2715E-10 | SIG |
| 10 | 807678664 | 8 | 7678664 | 16.2641 | -0.8679 | 0.2877 | 3.5773 | 4.9612E-18 | SIG |
| 11 | 1117131649 | 11 | 17131649 | 17.1211 | 0.9138 | 0.2607 | 3.2408 | 7.5781E-18 | SIG |
| 12 | 1118422045 | 11 | 18422045 | 8.6684 | -0.5996 | 0.3030 | 3.7672 | 2.1474E-09 | SIG |
| ***Suggested QTNs with the LOD score ≥ 3.0 but the P-value > 0.05/m, where m is the number of markers*** | | | | | | | | | |
| 1 | 140053361 | 1 | 40053361 | 4.6501 | 0.4262 | 0.1816 | 2.2584 | 3.7008E-06 | SUG |
| 2 | 316806894 | 3 | 16806894 | 4.9348 | 0.4386 | 0.1020 | 1.2677 | 1.8693E-06 | SUG |
| 3 | 422027451 | 4 | 22027451 | 6.7133 | 0.5249 | 0.2507 | 3.1171 | 1.9363E-07 | SUG |
| 4 | 1103476187 | 11 | 3476187 | 5.5165 | -0.4657 | 0.1328 | 1.6513 | 4.6506E-07 | SUG |

**Table S15 Main-effect QTNs interactions for grain width in main crop of 159 rice accessions in two environments using multi-environment joint analysis**

| **No.** | **Marker** | **Chr** | **Position (bp)** | **LOD score** | **Additive** | **Variance** | **r^2^(%)** | **P-value** | **Significance** |
| --- | --- | --- | --- | --- | --- | --- | --- | --- | --- |
| ***Significant QTNs at the critical P-value of 2.48e-08 (=0.05/m, where m is the number of markers)*** | | | | | | | | | |
| 1 | 138564970 | 1 | 38564970 | 40.8970 | 0.0531 | 0.0005 | 0.4667 | 1.272E-41 | SIG |
| 2 | 140502259 | 1 | 40502259 | 10.2573 | 0.0218 | 0.0005 | 0.4633 | 5.535E-11 | SIG |
| 3 | 201541597 | 2 | 1541597 | 26.9252 | -0.0394 | 0.0009 | 0.8008 | 8.4641E-29 | SIG |
| 4 | 313768754 | 3 | 13768754 | 34.2470 | -0.0457 | 0.0010 | 0.9194 | 3.5855E-36 | SIG |
| 5 | 316819050 | 3 | 16819050 | 11.3675 | -0.0246 | 0.0006 | 0.516 | 4.6505E-13 | SIG |
| 6 | 324648339 | 3 | 24648339 | 25.5588 | -0.0389 | 0.0015 | 1.3049 | 2.0188E-27 | SIG |
| 7 | 331660510 | 3 | 31660510 | 7.3262 | -0.0191 | 0.0004 | 0.3246 | 6.3089E-09 | SIG |
| 8 | 335144023 | 3 | 35144023 | 8.3243 | 0.0212 | 0.0004 | 0.3284 | 4.7421E-09 | SIG |
| 9 | 500636512 | 5 | 636512 | 14.6958 | 0.0277 | 0.0004 | 0.3979 | 2.017E-15 | SIG |
| 10 | 505361276 | 5 | 5361276 | 93.6886 | -0.0974 | 0.0083 | 7.4128 | 7.9129E-96 | SIG |
| 11 | 506213670 | 5 | 6213670 | 19.6598 | -0.0307 | 0.0010 | 0.9313 | 2.1926E-20 | SIG |
| 12 | 513788807 | 5 | 13788807 | 16.0072 | 0.0292 | 0.0008 | 0.7537 | 9.0326E-18 | SIG |
| 13 | 521087312 | 5 | 21087312 | 14.3008 | -0.0273 | 0.0002 | 0.1999 | 4.8529E-16 | SIG |
| 14 | 620319960 | 6 | 20319960 | 60.8151 | -0.0682 | 0.0011 | 0.9874 | 7.3095E-63 | SIG |
| 15 | 724401296 | 7 | 24401296 | 32.3148 | 0.0442 | 0.0005 | 0.4878 | 3.1563E-34 | SIG |
| 16 | 800487221 | 8 | 487221 | 30.8114 | -0.0429 | 0.0005 | 0.4653 | 1.548E-31 | SIG |
| 17 | 801495823 | 8 | 1495823 | 19.8533 | 0.0314 | 0.0007 | 0.6527 | 1.4042E-20 | SIG |
| 18 | 806060988 | 8 | 6060988 | 11.0279 | 0.0238 | 0.0006 | 0.4924 | 9.3858E-12 | SIG |
| 19 | 827378608 | 8 | 27378608 | 66.2065 | -0.0723 | 0.0011 | 0.9941 | 2.8472E-68 | SIG |
| 20 | 907514626 | 9 | 7514626 | 38.9908 | 0.0500 | 0.0010 | 0.9028 | 6.0692E-41 | SIG |
| 21 | 910216810 | 9 | 10216810 | 28.9976 | -0.0416 | 0.0003 | 0.2995 | 6.9094E-31 | SIG |
| 22 | 915748352 | 9 | 15748352 | 55.7970 | 0.0667 | 0.0007 | 0.6469 | 1.6035E-56 | SIG |
| 23 | 1022628897 | 10 | 22628897 | 18.6396 | 0.0317 | 0.0006 | 0.5763 | 2.2965E-19 | SIG |
| 24 | 1114234543 | 11 | 14234543 | 26.3805 | -0.0389 | 0.0004 | 0.3757 | 2.9971E-28 | SIG |
| 25 | 1118701082 | 11 | 18701082 | 35.4749 | -0.0467 | 0.0016 | 1.4135 | 2.0851E-37 | SIG |
| 26 | 1210350434 | 12 | 10350434 | 17.1268 | 0.0309 | 0.0008 | 0.7279 | 6.6376E-19 | SIG |
| 27 | 1215782282 | 12 | 15782282 | 8.2151 | -0.0203 | 0.0001 | 0.1267 | 6.0982E-09 | SIG |
| ***Suggested QTNs with the LOD score ≥ 3.0 but the P-value > 0.05/m, where m is the number of markers*** | | | | | | | | | |
| 1 | 219799308 | 2 | 19799308 | 6.6828 | 0.0185 | 0.0002 | 0.2068 | 2.077E-07 | SUG |
| 2 | 302278621 | 3 | 2278621 | 5.8835 | 0.0174 | 0.0003 | 0.2632 | 1.3084E-06 | SUG |
| 3 | 817656042 | 8 | 17656042 | 6.0548 | -0.0174 | 0.0003 | 0.2486 | 8.8182E-07 | SUG |
| 4 | 1212562266 | 12 | 12562266 | 6.3717 | 0.0177 | 0.0003 | 0.2553 | 6.0671E-08 | SUG |
| 5 | 1212651485 | 12 | 12651485 | 6.3717 | 0.0177 | 0.0003 | 0.2553 | 6.0671E-08 | SUG |

**Table S16 Main-effect QTNs and QTN-by-environment interactions for grain length in main crop of 159 rice accessions in two environments using multi-environment joint analysis**

| Main effect QTNs for grain length in main crop | | | | | |  | QTN-by-environment interactions for grain length in main crop | | | | | | |
| --- | --- | --- | --- | --- | --- | --- | --- | --- | --- | --- | --- | --- | --- |
| No. | Chr | Position (bp) | LOD (Q) | add | r^2^(%) |  | No. | Chr | Position (bp) | LOD (QE) | add*env1 | add*env2 | r^2^(%) |
| ***Significant QTNs at the critical P-value of 2.48e-08 (=0.05/m, where m is the number of markers)*** | | | | | |  | ***Suggested QTNs with the LOD score ≥ 3.0 but the P-value > 0.05/m, where m is the number of markers*** | | | | | | |
|  |  |  |  |  |  |  |  |  |  |  |  |  |  |
| 1 | 1 | 19393842 | 26.7024 | -0.0834 | 0.5202 |  | 1 | 3 | 30340995 | 6.3927 | -0.0378 | 0.0378 | 0.2016 |
| 2 | 1 | 36273012 | 12.0679 | 0.0523 | 0.1401 |  |  |  |  |  |  |  |  |
| 3 | 1 | 41721637 | 55.1168 | -0.1354 | 0.8621 |  |  |  |  |  |  |  |  |
| 4 | 2 | 4611151 | 15.4240 | -0.0526 | 0.4937 |  |  |  |  |  |  |  |  |
| 5 | 2 | 12917223 | 21.6944 | -0.0170 | 0.7721 |  |  |  |  |  |  |  |  |
| 6 | 2 | 24585913 | 48.8115 | -0.1230 | 0.4083 |  |  |  |  |  |  |  |  |
| 7 | 2 | 29971324 | 19.1238 | -0.0674 | 0.1273 |  |  |  |  |  |  |  |  |
| 8 | 3 | 616193 | 22.4779 | 0.0755 | 0.2431 |  |  |  |  |  |  |  |  |
| 9 | 3 | 16038474 | 51.1129 | 0.1271 | 0.7320 |  |  |  |  |  |  |  |  |
| 10 | 3 | 16708508 | 124.9527 | 0.2742 | 10.2135 |  |  |  |  |  |  |  |  |
| 11 | 3 | 23238238 | 44.2780 | -0.1160 | 0.2769 |  |  |  |  |  |  |  |  |
| 12 | 3 | 24589241 | 10.2696 | 0.0487 | 0.1221 |  |  |  |  |  |  |  |  |
| 13 | 4 | 607359 | 21.0166 | -0.0724 | 0.3920 |  |  |  |  |  |  |  |  |
| 14 | 4 | 4591488 | 49.9063 | 0.1249 | 0.9692 |  |  |  |  |  |  |  |  |
| 15 | 4 | 35129844 | 62.0264 | -0.1468 | 1.2292 |  |  |  |  |  |  |  |  |
| 16 | 5 | 5456085 | 33.3734 | 0.0957 | 0.7284 |  |  |  |  |  |  |  |  |
| 17 | 6 | 6534568 | 36.8136 | 0.1041 | 0.9205 |  |  |  |  |  |  |  |  |
| 18 | 7 | 14471382 | 16.6963 | -0.0671 | 0.1874 |  |  |  |  |  |  |  |  |
| 19 | 7 | 23874203 | 8.1755 | 0.0430 | 0.2567 |  |  |  |  |  |  |  |  |
| 20 | 7 | 24533051 | 12.6751 | -0.0548 | 0.1466 |  |  |  |  |  |  |  |  |
| 21 | 8 | 489575 | 18.5360 | 0.0675 | 0.2474 |  |  |  |  |  |  |  |  |
| 22 | 8 | 22125056 | 33.4626 | -0.098 | 1.2091 |  |  |  |  |  |  |  |  |
| 23 | 9 | 6053137 | 13.7134 | -0.0569 | 0.3262 |  |  |  |  |  |  |  |  |
| 24 | 9 | 7955689 | 22.0845 | -0.0724 | 0.7747 |  |  |  |  |  |  |  |  |
| 25 | 9 | 12884227 | 13.1748 | 0.0556 | 0.2004 |  |  |  |  |  |  |  |  |
| 26 | 11 | 17788225 | 28.2961 | 0.0859 | 1.0136 |  |  |  |  |  |  |  |  |
| 27 | 11 | 26213773 | 27.4812 | -0.0793 | 1.0188 |  |  |  |  |  |  |  |  |
| 28 | 12 | 4438901 | 12.6055 | -0.0543 | 0.3590 |  |  |  |  |  |  |  |  |
| 29 | 12 | 18076706 | 32.3876 | -0.0942 | 0.6669 |  |  |  |  |  |  |  |  |
| 30 | 12 | 22587924 | 54.807 | 0.1335 | 1.4548 |  |  |  |  |  |  |  |  |
| 31 | 12 | 24218710 | 17.5835 | -0.0653 | 0.5587 |  |  |  |  |  |  |  |  |
| 32 | 12 | 27092306 | 40.3835 | 0.1099 | 1.4291 |  |  |  |  |  |  |  |  |
| ***Suggested QTNs with the LOD score ≥ 3.0 but the P-value > 0.05/m, where m is the number of markers*** | | | | | |  |  |  |  |  |  |  |  |
| 1 | 2 | 5436687 | 7.2903 | -0.0203 | 0.1802 |  |  |  |  |  |  |  |  |

**Table S17 Main-effect QTNs and QTN-by-environment interactions for thousand grain weight in main crop of 159 rice accessions in two environments using multi-environment joint analysis**

| Main effect QTNs for thousand grain weight in main crop | | | | | |  | QTN-by-environment interactions for thousand grain weight in main crop | | | | | | |
| --- | --- | --- | --- | --- | --- | --- | --- | --- | --- | --- | --- | --- | --- |
| No. | Chr | Position (bp) | LOD (Q) | add | r^2^(%) |  | No. | Chr | Position (bp) | LOD (QE) | add*env1 | add*env2 | r^2^(%) |
| ***Significant QTNs at the critical P-value of 2.48e-08 (=0.05/m, where m is the number of markers)*** | | | | | |  | ***Significant QTNs at the critical P-value of 2.48e-08 (=0.05/m, where m is the number of markers)*** | | | | | | |
|  |  |  |  |  |  |  |  |  |  |  |  |  |  |
| 1 | 1 | 580218 | 17.6454 | -0.3817 | 0.6538 |  | 1 | 8 | 27495394 | 16.6544 | 0.3694 | -0.3694 | 1.5253 |
| 2 | 1 | 39719661 | 18.5866 | -0.3969 | 0.7871 |  | 2 | 9 | 12289928 | 22.7877 | -0.4437 | 0.4437 | 2.2002 |
| 3 | 2 | 4346020 | 22.4278 | 0.4395 | 0.6522 |  |  |  |  |  |  |  |  |
| 4 | 2 | 20481915 | 39.5567 | 0.6259 | 0.8745 |  |  |  |  |  |  |  |  |
| 5 | 2 | 25079124 | 44.8750 | -0.6854 | 1.1487 |  |  |  |  |  |  |  |  |
| 6 | 2 | 35005427 | 10.9096 | 0.2954 | 0.7791 |  |  |  |  |  |  |  |  |
| 7 | 3 | 35145112 | 11.0291 | 0.2844 | 0.9100 |  |  |  |  |  |  |  |  |
| 8 | 3 | 35437797 | 11.1080 | -0.2955 | 0.4293 |  |  |  |  |  |  |  |  |
| 9 | 4 | 31490102 | 46.0059 | -0.6883 | 1.2483 |  |  |  |  |  |  |  |  |
| 10 | 4 | 33294138 | 24.7448 | -0.4658 | 1.2066 |  |  |  |  |  |  |  |  |
| 11 | 5 | 248172 | 14.5578 | 0.3411 | 0.7463 |  |  |  |  |  |  |  |  |
| 12 | 5 | 630924 | 11.0921 | 0.2961 | 0.6193 |  |  |  |  |  |  |  |  |
| 13 | 5 | 5356835 | 31.7745 | -0.5307 | 3.1917 |  |  |  |  |  |  |  |  |
| 14 | 6 | 3189214 | 64.3369 | -0.8887 | 4.1357 |  |  |  |  |  |  |  |  |
| 15 | 6 | 5384404 | 11.0899 | -0.2962 | 0.9578 |  |  |  |  |  |  |  |  |
| 16 | 6 | 8254727 | 18.2192 | -0.3824 | 0.4816 |  |  |  |  |  |  |  |  |
| 17 | 6 | 8508510 | 11.9027 | 0.2504 | 1.0449 |  |  |  |  |  |  |  |  |
| 18 | 6 | 24706996 | 56.4054 | 0.8097 | 2.1902 |  |  |  |  |  |  |  |  |
| 19 | 7 | 8364433 | 50.7121 | -0.7412 | 1.1877 |  |  |  |  |  |  |  |  |
| 20 | 7 | 18236933 | 10.6413 | -0.0890 | 0.9255 |  |  |  |  |  |  |  |  |
| 21 | 8 | 17687290 | 25.5001 | -0.4803 | 2.5604 |  |  |  |  |  |  |  |  |
| 22 | 10 | 9402611 | 17.1034 | 0.2964 | 1.5402 |  |  |  |  |  |  |  |  |
| 23 | 11 | 2678930 | 26.9119 | -0.4883 | 1.1722 |  |  |  |  |  |  |  |  |
| 24 | 11 | 3505182 | 54.131 | 0.7765 | 2.1896 |  |  | | | | | |  |
| 25 | 11 | 14219838 | 20.5876 | 0.4083 | 1.9419 |  |  |  |  |  |  |  |  |
| 26 | 12 | 424245 | 16.6283 | -0.3623 | 1.5308 |  |  |  |  |  |  |  |  |
| 27 | 12 | 10698465 | 24.6932 | -0.4728 | 1.7272 |  |  |  |  |  |  |  |  |
| ***Suggested QTNs with the LOD score ≥ 3.0 but the P-value > 0.05/m, where m is the number of markers*** | | | | | |  |  |  |  |  |  |  |  |
|  |  |  |  |  |  |  |  |  |  |  |  |  |  |
| 1 | 2 | 33674994 | 5.1423 | 0.1973 | 0.3179 |  |  |  |  |  |  |  |  |
| 2 | 5 | 22190487 | 7.2697 | -0.2315 | 0.5057 |  |  |  |  |  |  |  |  |
| 3 | 7 | 1146652 | 5.6663 | -0.2082 | 0.3617 |  |  |  |  |  |  |  |  |

**Table S18 Main-effect QTNs and QTN-by-environment interactions for grain width in ratoon rice of 159 rice accessions in two environments using multi-environment joint analysis**

| Main effect QTNs for grain width in ratoon rice | | | | | |  | QTN-by-environment interactions for grain width in ratoon rice | | | | | | |
| --- | --- | --- | --- | --- | --- | --- | --- | --- | --- | --- | --- | --- | --- |
| ID | Chr | Position (bp) | LOD (Q) | add | r^2^(%) |  | ID | Chr | Position (bp) | LOD (QE) | add*env1 | add*env2 | r^2^(%) |
| ***Significant QTNs at the critical P-value of 2.48e-08 (=0.05/m, where m is the number of markers)*** | | | | | |  | ***Significant QTNs at the critical P-value of 2.48e-08 (=0.05/m, where m is the number of markers)*** | | | | | | |
|  |  |  |  |  |  |  |  |  |  |  |  |  |  |
| 1 | 2 | 4438556 | 12.7337 | 0.0250 | 0.6968 |  | 1 | 2 | 7733574 | 12.9165 | -0.0246 | 0.0246 | 0.7436 |
| 2 | 2 | 25565194 | 34.7859 | -0.0437 | 0.5675 |  | 2 | 10 | 710130 | 7.6514 | -0.0186 | 0.0186 | 0.4236 |
| 3 | 2 | 29413021 | 11.492 | 0.0226 | 0.4634 |  |  |  |  |  |  |  |  |
| 4 | 3 | 3390977 | 9.5865 | 0.0205 | 0.1967 |  |  |  |  |  |  |  |  |
| 5 | 3 | 14636351 | 11.6905 | 0.0203 | 0.4402 |  |  |  |  |  |  |  |  |
| 6 | 3 | 16504015 | 14.2389 | -0.0259 | 0.7082 |  |  |  |  |  |  |  |  |
| 7 | 3 | 22162176 | 11.9409 | -0.0232 | 0.3209 |  |  |  |  |  |  |  |  |
| 8 | 3 | 27973483 | 31.607 | -0.0419 | 0.3608 |  |  |  |  |  |  |  |  |
| 9 | 4 | 4722231 | 18.5414 | -0.0295 | 0.4880 |  |  |  |  |  |  |  |  |
| 10 | 4 | 13491272 | 15.2251 | 0.0264 | 0.7100 |  |  |  |  |  |  |  |  |
| 11 | 5 | 5358236 | 55.8300 | -0.0593 | 3.7483 |  |  |  |  |  |  |  |  |
| 12 | 5 | 13900081 | 12.3287 | 0.0235 | 0.6710 |  |  |  |  |  |  |  |  |
| 13 | 5 | 21159341 | 25.3342 | -0.0358 | 0.6647 |  |  |  |  |  |  |  |  |
| 14 | 6 | 14040627 | 20.1295 | 0.0318 | 0.4307 |  |  |  |  |  |  |  |  |
| 15 | 6 | 23191777 | 58.1151 | 0.0639 | 0.9187 |  |  |  |  |  |  |  |  |
| 16 | 7 | 25284287 | 62.4030 | 0.0657 | 1.3675 |  |  |  |  |  |  |  |  |
| 17 | 8 | 487221 | 43.3514 | -0.0498 | 0.8752 |  |  |  |  |  |  |  |  |
| 18 | 8 | 6166430 | 23.4842 | 0.0339 | 1.0234 |  |  |  |  |  |  |  |  |
| 19 | 8 | 8915046 | 11.5364 | -0.0228 | 0.5720 |  |  |  |  |  |  |  |  |
| 20 | 8 | 12442028 | 33.9802 | 0.0438 | 0.5111 |  |  |  |  |  |  |  |  |
| 21 | 9 | 7342896 | 39.5741 | 0.0470 | 1.2041 |  |  |  |  |  |  |  |  |
| 22 | 10 | 4958252 | 17.7834 | -0.0294 | 0.8852 |  |  |  |  |  |  |  |  |
| 23 | 10 | 11172977 | 9.2935 | 0.0202 | 0.3986 |  |  |  |  |  |  |  |  |
| 24 | 11 | 7048922 | 8.2464 | -0.0189 | 0.1498 |  |  | | | | | |  |
| 25 | 11 | 7848256 | 9.1518 | -0.0191 | 0.4086 |  |  |  |  |  |  |  |  |
| 26 | 11 | 16768397 | 53.5398 | -0.0575 | 1.1317 |  |  |  |  |  |  |  |  |
| 27 | 11 | 25125749 | 47.4877 | -0.0528 | 2.0390 |  |  |  |  |  |  |  |  |
| 28 | 12 | 4584004 | 13.0056 | 0.0184 | 0.7209 |  |  |  |  |  |  |  |  |
| 29 | 12 | 10711089 | 22.6157 | 0.0335 | 1.1879 |  |  |  |  |  |  |  |  |
| ***Suggested QTNs with the LOD score ≥ 3.0 but the P-value > 0.05/m, where m is the number of markers*** | | | | | |  |  |  |  |  |  |  |  |
|  |  |  |  |  |  |  |  |  |  |  |  |  |  |
| 1 | 3 | 31973046 | 5.7008 | -0.0150 | 0.3026 |  |  |  |  |  |  |  |  |
| 2 | 3 | 35262383 | 5.1097 | 0.0150 | 0.2710 |  |  |  |  |  |  |  |  |
| 3 | 11 | 26058071 | 4.0679 | 0.0132 | 0.2124 |  |  |  |  |  |  |  |  |

**Table S19 Main-effect QTNs and QTN-by-environment interactions for grain length in ratoon rice of 159 rice accessions in two environments using multi-environment joint analysis**

| Main effect QTNs for grain length in ratoon rice | | | | | |  | QTN-by-environment interactions for grain length in ratoon rice | | | | | | |
| --- | --- | --- | --- | --- | --- | --- | --- | --- | --- | --- | --- | --- | --- |
| No. | Chr | Position (bp) | LOD (Q) | add | r^2^(%) |  | No. | Chr | Position (bp) | LOD (QE) | add*env1 | add*env2 | r^2^(%) |
| ***Significant QTNs at the critical P-value of 2.48e-08 (=0.05/m, where m is the number of markers)*** | | | | | |  | ***Significant QTNs at the critical P-value of 2.48e-08 (=0.05/m, where m is the number of markers)*** | | | | | | |
|  |  |  |  |  |  |  |  |  |  |  |  |  |  |
| 1 | 1 | 25212877 | 15.9230 | -0.0692 | 0.2557 |  | 1 | 4 | 20228091 | 15.7721 | -0.0688 | 0.0688 | 0.7362 |
| 2 | 2 | 5151266 | 11.0671 | 0.0564 | 0.4954 |  | 2 | 6 | 26752211 | 21.2258 | 0.0806 | -0.0806 | 1.0259 |
| 3 | 2 | 17474355 | 9.6785 | 0.0527 | 0.2148 |  |  |  |  |  |  |  |  |
| 4 | 2 | 20073320 | 11.8733 | -0.0614 | 0.4711 |  |  |  |  |  |  |  |  |
| 5 | 3 | 16708508 | 57.6390 | 0.1546 | 3.5766 |  |  |  |  |  |  |  |  |
| 6 | 3 | 16845802 | 36.5658 | 0.1146 | 2.0171 |  |  |  |  |  |  |  |  |
| 7 | 3 | 34087282 | 39.4380 | 0.1188 | 0.4197 |  |  |  |  |  |  |  |  |
| 8 | 4 | 3855798 | 74.6811 | -0.1890 | 1.3100 |  |  |  |  |  |  |  |  |
| 9 | 4 | 4591488 | 18.7990 | 0.0757 | 0.3924 |  |  |  |  |  |  |  |  |
| 10 | 4 | 9493312 | 27.9829 | -0.0964 | 0.4843 |  |  |  |  |  |  |  |  |
| 11 | 4 | 35129844 | 23.6844 | -0.0869 | 0.4740 |  |  |  |  |  |  |  |  |
| 12 | 5 | 5914985 | 45.9299 | 0.1320 | 0.9316 |  |  |  |  |  |  |  |  |
| 13 | 5 | 14607988 | 12.1241 | -0.0597 | 0.1565 |  |  |  |  |  |  |  |  |
| 14 | 5 | 20889199 | 26.9320 | 0.0959 | 0.3029 |  |  |  |  |  |  |  |  |
| 15 | 6 | 6533624 | 21.3076 | 0.0827 | 0.6676 |  |  |  |  |  |  |  |  |
| 16 | 7 | 24964429 | 67.6761 | -0.1752 | 1.4424 |  |  |  |  |  |  |  |  |
| 17 | 7 | 28370748 | 24.2758 | -0.0879 | 0.3858 |  |  |  |  |  |  |  |  |
| 18 | 8 | 3954657 | 11.8470 | -0.0588 | 0.4127 |  |  |  |  |  |  |  |  |
| 19 | 8 | 20516196 | 19.3989 | -0.0803 | 0.8544 |  |  |  |  |  |  |  |  |
| 20 | 9 | 16419362 | 39.1286 | 0.1186 | 0.7065 |  |  |  |  |  |  |  |  |
| 21 | 10 | 14508748 | 9.5444 | -0.0521 | 0.3824 |  |  |  |  |  |  |  |  |
| 22 | 11 | 8501237 | 16.7441 | -0.0761 | 0.4312 |  |  |  |  |  |  |  |  |
| 23 | 11 | 17830860 | 25.8279 | 0.0912 | 1.2305 |  |  |  |  |  |  |  |  |
| 24 | 11 | 18450177 | 25.6913 | -0.0912 | 1.1420 |  |  | | | | | |  |
| 25 | 11 | 22928269 | 27.9500 | 0.0956 | 1.0533 |  |  |  |  |  |  |  |  |
| 26 | 12 | 43637 | 14.5081 | 0.0661 | 0.2501 |  |  |  |  |  |  |  |  |
| 27 | 12 | 4438901 | 12.6198 | -0.0606 | 0.4930 |  |  |  |  |  |  |  |  |
| 28 | 12 | 13963973 | 8.8320 | -0.050 | 0.3390 |  |  |  |  |  |  |  |  |
| 29 | 12 | 22702465 | 10.6073 | -0.0571 | 0.4154 |  |  |  |  |  |  |  |  |
| ***Suggested QTNs with the LOD score ≥ 3.0 but the P-value > 0.05/m, where m is the number of markers*** | | | | | |  |  |  |  |  |  |  |  |
|  |  |  |  |  |  |  |  |  |  |  |  |  |  |
| 1 | 6 | 6777283 | 4.2638 | 0.0342 | 0.1614 |  |  |  |  |  |  |  |  |
| 2 | 6 | 20496138 | 6.9149 | -0.0437 | 0.2632 |  |  |  |  |  |  |  |  |

**Table S20 Main-effect QTNs and QTN-by-environment interactions for thousand grain weight in ratoon rice of 159 rice accessions in two environments using multi-environment joint analysis**

| Main effect QTNs for thousand grain weight in ratoon rice | | | | | |  | QTN-by-environment interactions for thousand grain weight in ratoon rice | | | | | | |
| --- | --- | --- | --- | --- | --- | --- | --- | --- | --- | --- | --- | --- | --- |
| ID | Chr | Position (bp) | LOD (Q) | add | r^2^(%) |  | ID | Chr | Position (bp) | LOD (QE) | add*env1 | add*env2 | r^2^(%) |
| ***Significant QTNs at the critical P-value of 2.48e-08 (=0.05/m, where m is the number of markers)*** | | | | | |  | ***Suggested QTNs with the LOD score ≥ 3.0 but the P-value > 0.05/m, where m is the number of markers*** | | | | | | |
|  |  |  |  |  |  |  |  |  |  |  |  |  |  |
| 1 | 1 | 4921429 | 8.6017 | 0.1184 | 1.9646 |  | 1 | 7 | 15307889 | 4.5104 | -0.3033 | 0.3033 | 1.0407 |
| 2 | 3 | 22986087 | 19.7952 | -0.6664 | 2.0165 |  |  |  |  |  |  |  |  |
| 3 | 4 | 4570606 | 16.6756 | -0.6044 | 1.5781 |  |  |  |  |  |  |  |  |
| 4 | 4 | 24390487 | 21.4844 | 0.6987 | 1.6579 |  |  |  |  |  |  |  |  |
| 5 | 4 | 27688228 | 9.0793 | -0.4360 | 2.1372 |  |  |  |  |  |  |  |  |
| 6 | 4 | 31939665 | 53.4528 | -1.2542 | 5.3728 |  |  |  |  |  |  |  |  |
| 7 | 5 | 14238870 | 15.5683 | 0.5738 | 1.4108 |  |  |  |  |  |  |  |  |
| 8 | 5 | 14579088 | 32.3958 | -0.9063 | 3.0528 |  |  |  |  |  |  |  |  |
| 9 | 5 | 19116277 | 7.9108 | 0.2922 | 1.4597 |  |  |  |  |  |  |  |  |
| 10 | 6 | 12843781 | 11.0815 | 0.4841 | 0.7439 |  |  |  |  |  |  |  |  |
| 11 | 6 | 13123202 | 8.3084 | -0.4162 | 0.5950 |  |  |  |  |  |  |  |  |
| 12 | 6 | 14099638 | 9.5350 | -0.4613 | 1.3231 |  |  |  |  |  |  |  |  |
| 13 | 8 | 8333745 | 10.6489 | -0.4765 | 1.5629 |  |  |  |  |  |  |  |  |
| 14 | 8 | 16572646 | 19.1127 | -0.6743 | 1.2154 |  |  |  |  |  |  |  |  |
| 15 | 9 | 5251526 | 7.3255 | 0.3944 | 1.5320 |  |  |  |  |  |  |  |  |
| 16 | 11 | 3518389 | 8.8094 | -0.4266 | 1.2922 |  |  |  |  |  |  |  |  |
| 17 | 11 | 14219838 | 13.6805 | 0.5068 | 3.3536 |  |  |  |  |  |  |  |  |
| 18 | 11 | 17131649 | 33.0474 | 0.9235 | 3.0028 |  |  |  |  |  |  |  |  |
| 19 | 11 | 18828665 | 10.1167 | -0.4594 | 1.4236 |  |  |  |  |  |  |  |  |
| 20 | 12 | 10561772 | 8.4207 | -0.4178 | 1.3049 |  |  |  |  |  |  |  |  |
| ***Suggested QTNs with the LOD score ≥ 3.0 but the P-value > 0.05/m, where m is the number of markers*** | | | | | |  |  |  |  |  |  |  |  |
|  |  |  |  |  |  |  |  |  |  |  |  |  |  |
| 1 | 8 | 17696038 | 4.3115 | 0.3002 | 1.0194 |  |  |  |  |  |  |  |  |
| 2 | 12 | 2900755 | 4.0200 | 0.2841 | 0.9072 |  |  |  |  |  |  |  |  |

**Table S21 Candidate genes around main-effect QTNs of rice grain size in main crop**

| **Trait** | **No.** | **Locus** | |  | **LOD scores** | | | **r^2^ (%)** |  | **Gene differential expression analysis** | | |  | | **GO annotation** | | | | |
| --- | --- | --- | --- | --- | --- | --- | --- | --- | --- | --- | --- | --- | --- | --- | --- | --- | --- | --- | --- |
|  |  | **Chr** | **Posi (bp)** |  | **I** | **II** | **III** |  |  | **Gene_ID** | **log2(Fold Change)** | **P-value** | |  | **GO_ID** | **GO_name** | **E-value** | | **Reference** |
| Grain width | 1 | 2 | 1541597 |  |  |  | 26.93 | 0.80 |  | Os02g0126400 | 1.03 | 2.43E-02 | |  | GO:0046777 | protein autophosphorylation | | 0 | Qiu et al., 2016 |
|  | 2 | 2 | 4944049 |  | 9.72 |  |  | 1.44 |  | Os02g0187800 | -1.02 | 9.04E-03 | |  | GO:0009809 | lignin biosynthetic process | | 0 | Zhang et al., 2021 |
|  | 3 | 2 | 19799308 |  |  |  | 6.68 | 0.21 |  | Os02g0538000 | 1.23 | 6.74E-03 | |  | GO:0009793 | embryo development ending in seed dormancy | | 0 | Figueiredo et al., 2014 |
|  | 4 | 3 | 16819050 |  |  |  | 11.37 | 0.52 |  | Os03g0411500 | -1.22 | 4.09E-03 | |  | GO:0015979 | photosynthesis | | 2.50E-107 | Chen et al., 2021 |
|  | 5 | 3 | 24648339 |  |  | 9.14 | 25.56 | 1.30~2.28 |  | Os03g0640100 | 1.06 | 1.31E-02 | |  | GO:0045787 | positive regulation of cell cycle | | 0 | Guo et al., 2022 |
|  | 6 | 3 | 28864309 |  | 16.09 |  |  | 2.09 |  | Os03g0717700 | 1.12 | 3.00E-03 | |  | GO:0006468 | protein phosphorylation | | 0 | Qiu et al., 2016 |
|  | 7 | 7 | 3682234 |  |  | 11.35 |  | 0.84 |  | Os07g0166800 | 1.51 | 1.95E-02 | |  | GO:0006511 | ubiquitin-dependent protein catabolic process | | 1.88E-109 | Li et al., 2018 |
|  | 8 | 8 | 6060988 |  |  |  | 11.03 | 0.49 |  | Os08g0205900 | -1.02 | 2.43E-02 | |  | GO:0005985 | sucrose metabolic process | | 0 | Nakamura et al., 1992 |
|  | 9 | 10 | 22628897 |  |  |  | 18.64 | 0.58 |  | Os10g0567400 | 1.75 | 4.09E-04 | |  | GO:0015995 | chlorophyll biosynthetic process | | 0 | Chen et al., 2021 |
|  | 10 | 12 | 10350434 |  |  |  | 17.13 | 0.73 |  | Os12g0277500 | -1.19 | 3.71E-03 | |  | GO:0009793 | embryo development ending in seed dormancy | | 0 | Figueiredo et al., 2014 |
| Grain length | 1 | 1 | 9828171 |  |  | 25.54 |  | 0.96 |  | Os01g0280500 | -1.01 | 2.37E-02 | |  | GO:0009793 | embryo development ending in seed dormancy | | 8.15E-143 | Figueiredo et al., 2014 |
|  | 2 | 2 | 5436687 |  |  |  | 7.29 | 0.18 |  | Os02g0197600 | -1.33 | 3.03E-02 | |  | GO:0009735 | response to cytokinin | | 4.31E-147 | Jameson et al., 2016 |
|  | 3 | 2 | 24585913 |  |  |  | 48.81 | 0.41 |  | Os02g0621300 | -1.17 | 1.05E-02 | |  | GO:0009737 | response to abscisic acid | | 0 | Qin et al., 2021 |
|  | 4 | 2 | 24992114 |  |  | 13.56 |  | 0.75 |  | Os02g0626100 | -1.27 | 9.33E-03 | |  | GO:0009739 | response to gibberellin | | 0 | Shi et al., 2020 |
|  | 5 | 3 | 616193 |  | 37.04 |  | 22.48 | 0.24~2.57 |  | Os03g0108600 | 1.24 | 1.41E-02 | |  | GO:0071369 | cellular response to ethylene stimulus | | 0 | Ma et al., 2018 |
|  | 6 | 4 | 4591488 |  |  |  | 49.91 | 0.97 |  | Os04g0169100 | -1.20 | 2.76E-03 | |  | GO:2000904 | regulation of starch metabolic process | | 0 | Zhang et al., 2016 |
|  | 7 | 12 | 776662 |  | 5.44 |  |  | 0.71 |  | Os12g0112500 | -1.15 | 2.37E-02 | |  | GO:0005983 | starch catabolic process | | 8.5E-132 | Zhang et al., 2016 |
| Thousand grain weight | 1 | 5 | 22017452 |  |  | 10.27 |  | 1.82 |  | Os05g0445900 | 1.19 | 1.48E-02 | |  | GO:0080111 | DNA demethylation | | 0 | Zhu et al., 2018 |

**Reference:**

1. Chen, K., Łyskowski, A., Jaremko, Ł., and Jaremko, M. (2021). Genetic and molecular factors determining grain weight in rice. *Front. Plant Sci*. 12, 605799. doi: 10.3389/fpls.2021.605799
2. Figueiredo, D. D., and Köhler, C. (2014). Signalling events regulating seed coat development. *Biochem. Soc. Trans*. 42, 358–363. doi: 10.1042/BST20130221
3. Guo, M., Zhang, W., Mohammadi, M. A., He, Z., She, Z., Yan, M., et al. (2022). *OsDDM1b* controls grain size by influencing cell cycling and regulating homeostasis and signaling of brassinosteroid in rice. *Front. Plant Sci*. 13, 873993. doi: 10.3389/fpls.2022.873993
4. Jameson, P. E., and Song, J. (2016). Cytokinin: a key driver of seed yield. *J. Exp. Bot*. 67, 593–606. [doi](https://doi): 10.1093/jxb/erv461
5. Li, N., Xu, R., Duan, P., and Li, Y. (2018). Control of grain size in rice. *Plant Reprod*. 31, 237–251. doi: 10.1007/s00497-018-0333-6
6. Liu, J., Wu, M. W., and Liu, C. M. (2022). Cereal endosperms: development and storage product accumulation. *Ann. Rev. Plant Biol*. 73, 255–291. [doi](https://doi): 10.1146/annurev-arplant-070221-024405
7. Ma, B., Zhou, Y., Chen, H., He, S. J., Huang, Y. H., Zhao, H., et al. (2018). Membrane protein MHZ3 stabilizes *OsEIN2* in rice by interacting with its Nramp-like domain. *Proc. Natl. Acad. Sci. U. S. A*. 115, 2520–2525. [doi](https://doi): 10.1073/pnas.1718377115
8. Nakamura, Y., and Yuki, K. (1992). Changes in enzyme activities associated with carbohydrate metabolism during the development of rice endosperm. *Plant Sci*. 82, 15-20. doi: 10.1016/0168-9452(92)90003-5
9. Qin, P., Zhang, G., Hu, B., Wu, J., Chen, W., Ren, Z., et al. (2021). Leaf-derived ABA regulates rice seed development via a transporter-mediated and temperature-sensitive mechanism. *Sci. Adv*. 7, eabc8873. [doi](https://doi): 10.1126/sciadv.abc8873
10. Qiu, J., Hou, Y., Tong, X., Wang, Y., Lin, H., Liu, Q., et al. (2016). Quantitative phosphoproteomic analysis of early seed development in rice (*Oryza sativa* L.). *Plant Mol. Biol*. 90, 249–265. doi: 10.1007/s11103-015-0410-2
11. Shi, C. L., Dong, N. Q., Guo, T., Ye, W. W., Shan, J. X., and Lin, H. X. (2020). A quantitative trait locus *GW6* controls rice grain size and yield through the gibberellin pathway. *Plant J. Cell Mol. Biol*. 103, 1174–1188. [doi](https://doi): 10.1111/tpj.14793
12. Zhang, H., Lu, Y., Zhao, Y., and Zhou, D. X. (2016). *OsSRT1* is involved in rice seed development through regulation of starch metabolism gene expression. *Plant Sci.* 248, 28–36. doi: 10.1016/j.plantsci.2016.04.004
13. Zhang, Y. M., Yu, H. X., Ye, W. W., Shan, J. X., Dong, N. Q., Guo, T., et al. (2021). A rice QTL *GS3.1* regulates grain size through metabolic-flux distribution between flavonoid and lignin metabolons without affecting stress tolerance. *Commun. Biol*. 4, 1171. [doi: 10.1038/s42003-021-02686-x](https://doi.org/10.1038/s42003-021-02686-x)
14. Zhu, H., Xie, W., Xu, D., Miki, D., Tang, K., Huang, C. F., et al. (2018). DNA demethylase ROS1 negatively regulates the imprinting of DOGL4 and seed dormancy in Arabidopsis thaliana. *Proc. Natl. Acad. Sci. U. S. A*. 115, E9962–E9970. [doi](https://doi): 10.1073/pnas.1812847115

**Table S22 Candidate genes around main-effect QTNs of rice grain size in ratoon rice**

| **Trait** | **Locus** | |  | **LOD scores** | | | | | | **r^2^ (%)** |  | **Gene differential expression analysis** | | |  | **GO annotation** | | | |
| --- | --- | --- | --- | --- | --- | --- | --- | --- | --- | --- | --- | --- | --- | --- | --- | --- | --- | --- | --- |
|  | **Chr** | **Posi (bp)** |  | **I** | | | **II** | | **III** |  |  | **Gene_ID** | **log2(Fold Change)** | **P-value** |  | **GO_ID** | **GO_name** | **E-value** | **Reference** |
| Grain width | 2 | 4438556 |  |  | |  | | 12.73 | | 0.70 |  | Os02g0178800 | 1.36 | 1.76E-03 |  | GO:0009737 | response to abscisic acid | 0 | Qin et al., 2021 |
|  | 2 | 4994560 |  | 8.53 |  | | |  | | 1.37 |  | Os02g0187800 | -1.02 | 9.04E-03 |  | GO:0009809 | lignin biosynthetic process | 0 | Zhang et al., 2021 |
|  | 2 | 5481928 |  | 16.42 |  | | |  | | 2.46 |  | Os02g0197600 | -1.33 | 3.03E-02 |  | GO:0009735 | response to cytokinin | 4.31E-147 | Jameson et al., 2016 |
|  | 3 | 22162176 |  |  |  | | | 11.94 | | 0.32 |  | Os03g0592500 | -1.59 | 8.37E-04 |  | GO:0071215 | cellular response to abscisic acid stimulus | 1.10E-150 | Qin et al., 2021 |
|  | 3 | 27973483 |  | 22.72 |  | | | 31.61 | | 0.36~0.62 |  | Os03g0695700 | 1.13 | 5.82E-03 |  | GO:0009826 | unidimensional cell growth | 0 | Huang et al., 2017 |
|  | 4 | 4722231 |  |  | 24.41 | | | 18.54 | | 0.49~2.30 |  | Os04g0169100 | -1.20 | 2.76E-03 |  | GO:2000904 | regulation of starch metabolic process | 0 | Zhang et al., 2016 |
|  | 8 | 5984678~6166430 |  |  | 17.35 | | | 23.48 | | 1.02~2.29 |  | Os08g0205900 | -1.02 | 2.43E-02 |  | GO:0005985 | sucrose metabolic process | 0 | Nakamura et al., 1992 |
|  | 11 | 7818816~7848256 |  | 8.11 |  | | | 9.15 | | 0.41~1.01 |  | Os11g0242800 | -1.29 | 4.95E-03 |  | GO:0015979 | photosynthesis | 1.17E-147 | Chen et al., 2021 |
| Grain length | 2 | 20073320 |  |  |  | | | 11.87 | | 0.47 |  | Os02g0538000 | 1.23 | 6.74E-03 |  | GO:0009793 | embryo development ending in seed dormancy | 0 | Figueiredo et al., 2014 |
|  | 4 | 4591488 |  |  |  | | | 18.80 | | 0.39 |  | Os04g0169100 | -1.20 | 2.76E-03 |  | GO:2000904 | regulation of starch metabolic process | 0 | Zhang et al., 2016 |
|  | 4 | 25746858 |  | 15.38 |  | | |  | | 3.53 |  | Os04g0514800 | -1.02 | 5.96E-03 |  | GO:0009850 | auxin metabolic process | 0 | Liu et al., 2015 |
|  | 10 | 14508748 |  |  |  | | | 9.54 | | 0.38 |  | Os10g0418000 | -1.34 | 4.79E-02 |  | GO:0044030 | regulation of DNA methylation | 6.13E-78 | Xing et al., 2015 |
| Thousand grain weight | 2 | 24702111 |  | 10.65 |  | | |  | | 1.89 |  | Os02g0621300 | -1.17 | 1.05E-02 |  | GO:0009737 | response to abscisic acid | 0 | Qin et al., 2021 |
|  | 3 | 16806894 |  |  | 4.93 | | |  | | 1.27 |  | Os03g0411500 | -1.22 | 4.09E-03 |  | GO:0015979 | photosynthesis | 2.50E-107 | Chen et al., 2021 |
|  | 3 | 22986087 |  |  |  | | | 19.80 | | 2.02 |  | Os03g0607400 | -1.33 | 1.51E-02 |  | GO:0051512 | positive regulation of unidimensional cell growth | 1.02E-81 | Si et al., 2016 |

Reference:

1. Chen, K., Łyskowski, A., Jaremko, Ł., and Jaremko, M. (2021). Genetic and molecular factors determining grain weight in rice. *Front. Plant Sci.* 12, 605799. doi: 10.3389/fpls.2021.605799
2. Figueiredo, D. D., and Köhler, C. (2014). Signalling events regulating seed coat development. *Biochem. Soc. Trans*. 42, 358–363. doi: 10.1042/BST20130221
3. Huang, K., Wang, D., Duan, P., Zhang, B., Xu, R., Li, N., et al. (2017). Wide and thick grain 1, which encodes an otubain-like protease with deubiquitination activity, influences grain size and shape in rice. *Plant J*. 91, 849–860. doi: 10.1111/tpj.13613
4. Jameson, P. E., and Song, J. (2016). Cytokinin: a key driver of seed yield. *J. Exp. Bot.* 67, 593–606. doi: 10.1093/jxb/erv461
5. Liu, L., Tong, H., Xiao, Y., Che, R., Xu, F., Hu, B., et al. (2015). Activation of big grain1 significantly improves grain size by regulating auxin transport in rice. *Proc. Natl. Acad. Sci. U. S. A*. 112, 11102–11107. doi: 10.1073/pnas.1512748112
6. Qin, P., Zhang, G., Hu, B., Wu, J., Chen, W., Ren, Z., et al. (2021). Leaf-derived ABA regulates rice seed development via a transporter-mediated and temperature-sensitive mechanism. *Sci. Adv*. 7, eabc8873. [doi](https://doi): 10.1126/sciadv.abc8873
7. Si, L., Chen, J., Huang, X., Gong, H., Luo, J., Hou, Q., et al. (2016). *OsSPL13* controls grain size in cultivated rice. *Nat. Genet*. 48, 447–456. doi: 10.1038/ng.3518
8. Xing, M. Q., Zhang, Y. J., Zhou, S. R., Hu, W. Y., Wu, X. T., Ye, Y. J., et al. (2015). Global analysis reveals the crucial roles of DNA methylation during rice seed development. *Plant Physiol*. 168 1417–1432. doi: 10.1104/pp.15.00414
9. Zhang, H., Lu, Y., Zhao, Y., and Zhou, D. X. (2016). *OsSRT1* is involved in rice seed development through regulation of starch metabolism gene expression. *Plant Sci*. 248, 28–36. doi: 10.1016/j.plantsci.2016.04.004
10. Zhang, Y. M., Yu, H. X., Ye, W. W., Shan, J. X., Dong, N. Q., Guo, T., et al. (2021). A rice QTL *GS3.1* regulates grain size through metabolic-flux distribution between flavonoid and lignin metabolons without affecting stress tolerance. *Commun. Biol*. 4, 1171. doi: 10.1038/s42003-021-02686-x

**Table S23 Main-effect QTN for grain width, grain length, and thousand grain weight in main crop and ratoon rice of 159 rice accessions in single environment and multi-environment joint analysis using evolutionary population**

| **No.** | **Trait** | **MC or RR** | **Environment** | **Marker** | **Chromosome** | **Position (bp)** | **LOD** | **add** | **variance** | **r2(%)** | **P-value** | **significance** |
| --- | --- | --- | --- | --- | --- | --- | --- | --- | --- | --- | --- | --- |
| 1 | GW | MC | 1 | 140490468 | 1 | 40490468 | 4.3648 | 0.0331 | 0.0011 | 1.2262 | 4.3183E-05 | SUG |
| 2 | GW | MC | 1 | 201050680 | 2 | 1050680 | 14.0231 | 0.0658 | 0.0015 | 1.7669 | 9.28686E-16 | SIG |
| 3 | GW | MC | 1 | 326622478 | 3 | 26622478 | 18.7354 | -0.0818 | 0.0052 | 5.9084 | 1.56446E-20 | SIG |
| 4 | GW | MC | 1 | 421031164 | 4 | 21031164 | 14.4951 | -0.0673 | 0.0017 | 1.9938 | 3.08196E-16 | SIG |
| 5 | GW | MC | 1 | 431159467 | 4 | 31159467 | 20.8742 | 0.087 | 0.0033 | 3.8071 | 1.07827E-22 | SIG |
| 6 | GW | MC | 1 | 505361276 | 5 | 5361276 | 20.2645 | -0.0859 | 0.0051 | 5.7792 | 4.45336E-22 | SIG |
| 7 | GW | MC | 1 | 513788807 | 5 | 13788807 | 3.9669 | 0.0313 | 0.001 | 1.1192 | 1.91947E-05 | SUG |
| 8 | GW | MC | 1 | 810329229 | 8 | 10329229 | 14.5155 | 0.0696 | 0.0037 | 4.2501 | 2.93915E-16 | SIG |
| 9 | GW | MC | 1 | 816385094 | 8 | 16385094 | 16.6274 | -0.0752 | 0.0023 | 2.5796 | 2.36178E-17 | SIG |
| 10 | GW | MC | 1 | 826714889 | 8 | 26714889 | 12.7967 | -0.062 | 0.0009 | 1.0379 | 1.63464E-14 | SIG |
| 11 | GW | MC | 1 | 1101582356 | 11 | 1582356 | 11.199 | -0.0575 | 0.0022 | 2.5204 | 6.32976E-12 | SIG |
| 12 | GW | MC | 1 | 1126376406 | 11 | 26376406 | 10.8635 | 0.0559 | 0.0031 | 3.5555 | 1.51697E-12 | SIG |
| 13 | GW | MC | 2 | 134815173 | 1 | 34815173 | 10.7976 | -0.0387 | 0.0005 | 0.4983 | 1.77058E-12 | SIG |
| 14 | GW | MC | 2 | 140476043 | 1 | 40476043 | 4.4284 | 0.0207 | 0.0005 | 0.5853 | 3.73079E-05 | SUG |
| 15 | GW | MC | 2 | 204878888 | 2 | 4878888 | 19.3927 | -0.057 | 0.0016 | 1.7731 | 3.38666E-21 | SIG |
| 16 | GW | MC | 2 | 220138937 | 2 | 20138937 | 11.3542 | 0.0398 | 0.0016 | 1.738 | 4.79745E-13 | SIG |
| 17 | GW | MC | 2 | 314483625 | 3 | 14483625 | 13.4957 | 0.0444 | 0.0018 | 1.9653 | 3.18671E-15 | SIG |
| 18 | GW | MC | 2 | 321778824 | 3 | 21778824 | 19.4634 | -0.058 | 0.0011 | 1.1759 | 3.44637E-20 | SIG |
| 19 | GW | MC | 2 | 326795140 | 3 | 26795140 | 7.7983 | -0.0323 | 0.001 | 1.0556 | 2.06527E-09 | SIG |
| 20 | GW | MC | 2 | 335144023 | 3 | 35144023 | 10.6693 | 0.0406 | 0.0013 | 1.4206 | 2.14312E-11 | SIG |
| 21 | GW | MC | 2 | 413960907 | 4 | 13960907 | 14.8756 | -0.0478 | 0.0013 | 1.4552 | 1.26738E-16 | SIG |
| 22 | GW | MC | 2 | 504631781 | 5 | 4631781 | 30.3826 | -0.0819 | 0.0044 | 4.9051 | 2.78273E-32 | SIG |
| 23 | GW | MC | 2 | 504775669 | 5 | 4775669 | 15.9931 | -0.0476 | 0.0023 | 2.5094 | 1.01736E-16 | SIG |
| 24 | GW | MC | 2 | 505358771 | 5 | 5358771 | 16.6238 | -0.0513 | 0.0018 | 2.0292 | 2.38143E-17 | SIG |
| 25 | GW | MC | 2 | 906961682 | 9 | 6961682 | 13.3654 | 0.0442 | 0.0008 | 0.8912 | 4.32197E-15 | SIG |
| 26 | GW | MC | 2 | 910486075 | 9 | 10486075 | 8.7021 | 0.0341 | 0.0011 | 1.1834 | 2.44601E-10 | SIG |
| 27 | GW | MC | 2 | 915748352 | 9 | 15748352 | 18.6728 | 0.0595 | 0.0007 | 0.7739 | 2.1278E-19 | SIG |
| 28 | GW | MC | 2 | 1125818848 | 11 | 25818848 | 17.0568 | 0.052 | 0.0027 | 2.9866 | 7.81373E-19 | SIG |
| 29 | GW | MC | 2 | 1211133135 | 12 | 11133135 | 15.0736 | 0.0478 | 0.0018 | 1.9932 | 7.98318E-17 | SIG |
| 30 | GW | MC | MEJA | 201236423 | 2 | 1236423 | 29.019 | 0.0557 | 0.0011 | 1.2459 | 6.57469E-31 | SIG |
| 31 | GW | MC | MEJA | 220136939 | 2 | 20136939 | 12.877 | 0.0339 | 0.0011 | 1.2886 | 1.35474E-14 | SIG |
| 32 | GW | MC | MEJA | 223163721 | 2 | 23163721 | 40.9266 | -0.0715 | 0.0007 | 0.8136 | 6.86951E-43 | SIG |
| 33 | GW | MC | MEJA | 321778824 | 3 | 21778824 | 11.7973 | -0.0272 | 0.0007 | 0.7588 | 1.59646E-12 | SIG |
| 34 | GW | MC | MEJA | 504631781 | 5 | 4631781 | 26.3888 | -0.0525 | 0.0018 | 2.0554 | 2.93991E-28 | SIG |
| 35 | GW | MC | MEJA | 505358771 | 5 | 5358771 | 41.1839 | -0.0712 | 0.0035 | 3.982 | 6.57058E-42 | SIG |
| 36 | GW | MC | MEJA | 506052088 | 5 | 6052088 | 9.3278 | -0.0283 | 0.0008 | 0.8856 | 5.60237E-11 | SIG |
| 37 | GW | MC | MEJA | 514169729 | 5 | 14169729 | 6.1061 | -0.0226 | 0.0005 | 0.5436 | 1.14108E-07 | SUG |
| 38 | GW | MC | MEJA | 806080284 | 8 | 6080284 | 30.8827 | 0.0625 | 0.0008 | 0.8883 | 1.3136E-31 | SIG |
| 39 | GW | MC | MEJA | 810329229 | 8 | 10329229 | 20.5218 | 0.0461 | 0.0016 | 1.84 | 2.44738E-22 | SIG |
| 40 | GW | MC | MEJA | 816963433 | 8 | 16963433 | 25.2891 | -0.0511 | 0.0025 | 2.8006 | 5.1502E-26 | SIG |
| 41 | GW | MC | MEJA | 826714889 | 8 | 26714889 | 42.6112 | -0.073 | 0.0013 | 1.4184 | 1.3924E-44 | SIG |
| 42 | GW | MC | MEJA | 910486075 | 9 | 10486075 | 18.0811 | 0.0417 | 0.0016 | 1.7998 | 7.18211E-20 | SIG |
| 43 | GW | MC | MEJA | 920103851 | 9 | 20103851 | 22.266 | 0.0459 | 0.0008 | 0.9043 | 5.43044E-23 | SIG |
| 44 | GW | MC | MEJA | 1107833619 | 11 | 7833619 | 15.4743 | -0.038 | 0.0011 | 1.2935 | 3.35944E-16 | SIG |
| 45 | GW | MC | MEJA | 1117149260 | 11 | 17149260 | 29.1651 | -0.0589 | 0.0029 | 3.214 | 6.854E-30 | SIG |
| 46 | GW | MC | MEJA | 1125624085 | 11 | 25624085 | 18.3448 | 0.0417 | 0.0017 | 1.9565 | 3.8854E-20 | SIG |
| 47 | GW | MC | MEJA | 1211178871 | 12 | 11178871 | 15.7811 | 0.0394 | 0.0011 | 1.2022 | 1.65756E-16 | SIG |
| 48 | GL | MC | 1 | 301294521 | 3 | 1294521 | 24.4767 | 0.2973 | 0.0236 | 3.3069 | 2.49118E-26 | SIG |
| 49 | GL | MC | 1 | 316708508 | 3 | 16708508 | 33.5357 | 0.3893 | 0.1267 | 17.7195 | 1.86351E-35 | SIG |
| 50 | GL | MC | 1 | 433360398 | 4 | 33360398 | 14.8636 | -0.2078 | 0.0192 | 2.6842 | 1.30355E-16 | SIG |
| 51 | GL | MC | 1 | 525364698 | 5 | 25364698 | 22.0966 | -0.2754 | 0.018 | 2.5176 | 8.0213E-23 | SIG |
| 52 | GL | MC | 1 | 602130119 | 6 | 2130119 | 15.7795 | 0.2164 | 0.0371 | 5.1965 | 1.53683E-17 | SIG |
| 53 | GL | MC | 1 | 604725106 | 6 | 4725106 | 15.7988 | 0.2186 | 0.033 | 4.6198 | 1.46905E-17 | SIG |
| 54 | GL | MC | 1 | 607737965 | 6 | 7737965 | 15.6934 | -0.2146 | 0.0203 | 2.8404 | 1.87849E-17 | SIG |
| 55 | GL | MC | 1 | 714471382 | 7 | 14471382 | 17.2692 | -0.2476 | 0.0114 | 1.601 | 5.38866E-18 | SIG |
| 56 | GL | MC | 1 | 724533051 | 7 | 24533051 | 19.2738 | -0.2502 | 0.0227 | 3.178 | 4.46644E-21 | SIG |
| 57 | GL | MC | 1 | 1117785691 | 11 | 17785691 | 8.2797 | 0.1445 | 0.0194 | 2.7149 | 5.25565E-09 | SIG |
| 58 | GL | MC | 2 | 105419896 | 1 | 5419896 | 5.0602 | -0.0924 | 0.0085 | 1.2475 | 1.38435E-06 | SUG |
| 59 | GL | MC | 2 | 231132137 | 2 | 31132137 | 5.295 | 0.0948 | 0.0081 | 1.1824 | 7.8942E-07 | SUG |
| 60 | GL | MC | 2 | 316708508 | 3 | 16708508 | 35.1278 | 0.347 | 0.1006 | 14.7134 | 4.65926E-37 | SIG |
| 61 | GL | MC | 2 | 431203233 | 4 | 31203233 | 7.8303 | 0.119 | 0.0131 | 1.916 | 1.91467E-09 | SIG |
| 62 | GL | MC | 2 | 525364698 | 5 | 25364698 | 26.9845 | -0.2716 | 0.0181 | 2.6423 | 1.03877E-27 | SIG |
| 63 | GL | MC | 2 | 607759617 | 6 | 7759617 | 5.312 | 0.0954 | 0.0075 | 1.0971 | 7.58033E-07 | SUG |
| 64 | GL | MC | 2 | 702290160 | 7 | 2290160 | 8.5657 | -0.1266 | 0.0143 | 2.0833 | 3.37454E-10 | SIG |
| 65 | GL | MC | 2 | 712384603 | 7 | 12384603 | 17.5341 | -0.1994 | 0.0082 | 1.1971 | 2.92807E-18 | SIG |
| 66 | GL | MC | 2 | 716562571 | 7 | 16562571 | 8.5411 | -0.1128 | 0.0121 | 1.7758 | 2.87904E-09 | SIG |
| 67 | GL | MC | 2 | 818394489 | 8 | 18394489 | 47.0358 | 0.4698 | 0.0232 | 3.3945 | 4.98937E-49 | SIG |
| 68 | GL | MC | 2 | 1204443515 | 12 | 4443515 | 10.4138 | -0.1406 | 0.0184 | 2.6909 | 4.36042E-12 | SIG |
| 69 | GL | MC | 2 | 1222906272 | 12 | 22906272 | 8.9312 | -0.1286 | 0.0158 | 2.3036 | 1.42558E-10 | SIG |
| 70 | GL | MC | 2 | 1224137319 | 12 | 24137319 | 8.9989 | -0.1298 | 0.0162 | 2.3716 | 1.21551E-10 | SIG |
| 71 | GL | MC | MEJA | 100316750 | 1 | 316750 | 20.9872 | 0.1125 | 0.003 | 0.4293 | 8.29056E-23 | SIG |
| 72 | GL | MC | MEJA | 127124415 | 1 | 27124415 | 41.8529 | 0.179 | 0.0096 | 1.3721 | 8.05232E-44 | SIG |
| 73 | GL | MC | MEJA | 217656418 | 2 | 17656418 | 51.4311 | -0.2231 | 0.0206 | 2.9635 | 3.72257E-52 | SIG |
| 74 | GL | MC | MEJA | 224475326 | 2 | 24475326 | 21.0072 | -0.1125 | 0.0038 | 0.542 | 7.91254E-23 | SIG |
| 75 | GL | MC | MEJA | 316708508 | 3 | 16708508 | 88.325 | 0.3488 | 0.1017 | 14.6019 | 1.88142E-90 | SIG |
| 76 | GL | MC | MEJA | 325129163 | 3 | 25129163 | 22.5203 | 0.1155 | 0.013 | 1.8712 | 3.02349E-23 | SIG |
| 77 | GL | MC | MEJA | 334087282 | 3 | 34087282 | 27.2554 | 0.1328 | 0.0036 | 0.5177 | 3.93422E-29 | SIG |
| 78 | GL | MC | MEJA | 401015786 | 4 | 1015786 | 3.9756 | -0.0446 | 0.0009 | 0.1262 | 1.87966E-05 | SUG |
| 79 | GL | MC | MEJA | 520889199 | 5 | 20889199 | 5.9223 | 0.0496 | 0.002 | 0.2807 | 1.19665E-06 | SUG |
| 80 | GL | MC | MEJA | 525364698 | 5 | 25364698 | 44.0519 | -0.187 | 0.0085 | 1.222 | 8.90745E-45 | SIG |
| 81 | GL | MC | MEJA | 602130119 | 6 | 2130119 | 12.6344 | 0.0837 | 0.0056 | 0.7988 | 2.39038E-14 | SIG |
| 82 | GL | MC | MEJA | 617543510 | 6 | 17543510 | 32.878 | -0.1527 | 0.0112 | 1.6037 | 1.32816E-33 | SIG |
| 83 | GL | MC | MEJA | 702288888 | 7 | 2288888 | 15.0264 | -0.0958 | 0.0074 | 1.0676 | 9.42249E-16 | SIG |
| 84 | GL | MC | MEJA | 714471382 | 7 | 14471382 | 35.2393 | -0.1708 | 0.0057 | 0.8196 | 5.78165E-36 | SIG |
| 85 | GL | MC | MEJA | 800471029 | 8 | 471029 | 26.5609 | -0.1305 | 0.0088 | 1.2696 | 1.97169E-28 | SIG |
| 86 | GL | MC | MEJA | 818394489 | 8 | 18394489 | 104.6895 | 0.4245 | 0.019 | 2.7225 | 7.4798E-107 | SIG |
| 87 | GL | MC | MEJA | 1016532434 | 10 | 16532434 | 40.4964 | 0.1747 | 0.0062 | 0.8966 | 1.85939E-42 | SIG |
| 88 | GL | MC | MEJA | 1117785691 | 11 | 17785691 | 3.2574 | 0.0397 | 0.0015 | 0.216 | 0.000553056 | SUG |
| 89 | GL | MC | MEJA | 1211214543 | 12 | 11214543 | 22.9159 | 0.1221 | 0.0119 | 1.7131 | 9.35691E-25 | SIG |
| 90 | GL | MC | MEJA | 1215176154 | 12 | 15176154 | 37.9166 | -0.1665 | 0.0074 | 1.0652 | 7.29864E-40 | SIG |
| 91 | TGW | MC | 1 | 221664294 | 2 | 21664294 | 8.5298 | -0.6283 | 0.2177 | 2.336 | 3.67212E-10 | SIG |
| 92 | TGW | MC | 1 | 231234763 | 2 | 31234763 | 22.3567 | 1.1811 | 1.0526 | 11.2961 | 3.43246E-24 | SIG |
| 93 | TGW | MC | 1 | 334733054 | 3 | 34733054 | 16.5033 | 0.9474 | 0.3712 | 3.9837 | 2.83988E-18 | SIG |
| 94 | TGW | MC | 1 | 335437797 | 3 | 35437797 | 15.9056 | -0.9237 | 0.421 | 4.5181 | 1.14489E-17 | SIG |
| 95 | TGW | MC | 1 | 420926659 | 4 | 20926659 | 6.3254 | -0.5282 | 0.2784 | 2.9874 | 6.77388E-08 | SUG |
| 96 | TGW | MC | 1 | 506624426 | 5 | 6624426 | 7.5295 | -0.5838 | 0.2043 | 2.1922 | 3.89909E-09 | SIG |
| 97 | TGW | MC | 1 | 628503941 | 6 | 28503941 | 22.2022 | 1.1911 | 0.344 | 3.6914 | 4.91549E-24 | SIG |
| 98 | TGW | MC | 1 | 701027206 | 7 | 1027206 | 7.3701 | -0.5363 | 0.2982 | 3.2 | 4.267E-08 | SUG |
| 99 | TGW | MC | 1 | 702255614 | 7 | 2255614 | 7.4953 | -0.585 | 0.3417 | 3.6675 | 4.22791E-09 | SIG |
| 100 | TGW | MC | 1 | 808787015 | 8 | 8787015 | 6.1021 | -0.5248 | 0.1686 | 1.8097 | 1.15188E-07 | SUG |
| 101 | TGW | MC | 1 | 1001426946 | 10 | 1426946 | 23.4889 | -1.2272 | 0.4029 | 4.3238 | 2.47158E-25 | SIG |
| 102 | TGW | MC | 1 | 1017036118 | 10 | 17036118 | 11.2993 | -0.7422 | 0.2879 | 3.09 | 5.45709E-13 | SIG |
| 103 | TGW | MC | 1 | 1211816442 | 12 | 11816442 | 9.2555 | 0.646 | 0.2793 | 2.9973 | 5.55737E-10 | SIG |
| 104 | TGW | MC | 2 | 231332457 | 2 | 31332457 | 11.3805 | 0.7628 | 0.4723 | 5.0398 | 4.16817E-12 | SIG |
| 105 | TGW | MC | 2 | 335461301 | 3 | 35461301 | 8.5083 | -0.6452 | 0.2054 | 2.1919 | 3.86311E-10 | SIG |
| 106 | TGW | MC | 2 | 505651540 | 5 | 5651540 | 5.815 | 0.5251 | 0.2723 | 2.9061 | 2.28199E-07 | SUG |
| 107 | TGW | MC | 2 | 616653419 | 6 | 16653419 | 12.0078 | -0.8067 | 0.66 | 7.0428 | 9.83241E-13 | SIG |
| 108 | TGW | MC | 2 | 628503941 | 6 | 28503941 | 11.7799 | 0.7981 | 0.1544 | 1.6479 | 1.76838E-13 | SIG |
| 109 | TGW | MC | 2 | 701080125 | 7 | 1080125 | 9.1572 | -0.6877 | 0.4026 | 4.2965 | 6.96874E-10 | SIG |
| 110 | TGW | MC | 2 | 801493649 | 8 | 1493649 | 9.5598 | 0.3283 | 0.4734 | 5.0512 | 2.7577E-10 | SIG |
| 111 | TGW | MC | 2 | 808787015 | 8 | 8787015 | 15.4991 | -0.9638 | 0.5688 | 6.0701 | 2.95661E-17 | SIG |
| 112 | TGW | MC | 2 | 808811557 | 8 | 8811557 | 9.7231 | -0.7119 | 0.2837 | 3.0273 | 2.21017E-11 | SIG |
| 113 | TGW | MC | 2 | 906852848 | 9 | 6852848 | 8.6252 | -0.6534 | 0.178 | 1.899 | 2.93244E-10 | SIG |
| 114 | TGW | MC | 2 | 1116334757 | 11 | 16334757 | 12.5064 | 0.8144 | 0.6591 | 7.0335 | 3.11898E-13 | SIG |
| 115 | TGW | MC | 2 | 1223643046 | 12 | 23643046 | 9.6271 | 0.6948 | 0.2382 | 2.542 | 2.77058E-11 | SIG |
| 116 | TGW | MC | MEJA | 104407547 | 1 | 4407547 | 13.7283 | 0.4186 | 0.0421 | 0.4527 | 1.8498E-15 | SIG |
| 117 | TGW | MC | MEJA | 139046778 | 1 | 39046778 | 21.4332 | -0.5408 | 0.1044 | 1.1223 | 2.93821E-23 | SIG |
| 118 | TGW | MC | MEJA | 204346020 | 2 | 4346020 | 11.6238 | 0.3791 | 0.0249 | 0.2681 | 2.5497E-13 | SIG |
| 119 | TGW | MC | MEJA | 204706818 | 2 | 4706818 | 7.7962 | -0.3092 | 0.0905 | 0.9728 | 1.59974E-08 | SIG |
| 120 | TGW | MC | MEJA | 224553100 | 2 | 24553100 | 43.6124 | -0.8766 | 0.229 | 2.4612 | 1.37287E-45 | SIG |
| 121 | TGW | MC | MEJA | 231234763 | 2 | 31234763 | 10.5087 | 0.3567 | 0.096 | 1.0316 | 3.48902E-12 | SIG |
| 122 | TGW | MC | MEJA | 231262967 | 2 | 31262967 | 10.5087 | 0.3567 | 0.096 | 1.0316 | 3.48902E-12 | SIG |
| 123 | TGW | MC | MEJA | 235005427 | 2 | 35005427 | 8.0469 | 0.3123 | 0.0798 | 0.8579 | 1.14774E-09 | SIG |
| 124 | TGW | MC | MEJA | 308081540 | 3 | 8081540 | 25.5102 | 0.606 | 0.1847 | 1.9854 | 3.09571E-26 | SIG |
| 125 | TGW | MC | MEJA | 335486847 | 3 | 35486847 | 12.3697 | -0.3927 | 0.0806 | 0.866 | 4.44176E-14 | SIG |
| 126 | TGW | MC | MEJA | 431318603 | 4 | 31318603 | 59.0925 | -1.1367 | 0.4012 | 4.3112 | 8.12264E-60 | SIG |
| 127 | TGW | MC | MEJA | 510518812 | 5 | 10518812 | 10.7691 | 0.3683 | 0.046 | 0.494 | 1.89306E-12 | SIG |
| 128 | TGW | MC | MEJA | 600023671 | 6 | 23671 | 15.603 | -0.4599 | 0.2071 | 2.2255 | 2.498E-16 | SIG |
| 129 | TGW | MC | MEJA | 628503941 | 6 | 28503941 | 41.0574 | 0.8493 | 0.1749 | 1.8794 | 5.07504E-43 | SIG |
| 130 | TGW | MC | MEJA | 701080125 | 7 | 1080125 | 17.8836 | -0.491 | 0.2072 | 2.2272 | 1.30941E-18 | SIG |
| 131 | TGW | MC | MEJA | 710299436 | 7 | 10299436 | 20.5664 | -0.5272 | 0.0478 | 0.5139 | 2.20617E-22 | SIG |
| 132 | TGW | MC | MEJA | 903772207 | 9 | 3772207 | 13.8503 | -0.4076 | 0.1047 | 1.1252 | 1.41315E-14 | SIG |
| 133 | TGW | MC | MEJA | 904639851 | 9 | 4639851 | 14.5337 | 0.2601 | 0.1778 | 1.9107 | 2.92956E-15 | SIG |
| 134 | TGW | MC | MEJA | 904826405 | 9 | 4826405 | 18.9444 | 0.506 | 0.0697 | 0.7486 | 9.61653E-21 | SIG |
| 135 | TGW | MC | MEJA | 1001426946 | 10 | 1426946 | 7.5405 | -0.2974 | 0.0237 | 0.2543 | 3.79906E-09 | SIG |
| 136 | TGW | MC | MEJA | 1013915559 | 10 | 13915559 | 33.6255 | -0.7147 | 0.3675 | 3.949 | 2.37551E-34 | SIG |
| 137 | TGW | MC | MEJA | 1016131068 | 10 | 16131068 | 19.0549 | -0.5055 | 0.0689 | 0.7407 | 7.43576E-21 | SIG |
| 138 | TGW | MC | MEJA | 1020319057 | 10 | 20319057 | 16.3725 | 0.4726 | 0.0556 | 0.5973 | 3.85328E-18 | SIG |
| 139 | TGW | MC | MEJA | 1116334757 | 11 | 16334757 | 24.0824 | 0.5843 | 0.3344 | 3.5933 | 8.28821E-25 | SIG |
| 140 | TGW | MC | MEJA | 1117803774 | 11 | 17803774 | 7.6668 | 0.3 | 0.0704 | 0.7562 | 2.15539E-08 | SIG |
| 141 | GW | RR | 1 | 225565194 | 2 | 25565194 | 11.3265 | -0.0554 | 0.0007 | 0.886 | 5.1195E-13 | SIG |
| 142 | GW | RR | 1 | 316415833 | 3 | 16415833 | 6.2872 | -0.0386 | 0.0014 | 1.6544 | 5.16458E-07 | SUG |
| 143 | GW | RR | 1 | 505357438 | 5 | 5357438 | 28.69 | -0.1003 | 0.0086 | 10.2534 | 1.41023E-30 | SIG |
| 144 | GW | RR | 1 | 513900081 | 5 | 13900081 | 4.7854 | 0.0337 | 0.0011 | 1.3438 | 2.67503E-06 | SUG |
| 145 | GW | RR | 1 | 725284287 | 7 | 25284287 | 10.7505 | 0.0546 | 0.0008 | 0.9663 | 1.77772E-11 | SIG |
| 146 | GW | RR | 1 | 805969839 | 8 | 5969839 | 6.4855 | 0.0401 | 0.0013 | 1.5469 | 3.27178E-07 | SUG |
| 147 | GW | RR | 1 | 906837440 | 9 | 6837440 | 18.7435 | 0.0747 | 0.0022 | 2.5865 | 1.80818E-19 | SIG |
| 148 | GW | RR | 1 | 1003262163 | 10 | 3262163 | 27.3216 | 0.0994 | 0.0011 | 1.2962 | 4.77936E-28 | SIG |
| 149 | GW | RR | 1 | 1004502295 | 10 | 4502295 | 3.6691 | 0.0299 | 0.0007 | 0.857 | 3.94823E-05 | SUG |
| 150 | GW | RR | 1 | 1107818816 | 11 | 7818816 | 9.4607 | -0.0493 | 0.0019 | 2.2536 | 4.09751E-11 | SIG |
| 151 | GW | RR | 1 | 1116768397 | 11 | 16768397 | 22.6673 | -0.0848 | 0.0018 | 2.1254 | 2.15521E-23 | SIG |
| 152 | GW | RR | 1 | 1118701082 | 11 | 18701082 | 10.1386 | -0.0511 | 0.0019 | 2.2596 | 8.32305E-12 | SIG |
| 153 | GW | RR | 1 | 1122223115 | 11 | 22223115 | 18.3276 | -0.0737 | 0.0012 | 1.3951 | 4.04472E-20 | SIG |
| 154 | GW | RR | 1 | 1125125749 | 11 | 25125749 | 8.4956 | -0.0462 | 0.0013 | 1.5143 | 3.98124E-10 | SIG |
| 155 | GW | RR | 1 | 1210386656 | 12 | 10386656 | 8.2023 | 0.0455 | 0.0019 | 2.2098 | 7.95352E-10 | SIG |
| 156 | GW | RR | 2 | 140476070 | 1 | 40476070 | 11.7326 | -0.0442 | 0.002 | 2.5295 | 1.853E-12 | SIG |
| 157 | GW | RR | 2 | 217616900 | 2 | 17616900 | 30.5225 | 0.0876 | 0.0027 | 3.4194 | 3.01063E-31 | SIG |
| 158 | GW | RR | 2 | 223888162 | 2 | 23888162 | 12.5484 | -0.0467 | 0.0008 | 1.0061 | 2.92333E-14 | SIG |
| 159 | GW | RR | 2 | 336328500 | 3 | 36328500 | 12.297 | 0.0469 | 0.0017 | 2.1888 | 5.26624E-14 | SIG |
| 160 | GW | RR | 2 | 422072891 | 4 | 22072891 | 7.7731 | 0.0364 | 0.001 | 1.3253 | 1.6871E-08 | SIG |
| 161 | GW | RR | 2 | 505358771 | 5 | 5358771 | 17.4274 | -0.0573 | 0.0029 | 3.7074 | 3.74352E-18 | SIG |
| 162 | GW | RR | 2 | 506015422 | 5 | 6015422 | 11.791 | -0.0458 | 0.0021 | 2.6493 | 1.72332E-13 | SIG |
| 163 | GW | RR | 2 | 521088250 | 5 | 21088250 | 19.2727 | -0.0597 | 0.0022 | 2.7465 | 5.34618E-20 | SIG |
| 164 | GW | RR | 2 | 725127139 | 7 | 25127139 | 26.8931 | 0.0773 | 0.0014 | 1.8287 | 9.11966E-29 | SIG |
| 165 | GW | RR | 2 | 907639381 | 9 | 7639381 | 16.5776 | 0.0566 | 0.0012 | 1.5401 | 2.38841E-18 | SIG |
| 166 | GW | RR | 2 | 1022038949 | 10 | 22038949 | 15.3988 | -0.0535 | 0.0025 | 3.2285 | 3.7359E-17 | SIG |
| 167 | GW | RR | 2 | 1113995468 | 11 | 13995468 | 51.4814 | -0.1328 | 0.0026 | 3.3567 | 3.31519E-52 | SIG |
| 168 | GW | RR | 2 | 1118701082 | 11 | 18701082 | 14.9619 | -0.0519 | 0.002 | 2.5022 | 1.03611E-16 | SIG |
| 169 | GW | RR | 2 | 1125735896 | 11 | 25735896 | 11.162 | -0.0443 | 0.0011 | 1.3592 | 7.52936E-13 | SIG |
| 170 | GW | RR | 2 | 1210711089 | 12 | 10711089 | 15.7479 | 0.0543 | 0.0025 | 3.2509 | 1.65438E-17 | SIG |
| 171 | GW | RR | 2 | 1227465140 | 12 | 27465140 | 19.4722 | 0.0625 | 0.0014 | 1.8133 | 3.37701E-20 | SIG |
| 172 | GW | RR | MEJA | 105421087 | 1 | 5421087 | 9.1685 | 0.0043 | 0.0005 | 0.5665 | 6.78879E-10 | SIG |
| 173 | GW | RR | MEJA | 204994560 | 2 | 4994560 | 15.2559 | -0.0282 | 0.0008 | 0.9701 | 5.21548E-17 | SIG |
| 174 | GW | RR | MEJA | 225565194 | 2 | 25565194 | 33.4266 | -0.0454 | 0.0005 | 0.6123 | 2.39939E-35 | SIG |
| 175 | GW | RR | MEJA | 229241853 | 2 | 29241853 | 17.1014 | 0.0307 | 0.0004 | 0.4814 | 7.04125E-19 | SIG |
| 176 | GW | RR | MEJA | 303390977 | 3 | 3390977 | 26.1316 | 0.0384 | 0.0006 | 0.689 | 5.34064E-28 | SIG |
| 177 | GW | RR | MEJA | 316504015 | 3 | 16504015 | 15.0212 | -0.0286 | 0.0007 | 0.8555 | 9.53546E-16 | SIG |
| 178 | GW | RR | MEJA | 331660510 | 3 | 31660510 | 5.5412 | -0.0164 | 0.0003 | 0.3284 | 4.38451E-07 | SUG |
| 179 | GW | RR | MEJA | 404722231 | 4 | 4722231 | 11.0907 | -0.0236 | 0.0003 | 0.3133 | 8.90158E-13 | SIG |
| 180 | GW | RR | MEJA | 413817255 | 4 | 13817255 | 12.7843 | -0.0256 | 0.0005 | 0.5537 | 1.68284E-14 | SIG |
| 181 | GW | RR | MEJA | 505323603 | 5 | 5323603 | 7.3103 | 0.019 | 0.0004 | 0.4578 | 4.89698E-08 | SUG |
| 182 | GW | RR | MEJA | 505358236 | 5 | 5358236 | 52.7153 | -0.0606 | 0.0032 | 3.9152 | 9.8672E-55 | SIG |
| 183 | GW | RR | MEJA | 513900081 | 5 | 13900081 | 5.1303 | 0.0157 | 0.0002 | 0.3004 | 1.17065E-06 | SUG |
| 184 | GW | RR | MEJA | 521088250 | 5 | 21088250 | 22.6226 | -0.0331 | 0.0008 | 0.987 | 2.38893E-23 | SIG |
| 185 | GW | RR | MEJA | 725284287 | 7 | 25284287 | 28.1824 | 0.0405 | 0.0004 | 0.5154 | 6.58566E-29 | SIG |
| 186 | GW | RR | MEJA | 800487221 | 8 | 487221 | 29.7484 | -0.0418 | 0.0005 | 0.5848 | 1.78941E-30 | SIG |
| 187 | GW | RR | MEJA | 806166430 | 8 | 6166430 | 11.4811 | 0.0241 | 0.0004 | 0.5207 | 3.30643E-12 | SIG |
| 188 | GW | RR | MEJA | 808915046 | 8 | 8915046 | 10.4167 | -0.023 | 0.0005 | 0.5798 | 3.83392E-11 | SIG |
| 189 | GW | RR | MEJA | 812442028 | 8 | 12442028 | 17.428 | 0.0299 | 0.0004 | 0.5018 | 3.7379E-18 | SIG |
| 190 | GW | RR | MEJA | 817510908 | 8 | 17510908 | 29.4902 | -0.0418 | 0.0016 | 2.0096 | 3.24273E-30 | SIG |
| 191 | GW | RR | MEJA | 819654130 | 8 | 19654130 | 7.4181 | 0.0197 | 0.0002 | 0.3051 | 3.82056E-08 | SUG |
| 192 | GW | RR | MEJA | 907342896 | 9 | 7342896 | 23.5331 | 0.0363 | 0.0006 | 0.7171 | 2.23039E-25 | SIG |
| 193 | GW | RR | MEJA | 1011991995 | 10 | 11991995 | 9.7444 | 0.022 | 0.0005 | 0.5772 | 2.1023E-11 | SIG |
| 194 | GW | RR | MEJA | 1021983205 | 10 | 21983205 | 22.8753 | -0.0354 | 0.0011 | 1.392 | 1.33508E-23 | SIG |
| 195 | GW | RR | MEJA | 1106648699 | 11 | 6648699 | 16.1171 | -0.029 | 0.0003 | 0.3096 | 6.99048E-18 | SIG |
| 196 | GW | RR | MEJA | 1116528947 | 11 | 16528947 | 17.418 | -0.0283 | 0.0005 | 0.6476 | 3.8247E-18 | SIG |
| 197 | GW | RR | MEJA | 1116768397 | 11 | 16768397 | 54.8819 | -0.0616 | 0.0012 | 1.4738 | 1.31854E-55 | SIG |
| 198 | GW | RR | MEJA | 1118761608 | 11 | 18761608 | 61.9895 | -0.0665 | 0.0009 | 1.1315 | 1.02982E-62 | SIG |
| 199 | GW | RR | MEJA | 1125735896 | 11 | 25735896 | 43.8426 | -0.0541 | 0.0016 | 1.9532 | 8.058E-46 | SIG |
| 200 | GW | RR | MEJA | 1126349247 | 11 | 26349247 | 19.5934 | 0.0324 | 0.001 | 1.2752 | 2.12291E-21 | SIG |
| 201 | GW | RR | MEJA | 1209492644 | 12 | 9492644 | 10.7003 | 0.0232 | 0.0005 | 0.6191 | 2.22497E-12 | SIG |
| 202 | GL | RR | 1 | 125212877 | 1 | 25212877 | 13.7046 | -0.1312 | 0.0059 | 0.9399 | 1.95511E-15 | SIG |
| 203 | GL | RR | 1 | 206168637 | 2 | 6168637 | 5.558 | 0.0795 | 0.0062 | 0.9813 | 4.21262E-07 | SUG |
| 204 | GL | RR | 1 | 207093706 | 2 | 7093706 | 16.1357 | 0.1446 | 0.0103 | 1.6444 | 6.69336E-18 | SIG |
| 205 | GL | RR | 1 | 217474355 | 2 | 17474355 | 14.3413 | 0.1348 | 0.009 | 1.4378 | 4.41492E-16 | SIG |
| 206 | GL | RR | 1 | 231629057 | 2 | 31629057 | 11.6607 | 0.1187 | 0.0086 | 1.372 | 2.33835E-13 | SIG |
| 207 | GL | RR | 1 | 316746142 | 3 | 16746142 | 26.1652 | 0.1994 | 0.0381 | 6.0501 | 4.93987E-28 | SIG |
| 208 | GL | RR | 1 | 321140760 | 3 | 21140760 | 17.0132 | 0.1495 | 0.0188 | 2.994 | 8.64956E-19 | SIG |
| 209 | GL | RR | 1 | 334113758 | 3 | 34113758 | 19.7123 | 0.1649 | 0.0052 | 0.8308 | 1.60971E-21 | SIG |
| 210 | GL | RR | 1 | 401015786 | 4 | 1015786 | 16.1207 | -0.1445 | 0.0096 | 1.5217 | 6.93276E-18 | SIG |
| 211 | GL | RR | 1 | 406883844 | 4 | 6883844 | 17.339 | -0.1536 | 0.007 | 1.1088 | 4.58853E-18 | SIG |
| 212 | GL | RR | 1 | 431723084 | 4 | 31723084 | 10.6102 | -0.1124 | 0.0033 | 0.5168 | 2.74877E-12 | SIG |
| 213 | GL | RR | 1 | 724964429 | 7 | 24964429 | 20.7783 | -0.1707 | 0.0088 | 1.3992 | 1.34775E-22 | SIG |
| 214 | GL | RR | 1 | 820722975 | 8 | 20722975 | 20.4004 | -0.1681 | 0.0198 | 3.1475 | 3.24617E-22 | SIG |
| 215 | GL | RR | 1 | 906961517 | 9 | 6961517 | 11.9567 | 0.1205 | 0.0061 | 0.9714 | 1.16883E-13 | SIG |
| 216 | GL | RR | 1 | 1013888948 | 10 | 13888948 | 8.2083 | -0.097 | 0.0036 | 0.5717 | 7.84058E-10 | SIG |
| 217 | GL | RR | 1 | 1018229059 | 10 | 18229059 | 9.8837 | -0.1078 | 0.0077 | 1.2161 | 1.51503E-11 | SIG |
| 218 | GL | RR | 1 | 1118043760 | 11 | 18043760 | 7.7372 | -0.0954 | 0.0069 | 1.0941 | 2.38593E-09 | SIG |
| 219 | GL | RR | 1 | 1219921339 | 12 | 19921339 | 24.9033 | -0.1972 | 0.0137 | 2.1729 | 1.25204E-25 | SIG |
| 220 | GL | RR | 2 | 125212877 | 1 | 25212877 | 24.6341 | -0.2021 | 0.014 | 2.1294 | 1.72846E-26 | SIG |
| 221 | GL | RR | 2 | 316708508 | 3 | 16708508 | 46.204 | 0.3311 | 0.1055 | 15.9983 | 3.41725E-48 | SIG |
| 222 | GL | RR | 2 | 334161467 | 3 | 34161467 | 17.0358 | 0.1582 | 0.0101 | 1.5328 | 8.2049E-19 | SIG |
| 223 | GL | RR | 2 | 405112378 | 4 | 5112378 | 20.4127 | -0.1773 | 0.006 | 0.9112 | 3.15423E-22 | SIG |
| 224 | GL | RR | 2 | 406883844 | 4 | 6883844 | 16.9095 | -0.1571 | 0.0085 | 1.286 | 1.23346E-17 | SIG |
| 225 | GL | RR | 2 | 505413770 | 5 | 5413770 | 14.3552 | 0.1418 | 0.0117 | 1.7693 | 4.27416E-16 | SIG |
| 226 | GL | RR | 2 | 520889199 | 5 | 20889199 | 17.7093 | 0.1658 | 0.0059 | 0.899 | 1.95588E-18 | SIG |
| 227 | GL | RR | 2 | 601623846 | 6 | 1623846 | 8.2691 | -0.1027 | 0.0105 | 1.5926 | 6.79347E-10 | SIG |
| 228 | GL | RR | 2 | 606777639 | 6 | 6777639 | 11.6929 | 0.1262 | 0.0142 | 2.1592 | 2.16849E-13 | SIG |
| 229 | GL | RR | 2 | 820715449 | 8 | 20715449 | 13.6741 | -0.1418 | 0.0115 | 1.7488 | 2.1205E-14 | SIG |
| 230 | GL | RR | 2 | 920512658 | 9 | 20512658 | 5.5378 | -0.0831 | 0.0041 | 0.6177 | 4.42035E-07 | SUG |
| 231 | GL | RR | 2 | 1014508748 | 10 | 14508748 | 8.1257 | -0.1017 | 0.0094 | 1.4201 | 9.52897E-10 | SIG |
| 232 | GL | RR | 2 | 1108501237 | 11 | 8501237 | 12.5957 | -0.1405 | 0.0094 | 1.4295 | 2.53936E-13 | SIG |
| 233 | GL | RR | 2 | 1117830860 | 11 | 17830860 | 5.5426 | 0.0824 | 0.0065 | 0.9799 | 4.37025E-07 | SUG |
| 234 | GL | RR | 2 | 1118586493 | 11 | 18586493 | 3.8131 | -0.0677 | 0.0041 | 0.6253 | 2.78514E-05 | SUG |
| 235 | GL | RR | MEJA | 106036854 | 1 | 6036854 | 13.6295 | -0.0601 | 0.0033 | 0.5089 | 2.33027E-15 | SIG |
| 236 | GL | RR | MEJA | 123144702 | 1 | 23144702 | 40.4568 | -0.1161 | 0.0055 | 0.8595 | 3.50537E-41 | SIG |
| 237 | GL | RR | MEJA | 204868399 | 2 | 4868399 | 13.3913 | -0.0597 | 0.0032 | 0.4902 | 4.06751E-15 | SIG |
| 238 | GL | RR | MEJA | 316708508 | 3 | 16708508 | 119.44 | 0.2772 | 0.0739 | 11.5002 | 1.2455E-121 | SIG |
| 239 | GL | RR | MEJA | 321140760 | 3 | 21140760 | 16.7587 | 0.0675 | 0.0038 | 0.5967 | 1.56554E-18 | SIG |
| 240 | GL | RR | MEJA | 334087282 | 3 | 34087282 | 55.8497 | 0.1436 | 0.0039 | 0.6133 | 7.03947E-58 | SIG |
| 241 | GL | RR | MEJA | 401015786 | 4 | 1015786 | 8.1228 | -0.0455 | 0.0009 | 0.1475 | 9.59444E-10 | SIG |
| 242 | GL | RR | MEJA | 403855798 | 4 | 3855798 | 72.4213 | -0.1753 | 0.0072 | 1.1265 | 1.66151E-74 | SIG |
| 243 | GL | RR | MEJA | 404591488 | 4 | 4591488 | 15.573 | 0.0647 | 0.0018 | 0.2868 | 2.4881E-17 | SIG |
| 244 | GL | RR | MEJA | 406883844 | 4 | 6883844 | 29.7445 | -0.0948 | 0.003 | 0.4691 | 1.8054E-30 | SIG |
| 245 | GL | RR | MEJA | 409493312 | 4 | 9493312 | 9.6352 | -0.0516 | 0.0009 | 0.143 | 2.31824E-10 | SIG |
| 246 | GL | RR | MEJA | 435129844 | 4 | 35129844 | 34.6613 | -0.1043 | 0.0044 | 0.6827 | 1.37316E-36 | SIG |
| 247 | GL | RR | MEJA | 505914985 | 5 | 5914985 | 48.789 | 0.1308 | 0.0059 | 0.9154 | 8.65105E-51 | SIG |
| 248 | GL | RR | MEJA | 606533624 | 6 | 6533624 | 14.0836 | 0.0622 | 0.0024 | 0.3777 | 8.06177E-16 | SIG |
| 249 | GL | RR | MEJA | 606777283 | 6 | 6777283 | 10.2356 | 0.0514 | 0.0024 | 0.366 | 6.62709E-12 | SIG |
| 250 | GL | RR | MEJA | 614171782 | 6 | 14171782 | 10.1675 | -0.0513 | 0.0024 | 0.3667 | 7.77554E-12 | SIG |
| 251 | GL | RR | MEJA | 627045117 | 6 | 27045117 | 35.5486 | -0.1059 | 0.0029 | 0.4524 | 1.75788E-37 | SIG |
| 252 | GL | RR | MEJA | 719727567 | 7 | 19727567 | 27.594 | -0.0908 | 0.0025 | 0.3895 | 1.79297E-29 | SIG |
| 253 | GL | RR | MEJA | 724620377 | 7 | 24620377 | 43.7233 | -0.1214 | 0.0057 | 0.8801 | 1.0621E-45 | SIG |
| 254 | GL | RR | MEJA | 803954829 | 8 | 3954829 | 15.6762 | -0.065 | 0.0033 | 0.5183 | 1.95548E-17 | SIG |
| 255 | GL | RR | MEJA | 820719085 | 8 | 20719085 | 16.7466 | -0.0669 | 0.0031 | 0.4848 | 1.79495E-17 | SIG |
| 256 | GL | RR | MEJA | 906986093 | 9 | 6986093 | 22.4094 | 0.0799 | 0.0028 | 0.4397 | 3.03668E-24 | SIG |
| 257 | GL | RR | MEJA | 916419362 | 9 | 16419362 | 28.3219 | 0.0919 | 0.0027 | 0.4248 | 3.31249E-30 | SIG |
| 258 | GL | RR | MEJA | 920512658 | 9 | 20512658 | 23.6421 | -0.083 | 0.0041 | 0.6317 | 1.73136E-25 | SIG |
| 259 | GL | RR | MEJA | 1014508748 | 10 | 14508748 | 14.1303 | -0.0613 | 0.0034 | 0.5296 | 7.22814E-16 | SIG |
| 260 | GL | RR | MEJA | 1108501237 | 11 | 8501237 | 20.1412 | -0.0772 | 0.0026 | 0.4105 | 7.23608E-21 | SIG |
| 261 | GL | RR | MEJA | 1117830860 | 11 | 17830860 | 28.8801 | 0.0927 | 0.0082 | 1.2733 | 9.07467E-31 | SIG |
| 262 | GL | RR | MEJA | 1118586493 | 11 | 18586493 | 21.562 | -0.0782 | 0.0055 | 0.8553 | 2.1776E-23 | SIG |
| 263 | GL | RR | MEJA | 1120155299 | 11 | 20155299 | 42.6161 | -0.1281 | 0.0024 | 0.3676 | 2.42935E-43 | SIG |
| 264 | GL | RR | MEJA | 1200043637 | 12 | 43637 | 11.5608 | 0.0555 | 0.0011 | 0.1762 | 2.9553E-13 | SIG |
| 265 | GL | RR | MEJA | 1202930390 | 12 | 2930390 | 20.9647 | 0.0767 | 0.0026 | 0.4021 | 8.73542E-23 | SIG |
| 266 | TGW | RR | 1 | 111524890 | 1 | 11524890 | 9.1316 | 0.5829 | 0.2335 | 2.4494 | 7.39202E-10 | SIG |
| 267 | TGW | RR | 1 | 129860670 | 1 | 29860670 | 27.4666 | -1.2301 | 0.3232 | 3.3906 | 2.40969E-29 | SIG |
| 268 | TGW | RR | 1 | 201230631 | 2 | 1230631 | 14.6089 | 0.8175 | 0.246 | 2.5811 | 2.36288E-16 | SIG |
| 269 | TGW | RR | 1 | 226049877 | 2 | 26049877 | 29.3821 | -1.3333 | 0.4952 | 5.195 | 4.15898E-30 | SIG |
| 270 | TGW | RR | 1 | 231102971 | 2 | 31102971 | 10.8632 | 0.6784 | 0.3287 | 3.4486 | 1.51787E-12 | SIG |
| 271 | TGW | RR | 1 | 404570606 | 4 | 4570606 | 14.2645 | -0.798 | 0.2432 | 2.5515 | 5.28231E-16 | SIG |
| 272 | TGW | RR | 1 | 431723084 | 4 | 31723084 | 48.6178 | -1.9607 | 0.9902 | 10.388 | 1.28518E-50 | SIG |
| 273 | TGW | RR | 1 | 505895833 | 5 | 5895833 | 5.0426 | 0.4424 | 0.1944 | 2.0398 | 1.44398E-06 | SUG |
| 274 | TGW | RR | 1 | 612697211 | 6 | 12697211 | 21.9412 | 1.0513 | 0.2361 | 2.4767 | 9.01799E-24 | SIG |
| 275 | TGW | RR | 1 | 615626457 | 6 | 15626457 | 12.2696 | -0.7475 | 0.2989 | 3.1354 | 5.38128E-13 | SIG |
| 276 | TGW | RR | 1 | 810690144 | 8 | 10690144 | 4.8643 | -0.4367 | 0.1448 | 1.5195 | 2.21385E-06 | SUG |
| 277 | TGW | RR | 1 | 1010606927 | 10 | 10606927 | 8.4875 | 0.5908 | 0.2683 | 2.8148 | 4.05769E-10 | SIG |
| 278 | TGW | RR | 1 | 1103362215 | 11 | 3362215 | 15.2289 | 0.836 | 0.2416 | 2.5342 | 5.55424E-17 | SIG |
| 279 | TGW | RR | 1 | 1113681900 | 11 | 13681900 | 20.6606 | -1.0098 | 0.1949 | 2.0446 | 1.77198E-22 | SIG |
| 280 | TGW | RR | 1 | 1113977759 | 11 | 13977759 | 8.0962 | 0.5741 | 0.3288 | 3.4495 | 8.0188E-09 | SIG |
| 281 | TGW | RR | 1 | 1200833377 | 12 | 833377 | 16.6818 | 0.8793 | 0.3546 | 3.7194 | 1.87315E-18 | SIG |
| 282 | TGW | RR | 2 | 122612838 | 1 | 22612838 | 25.2357 | 0.9938 | 0.2328 | 2.895 | 4.27432E-27 | SIG |
| 283 | TGW | RR | 2 | 126809821 | 1 | 26809821 | 6.5098 | -0.463 | 0.1812 | 2.2534 | 3.09371E-07 | SUG |
| 284 | TGW | RR | 2 | 140053361 | 1 | 40053361 | 13.9971 | 0.6792 | 0.4614 | 5.7361 | 9.86817E-16 | SIG |
| 285 | TGW | RR | 2 | 225038930 | 2 | 25038930 | 21.5681 | -0.9163 | 0.2752 | 3.4211 | 2.70813E-22 | SIG |
| 286 | TGW | RR | 2 | 431939665 | 4 | 31939665 | 12.4957 | -0.6344 | 0.1216 | 1.5113 | 3.30691E-14 | SIG |
| 287 | TGW | RR | 2 | 501981355 | 5 | 1981355 | 33.5621 | 1.2315 | 0.3597 | 4.4718 | 1.75291E-35 | SIG |
| 288 | TGW | RR | 2 | 511152752 | 5 | 11152752 | 33.0985 | -1.2139 | 0.4636 | 5.7634 | 7.99348E-34 | SIG |
| 289 | TGW | RR | 2 | 611905299 | 6 | 11905299 | 16.8987 | -0.7609 | 0.2065 | 2.567 | 1.26441E-17 | SIG |
| 290 | TGW | RR | 2 | 628657023 | 6 | 28657023 | 11.5382 | 0.6052 | 0.3615 | 4.494 | 2.89892E-12 | SIG |
| 291 | TGW | RR | 2 | 708761424 | 7 | 8761424 | 21.7612 | -0.8699 | 0.1804 | 2.2431 | 1.73631E-22 | SIG |
| 292 | TGW | RR | 2 | 808395003 | 8 | 8395003 | 11.8312 | -0.618 | 0.2301 | 2.8606 | 1.47639E-12 | SIG |
| 293 | TGW | RR | 2 | 826111247 | 8 | 26111247 | 28.202 | 1.0764 | 0.2984 | 3.7103 | 4.37429E-30 | SIG |
| 294 | TGW | RR | 2 | 920866738 | 9 | 20866738 | 23.7992 | 0.954 | 0.2145 | 2.6674 | 1.20191E-25 | SIG |
| 295 | TGW | RR | 2 | 1016892211 | 10 | 16892211 | 17.8409 | -0.6998 | 0.3705 | 4.607 | 1.4446E-18 | SIG |
| 296 | TGW | RR | 2 | 1103476187 | 11 | 3476187 | 12.9448 | -0.6459 | 0.2554 | 3.1759 | 1.1559E-14 | SIG |
| 297 | TGW | RR | 2 | 1118422045 | 11 | 18422045 | 19.581 | -0.8392 | 0.5922 | 7.3634 | 2.62877E-20 | SIG |
| 298 | TGW | RR | 2 | 1124346080 | 11 | 24346080 | 7.3689 | -0.4671 | 0.218 | 2.71 | 5.70206E-09 | SIG |
| 299 | TGW | RR | MEJA | 100171150 | 1 | 171150 | 7.2519 | 0.0324 | 0.1082 | 1.2237 | 5.60194E-08 | SUG |
| 300 | TGW | RR | MEJA | 107261596 | 1 | 7261596 | 7.3262 | -0.3351 | 0.0567 | 0.6417 | 6.30859E-09 | SIG |
| 301 | TGW | RR | MEJA | 121465134 | 1 | 21465134 | 9.2079 | 0.3807 | 0.1101 | 1.2453 | 7.43027E-11 | SIG |
| 302 | TGW | RR | MEJA | 135730043 | 1 | 35730043 | 8.0219 | -0.3564 | 0.12 | 1.3574 | 9.51476E-09 | SIG |
| 303 | TGW | RR | MEJA | 140086802 | 1 | 40086802 | 15.3981 | 0.4902 | 0.2442 | 2.7622 | 4.00412E-16 | SIG |
| 304 | TGW | RR | MEJA | 201230631 | 2 | 1230631 | 16.1277 | 0.512 | 0.0965 | 1.0916 | 6.81932E-18 | SIG |
| 305 | TGW | RR | MEJA | 313505333 | 3 | 13505333 | 13.7785 | 0.4519 | 0.1165 | 1.3172 | 1.66721E-14 | SIG |
| 306 | TGW | RR | MEJA | 315602952 | 3 | 15602952 | 29.7897 | 0.7285 | 0.1932 | 2.1851 | 1.10039E-31 | SIG |
| 307 | TGW | RR | MEJA | 404570606 | 4 | 4570606 | 21.6974 | -0.6008 | 0.1379 | 1.5592 | 1.58962E-23 | SIG |
| 308 | TGW | RR | MEJA | 425092933 | 4 | 25092933 | 19.9604 | 0.597 | 0.0686 | 0.7759 | 1.09722E-20 | SIG |
| 309 | TGW | RR | MEJA | 432466735 | 4 | 32466735 | 45.3429 | -0.95 | 0.4053 | 4.5838 | 4.55835E-46 | SIG |
| 310 | TGW | RR | MEJA | 501568614 | 5 | 1568614 | 9.8156 | 0.3877 | 0.0948 | 1.0722 | 1.77811E-11 | SIG |
| 311 | TGW | RR | MEJA | 514579088 | 5 | 14579088 | 15.4061 | -0.5007 | 0.0824 | 0.9318 | 3.67341E-17 | SIG |
| 312 | TGW | RR | MEJA | 610584698 | 6 | 10584698 | 37.0771 | -0.8336 | 0.3802 | 4.3 | 5.09955E-39 | SIG |
| 313 | TGW | RR | MEJA | 816572646 | 8 | 16572646 | 27.9384 | -0.7058 | 0.0978 | 1.1059 | 1.15519E-28 | SIG |
| 314 | TGW | RR | MEJA | 817696038 | 8 | 17696038 | 5.2885 | 0.2853 | 0.0814 | 0.9206 | 8.01858E-07 | SUG |
| 315 | TGW | RR | MEJA | 905251526 | 9 | 5251526 | 7.4565 | 0.3404 | 0.1009 | 1.141 | 4.63417E-09 | SIG |
| 316 | TGW | RR | MEJA | 919843818 | 9 | 19843818 | 34.9536 | 0.8418 | 0.1745 | 1.9736 | 1.11605E-35 | SIG |
| 317 | TGW | RR | MEJA | 1010926362 | 10 | 10926362 | 5.5627 | -0.276 | 0.0495 | 0.5602 | 2.73834E-06 | SUG |
| 318 | TGW | RR | MEJA | 1103320017 | 11 | 3320017 | 39.2364 | 0.8676 | 0.2113 | 2.3899 | 3.43722E-41 | SIG |
| 319 | TGW | RR | MEJA | 1103518389 | 11 | 3518389 | 28.5826 | -0.708 | 0.3147 | 3.5595 | 1.80936E-30 | SIG |
| 320 | TGW | RR | MEJA | 1116085870 | 11 | 16085870 | 4.0992 | 0.2485 | 0.0609 | 0.6887 | 1.39438E-05 | SUG |
| 321 | TGW | RR | MEJA | 1117131649 | 11 | 17131649 | 15.4395 | 0.5045 | 0.0792 | 0.8953 | 3.63954E-16 | SIG |
| 322 | TGW | RR | MEJA | 1118422045 | 11 | 18422045 | 4.9968 | -0.2715 | 0.0623 | 0.705 | 1.00772E-05 | SUG |
| 323 | TGW | RR | MEJA | 1222584177 | 12 | 22584177 | 14.7554 | -0.4826 | 0.1194 | 1.3504 | 1.67813E-16 | SIG |

GW: grain width; GL: grain length; TGW: thousand grain weight; MC: main crop; RR: ratoon rice; MEJA: multi-environment joint analysis

**Table S24 Main-effect QTN for grain width, grain length, and thousand grain weight in main crop and ratoon rice of 159 rice accessions in single environment and multi-environment joint analysis using Q matrix**

| **No.** | **Trait** | **MC or RR** | **Environment** | **Marker** | **Chromosome** | **Position (bp)** | **LOD** | **add** | **variance** | **r2(%)** | **P-value** | **significance** |
| --- | --- | --- | --- | --- | --- | --- | --- | --- | --- | --- | --- | --- |
| 1 | GW | MC | 1 | 140476043 | 1 | 40476043 | 17.1236 | 0.0711 | 0.0049 | 5.6146 | 7.53381E-18 | SIG |
| 2 | GW | MC | 1 | 326622444 | 3 | 26622444 | 6.6722 | -0.0392 | 0.0012 | 1.3527 | 2.97209E-08 | SUG |
| 3 | GW | MC | 1 | 331660510 | 3 | 31660510 | 6.0965 | -0.0374 | 0.0013 | 1.4921 | 1.16733E-07 | SUG |
| 4 | GW | MC | 1 | 431159467 | 4 | 31159467 | 22.545 | 0.0863 | 0.0033 | 3.7496 | 2.2158E-24 | SIG |
| 5 | GW | MC | 1 | 504208472 | 5 | 4208472 | 14.5038 | -0.0633 | 0.0018 | 2.0327 | 3.02037E-16 | SIG |
| 6 | GW | MC | 1 | 505361276 | 5 | 5361276 | 9.2478 | -0.0479 | 0.0016 | 1.797 | 6.76449E-11 | SIG |
| 7 | GW | MC | 1 | 508320511 | 5 | 8320511 | 18.5035 | -0.0746 | 0.0039 | 4.4281 | 2.68469E-20 | SIG |
| 8 | GW | MC | 1 | 809925342 | 8 | 9925342 | 29.3035 | -0.1086 | 0.0025 | 2.8668 | 4.98371E-30 | SIG |
| 9 | GW | MC | 1 | 810329229 | 8 | 10329229 | 27.2041 | 0.1037 | 0.0083 | 9.429 | 4.43075E-29 | SIG |
| 10 | GW | MC | 1 | 1011713473 | 10 | 11713473 | 5.1586 | -0.0348 | 0.0007 | 0.7523 | 1.09391E-06 | SUG |
| 11 | GW | MC | 1 | 1117167095 | 11 | 17167095 | 6.5878 | -0.0403 | 0.0016 | 1.773 | 2.58506E-07 | SUG |
| 12 | GW | MC | 1 | 1125125749 | 11 | 25125749 | 11.754 | -0.055 | 0.0019 | 2.1948 | 1.87937E-13 | SIG |
| 13 | GW | MC | 1 | 1206317244 | 12 | 6317244 | 3.8527 | 0.0293 | 0.0008 | 0.938 | 2.53034E-05 | SUG |
| 14 | GW | MC | 2 | 140476043 | 1 | 40476043 | 19.1758 | 0.0682 | 0.0046 | 5.0728 | 6.68261E-20 | SIG |
| 15 | GW | MC | 2 | 201165732 | 2 | 1165732 | 21.8807 | 0.0754 | 0.0019 | 2.0622 | 1.0379E-23 | SIG |
| 16 | GW | MC | 2 | 314763587 | 3 | 14763587 | 6.5998 | 0.0355 | 0.0012 | 1.3254 | 3.52939E-08 | SUG |
| 17 | GW | MC | 2 | 326844557 | 3 | 26844557 | 9.8773 | -0.0443 | 0.0019 | 2.0867 | 1.53817E-11 | SIG |
| 18 | GW | MC | 2 | 420400208 | 4 | 20400208 | 23.5701 | -0.0799 | 0.0013 | 1.4429 | 2.04668E-25 | SIG |
| 19 | GW | MC | 2 | 423538685 | 4 | 23538685 | 5.6833 | -0.0342 | 0.0011 | 1.1622 | 2.0743E-06 | SUG |
| 20 | GW | MC | 2 | 504775669 | 5 | 4775669 | 13.8669 | -0.0359 | 0.0028 | 3.0877 | 1.36036E-14 | SIG |
| 21 | GW | MC | 2 | 505358771 | 5 | 5358771 | 28.7431 | -0.0942 | 0.0063 | 6.965 | 1.81099E-29 | SIG |
| 22 | GW | MC | 2 | 701743812 | 7 | 1743812 | 10.6038 | -0.0468 | 0.001 | 1.1555 | 2.79042E-12 | SIG |
| 23 | GW | MC | 2 | 907776660 | 9 | 7776660 | 15.3839 | 0.0586 | 0.0008 | 0.8978 | 3.86871E-17 | SIG |
| 24 | GW | MC | 2 | 910486075 | 9 | 10486075 | 12.5618 | 0.0518 | 0.0025 | 2.7227 | 2.83301E-14 | SIG |
| 25 | GW | MC | 2 | 1116995448 | 11 | 16995448 | 16.1202 | -0.0608 | 0.0013 | 1.4708 | 7.59262E-17 | SIG |
| 26 | GW | MC | 2 | 1125261662 | 11 | 25261662 | 11.3025 | 0.048 | 0.0023 | 2.5276 | 5.41563E-13 | SIG |
| 27 | GW | MC | MEJA | 128170409 | 1 | 28170409 | 13.8449 | 0.0309 | 0.001 | 1.0742 | 1.40857E-15 | SIG |
| 28 | GW | MC | MEJA | 134815173 | 1 | 34815173 | 26.8606 | -0.0457 | 0.0006 | 0.7069 | 9.83432E-29 | SIG |
| 29 | GW | MC | MEJA | 140476043 | 1 | 40476043 | 26.7642 | 0.0446 | 0.002 | 2.3036 | 1.72514E-27 | SIG |
| 30 | GW | MC | MEJA | 201236423 | 2 | 1236423 | 38.7668 | 0.0585 | 0.0012 | 1.3784 | 1.01932E-40 | SIG |
| 31 | GW | MC | MEJA | 219799308 | 2 | 19799308 | 4.2375 | 0.016 | 0.0002 | 0.2201 | 5.79031E-05 | SUG |
| 32 | GW | MC | MEJA | 220138937 | 2 | 20138937 | 5.8795 | 0.019 | 0.0004 | 0.4028 | 1.95689E-07 | SUG |
| 33 | GW | MC | MEJA | 313813715 | 3 | 13813715 | 27.7314 | -0.0465 | 0.0013 | 1.4914 | 1.30341E-29 | SIG |
| 34 | GW | MC | MEJA | 322032925 | 3 | 22032925 | 7.6972 | -0.022 | 0.0001 | 0.1687 | 2.00967E-08 | SIG |
| 35 | GW | MC | MEJA | 323970533 | 3 | 23970533 | 26.8433 | -0.0438 | 0.0003 | 0.3897 | 1.43792E-27 | SIG |
| 36 | GW | MC | MEJA | 504631781 | 5 | 4631781 | 55.9965 | -0.0784 | 0.0041 | 4.5799 | 5.014E-58 | SIG |
| 37 | GW | MC | MEJA | 505466023 | 5 | 5466023 | 12.6484 | 0.0294 | 0.0008 | 0.9537 | 2.31334E-14 | SIG |
| 38 | GW | MC | MEJA | 508182309 | 5 | 8182309 | 24.942 | -0.0434 | 0.0015 | 1.7054 | 8.45378E-27 | SIG |
| 39 | GW | MC | MEJA | 600023671 | 6 | 23671 | 16.6264 | -0.0365 | 0.0011 | 1.1848 | 2.36693E-17 | SIG |
| 40 | GW | MC | MEJA | 810329229 | 8 | 10329229 | 38.9909 | 0.0607 | 0.0028 | 3.1942 | 6.06745E-41 | SIG |
| 41 | GW | MC | MEJA | 816385094 | 8 | 16385094 | 18.7139 | -0.0375 | 0.0005 | 0.5269 | 1.93549E-19 | SIG |
| 42 | GW | MC | MEJA | 826515715 | 8 | 26515715 | 10.0799 | 0.0255 | 0.0006 | 0.7217 | 9.55458E-12 | SIG |
| 43 | GW | MC | MEJA | 910486075 | 9 | 10486075 | 15.7431 | 0.033 | 0.001 | 1.1299 | 1.67276E-17 | SIG |
| 44 | GW | MC | MEJA | 1101221125 | 11 | 1221125 | 37.1215 | 0.0582 | 0.001 | 1.1747 | 7.58364E-38 | SIG |
| 45 | GW | MC | MEJA | 1117149260 | 11 | 17149260 | 25.2208 | -0.0462 | 0.0018 | 2.0181 | 6.02797E-26 | SIG |
| 46 | GW | MC | MEJA | 1125624085 | 11 | 25624085 | 15.9484 | 0.033 | 0.0011 | 1.2256 | 1.03608E-17 | SIG |
| 47 | GW | MC | MEJA | 1211178871 | 12 | 11178871 | 19.2252 | 0.0381 | 0.001 | 1.1307 | 5.96297E-20 | SIG |
| 48 | GL | MC | 1 | 316708508 | 3 | 16708508 | 35.051 | 0.3596 | 0.108 | 15.1157 | 5.56673E-37 | SIG |
| 49 | GL | MC | 1 | 413796107 | 4 | 13796107 | 8.1622 | 0.1266 | 0.0067 | 0.9347 | 8.74248E-10 | SIG |
| 50 | GL | MC | 1 | 433360398 | 4 | 33360398 | 19.534 | -0.2229 | 0.0221 | 3.0868 | 2.4376E-21 | SIG |
| 51 | GL | MC | 1 | 500585798 | 5 | 585798 | 16.7948 | 0.1992 | 0.0164 | 2.2968 | 1.4393E-18 | SIG |
| 52 | GL | MC | 1 | 519329552 | 5 | 19329552 | 19.2694 | 0.2197 | 0.0144 | 2.0116 | 4.51322E-21 | SIG |
| 53 | GL | MC | 1 | 525364698 | 5 | 25364698 | 28.4998 | -0.2999 | 0.0219 | 3.0595 | 3.17127E-29 | SIG |
| 54 | GL | MC | 1 | 701179170 | 7 | 1179170 | 14.2194 | 0.178 | 0.0303 | 4.2415 | 5.8696E-16 | SIG |
| 55 | GL | MC | 1 | 724533051 | 7 | 24533051 | 14.8238 | -0.1847 | 0.0124 | 1.7313 | 1.43032E-16 | SIG |
| 56 | GL | MC | 1 | 818394489 | 8 | 18394489 | 25.3648 | 0.2711 | 0.0077 | 1.0811 | 3.1674E-27 | SIG |
| 57 | GL | MC | 1 | 906852848 | 9 | 6852848 | 14.6602 | -0.1825 | 0.0139 | 1.9414 | 2.09606E-16 | SIG |
| 58 | GL | MC | 1 | 1015482498 | 10 | 15482498 | 10.5568 | -0.1485 | 0.0074 | 1.0284 | 3.11626E-12 | SIG |
| 59 | GL | MC | 1 | 1204175263 | 12 | 4175263 | 17.9864 | -0.2091 | 0.0305 | 4.2676 | 8.95469E-20 | SIG |
| 60 | GL | MC | 1 | 1214201832 | 12 | 14201832 | 9.58 | -0.1419 | 0.0178 | 2.4925 | 3.09532E-11 | SIG |
| 61 | GL | MC | 2 | 106259714 | 1 | 6259714 | 5.1544 | 0.0836 | 0.0067 | 0.9781 | 1.10496E-06 | SUG |
| 62 | GL | MC | 2 | 206081603 | 2 | 6081603 | 7.1025 | -0.1002 | 0.0092 | 1.339 | 1.07153E-08 | SIG |
| 63 | GL | MC | 2 | 316708508 | 3 | 16708508 | 34.822 | 0.3082 | 0.0794 | 11.6067 | 9.46292E-37 | SIG |
| 64 | GL | MC | 2 | 401015786 | 4 | 1015786 | 10.6329 | -0.1275 | 0.0072 | 1.0478 | 2.60619E-12 | SIG |
| 65 | GL | MC | 2 | 431203233 | 4 | 31203233 | 8.5668 | 0.1124 | 0.0117 | 1.7086 | 3.36564E-10 | SIG |
| 66 | GL | MC | 2 | 525364698 | 5 | 25364698 | 23.0667 | -0.2161 | 0.0111 | 1.6253 | 8.59262E-24 | SIG |
| 67 | GL | MC | 2 | 621530176 | 6 | 21530176 | 18.4514 | -0.1879 | 0.0121 | 1.7642 | 3.03109E-20 | SIG |
| 68 | GL | MC | 2 | 702288888 | 7 | 2288888 | 12.979 | -0.1332 | 0.0175 | 2.5539 | 1.05079E-13 | SIG |
| 69 | GL | MC | 2 | 712384603 | 7 | 12384603 | 16.1546 | -0.169 | 0.0059 | 0.8623 | 7.01407E-17 | SIG |
| 70 | GL | MC | 2 | 818394489 | 8 | 18394489 | 40.2764 | 0.3558 | 0.0133 | 1.9465 | 3.09455E-42 | SIG |
| 71 | GL | MC | 2 | 1114067932 | 11 | 14067932 | 11.6549 | -0.0696 | 0.0189 | 2.7665 | 2.21572E-12 | SIG |
| 72 | GL | MC | 2 | 1204146868 | 12 | 4146868 | 14.5841 | -0.1583 | 0.0157 | 2.2946 | 2.50342E-16 | SIG |
| 73 | GL | MC | 2 | 1218051198 | 12 | 18051198 | 13.7088 | -0.1499 | 0.0099 | 1.4476 | 1.9362E-15 | SIG |
| 74 | GL | MC | 2 | 1222906272 | 12 | 22906272 | 8.7552 | -0.1138 | 0.0123 | 1.8041 | 2.1586E-10 | SIG |
| 75 | GL | MC | 2 | 1223901098 | 12 | 23901098 | 15.6252 | 0.1635 | 0.0202 | 2.9499 | 2.20247E-17 | SIG |
| 76 | GL | MC | MEJA | 203464709 | 2 | 3464709 | 36.8937 | -0.1542 | 0.0034 | 0.4872 | 7.79729E-39 | SIG |
| 77 | GL | MC | MEJA | 316717631 | 3 | 16717631 | 52.5223 | 0.1995 | 0.0332 | 4.7681 | 3.01712E-53 | SIG |
| 78 | GL | MC | MEJA | 316866114 | 3 | 16866114 | 29.3891 | 0.1337 | 0.0163 | 2.3419 | 2.78644E-31 | SIG |
| 79 | GL | MC | MEJA | 413501788 | 4 | 13501788 | 11.8661 | 0.0749 | 0.0026 | 0.3771 | 1.44502E-13 | SIG |
| 80 | GL | MC | MEJA | 431203233 | 4 | 31203233 | 8.0842 | 0.0609 | 0.0034 | 0.4928 | 1.051E-09 | SIG |
| 81 | GL | MC | MEJA | 433360398 | 4 | 33360398 | 57.0338 | -0.2144 | 0.0204 | 2.934 | 4.55993E-59 | SIG |
| 82 | GL | MC | MEJA | 500585798 | 5 | 585798 | 22.611 | 0.1097 | 0.005 | 0.7153 | 1.90072E-24 | SIG |
| 83 | GL | MC | MEJA | 519329552 | 5 | 19329552 | 40.9191 | 0.164 | 0.008 | 1.1514 | 6.99E-43 | SIG |
| 84 | GL | MC | MEJA | 525364698 | 5 | 25364698 | 72.0289 | -0.2646 | 0.0166 | 2.3857 | 9.41315E-73 | SIG |
| 85 | GL | MC | MEJA | 601969823 | 6 | 1969823 | 18.2664 | -0.094 | 0.0092 | 1.3179 | 5.42342E-19 | SIG |
| 86 | GL | MC | MEJA | 624698553 | 6 | 24698553 | 46.8305 | 0.1826 | 0.009 | 1.2915 | 8.02202E-49 | SIG |
| 87 | GL | MC | MEJA | 702288888 | 7 | 2288888 | 17.8087 | -0.0958 | 0.0077 | 1.1047 | 1.55592E-18 | SIG |
| 88 | GL | MC | MEJA | 716562571 | 7 | 16562571 | 19.8383 | -0.109 | 0.0025 | 0.3634 | 1.45342E-20 | SIG |
| 89 | GL | MC | MEJA | 724533051 | 7 | 24533051 | 62.4943 | -0.2333 | 0.0198 | 2.8369 | 1.50965E-64 | SIG |
| 90 | GL | MC | MEJA | 818394489 | 8 | 18394489 | 78.5299 | 0.2872 | 0.0087 | 1.246 | 1.24342E-80 | SIG |
| 91 | GL | MC | MEJA | 823153658 | 8 | 23153658 | 6.0102 | -0.0423 | 0.0019 | 0.2685 | 9.77393E-07 | SUG |
| 92 | GL | MC | MEJA | 906852848 | 9 | 6852848 | 30.1805 | -0.1329 | 0.0074 | 1.0579 | 4.44606E-32 | SIG |
| 93 | GL | MC | MEJA | 922540770 | 9 | 22540770 | 8.1595 | 0.0609 | 0.0036 | 0.5205 | 8.79878E-10 | SIG |
| 94 | GL | MC | MEJA | 1117785691 | 11 | 17785691 | 9.2556 | 0.062 | 0.0039 | 0.565 | 5.55522E-10 | SIG |
| 95 | GL | MC | MEJA | 1204415230 | 12 | 4415230 | 26.8737 | -0.1242 | 0.011 | 1.5772 | 9.53918E-29 | SIG |
| 96 | GL | MC | MEJA | 1223901098 | 12 | 23901098 | 9.039 | 0.0644 | 0.0031 | 0.4497 | 1.10589E-10 | SIG |
| 97 | TGW | MC | 1 | 128597255 | 1 | 28597255 | 5.2591 | 0.432 | 0.1503 | 1.6125 | 8.60224E-07 | SUG |
| 98 | TGW | MC | 1 | 201334322 | 2 | 1334322 | 13.6646 | 0.7641 | 0.1914 | 2.0546 | 2.14667E-15 | SIG |
| 99 | TGW | MC | 1 | 231234763 | 2 | 31234763 | 11.6622 | 0.6901 | 0.3594 | 3.8566 | 2.33019E-13 | SIG |
| 100 | TGW | MC | 1 | 308081540 | 3 | 8081540 | 19.2708 | 0.9724 | 0.4715 | 5.0598 | 5.36914E-20 | SIG |
| 101 | TGW | MC | 1 | 334733054 | 3 | 34733054 | 18.712 | 0.9477 | 0.3715 | 3.9867 | 1.65208E-20 | SIG |
| 102 | TGW | MC | 1 | 421023194 | 4 | 21023194 | 23.3256 | -1.1227 | 0.7788 | 8.3576 | 3.61135E-25 | SIG |
| 103 | TGW | MC | 1 | 520615822 | 5 | 20615822 | 16.0957 | -0.8565 | 0.2852 | 3.0609 | 7.34876E-18 | SIG |
| 104 | TGW | MC | 1 | 701080125 | 7 | 1080125 | 8.0755 | -0.5647 | 0.2711 | 2.9096 | 8.40927E-09 | SIG |
| 105 | TGW | MC | 1 | 801493649 | 8 | 1493649 | 10.9137 | 0.136 | 0.4496 | 4.8254 | 1.22092E-11 | SIG |
| 106 | TGW | MC | 1 | 808787015 | 8 | 8787015 | 17.4512 | -0.9229 | 0.5215 | 5.5971 | 3.11629E-19 | SIG |
| 107 | TGW | MC | 1 | 1013414896 | 10 | 13414896 | 35.3806 | -1.647 | 0.5869 | 6.298 | 4.17526E-36 | SIG |
| 108 | TGW | MC | 1 | 1020319057 | 10 | 20319057 | 18.0652 | 0.9502 | 0.2247 | 2.4117 | 7.4522E-20 | SIG |
| 109 | TGW | MC | 1 | 1211816442 | 12 | 11816442 | 9.948 | 0.6644 | 0.2866 | 3.0761 | 1.12806E-10 | SIG |
| 110 | TGW | MC | 2 | 139719661 | 1 | 39719661 | 6.7908 | -0.5558 | 0.1478 | 1.5768 | 2.24317E-08 | SIG |
| 111 | TGW | MC | 2 | 201334322 | 2 | 1334322 | 14.0614 | 0.8553 | 0.2398 | 2.5594 | 8.49046E-16 | SIG |
| 112 | TGW | MC | 2 | 231332457 | 2 | 31332457 | 24.4393 | 1.2897 | 1.253 | 13.3715 | 3.64444E-25 | SIG |
| 113 | TGW | MC | 2 | 316786485 | 3 | 16786485 | 12.7899 | 0.804 | 0.476 | 5.0791 | 1.66112E-14 | SIG |
| 114 | TGW | MC | 2 | 401274245 | 4 | 1274245 | 12.2406 | -0.7808 | 0.2203 | 2.3505 | 5.75217E-13 | SIG |
| 115 | TGW | MC | 2 | 421456460 | 4 | 21456460 | 19.0632 | 1.055 | 0.2631 | 2.8073 | 7.29336E-21 | SIG |
| 116 | TGW | MC | 2 | 505651540 | 5 | 5651540 | 9.4813 | 0.6763 | 0.4518 | 4.821 | 3.90419E-11 | SIG |
| 117 | TGW | MC | 2 | 506624426 | 5 | 6624426 | 11.0132 | -0.738 | 0.3264 | 3.4832 | 1.06756E-12 | SIG |
| 118 | TGW | MC | 2 | 619661779 | 6 | 19661779 | 12.0594 | -0.7814 | 0.497 | 5.304 | 9.18676E-14 | SIG |
| 119 | TGW | MC | 2 | 715650325 | 7 | 15650325 | 13.6998 | 0.8484 | 0.6078 | 6.4856 | 1.97717E-15 | SIG |
| 120 | TGW | MC | 2 | 1022657739 | 10 | 22657739 | 6.8293 | 0.0828 | 0.2664 | 2.8429 | 1.48242E-07 | SUG |
| 121 | TGW | MC | 2 | 1218171070 | 12 | 18171070 | 7.713 | -0.5901 | 0.3482 | 3.716 | 2.52646E-09 | SIG |
| 122 | TGW | MC | MEJA | 104440399 | 1 | 4440399 | 45.5274 | 0.8301 | 0.1629 | 1.7501 | 1.63458E-47 | SIG |
| 123 | TGW | MC | MEJA | 201334322 | 2 | 1334322 | 26.694 | 0.5696 | 0.1064 | 1.1431 | 1.44773E-28 | SIG |
| 124 | TGW | MC | MEJA | 231332457 | 2 | 31332457 | 69.2022 | 1.1786 | 1.0523 | 11.3093 | 6.31417E-70 | SIG |
| 125 | TGW | MC | MEJA | 304386976 | 3 | 4386976 | 7.8796 | 0.0366 | 0.0728 | 0.7822 | 1.32028E-08 | SIG |
| 126 | TGW | MC | MEJA | 316030163 | 3 | 16030163 | 21.5744 | 0.5165 | 0.0975 | 1.0479 | 2.66904E-22 | SIG |
| 127 | TGW | MC | MEJA | 335486847 | 3 | 35486847 | 44.6591 | -0.8216 | 0.3528 | 3.7913 | 1.21864E-46 | SIG |
| 128 | TGW | MC | MEJA | 606509590 | 6 | 6509590 | 16.9334 | 0.4296 | 0.1827 | 1.9639 | 1.04191E-18 | SIG |
| 129 | TGW | MC | MEJA | 624595315 | 6 | 24595315 | 48.206 | 0.8808 | 0.2321 | 2.4949 | 6.24927E-49 | SIG |
| 130 | TGW | MC | MEJA | 701027206 | 7 | 1027206 | 3.2423 | -0.1735 | 0.0281 | 0.3016 | 0.000572569 | SUG |
| 131 | TGW | MC | MEJA | 718006816 | 7 | 18006816 | 16.0732 | 0.0504 | 0.1706 | 1.8332 | 8.46026E-17 | SIG |
| 132 | TGW | MC | MEJA | 800779283 | 8 | 779283 | 21.8836 | 0.5186 | 0.1539 | 1.6543 | 1.03095E-23 | SIG |
| 133 | TGW | MC | MEJA | 808787015 | 8 | 8787015 | 16.1063 | -0.4268 | 0.1115 | 1.1987 | 7.16844E-18 | SIG |
| 134 | TGW | MC | MEJA | 811983096 | 8 | 11983096 | 19.8284 | -0.4835 | 0.0651 | 0.6994 | 1.48696E-20 | SIG |
| 135 | TGW | MC | MEJA | 902228113 | 9 | 2228113 | 17.8491 | -0.4523 | 0.1003 | 1.078 | 1.41768E-18 | SIG |
| 136 | TGW | MC | MEJA | 912157401 | 9 | 12157401 | 8.4664 | 0.291 | 0.0798 | 0.8571 | 3.4188E-09 | SIG |
| 137 | TGW | MC | MEJA | 1013414896 | 10 | 13414896 | 50.6161 | -0.9334 | 0.1963 | 2.1101 | 2.43095E-51 | SIG |
| 138 | TGW | MC | MEJA | 1013915559 | 10 | 13915559 | 11.209 | -0.3659 | 0.0518 | 0.5564 | 6.18662E-12 | SIG |
| 139 | TGW | MC | MEJA | 1016066492 | 10 | 16066492 | 62.0748 | -1.0775 | 0.3509 | 3.7714 | 8.46272E-63 | SIG |
| 140 | TGW | MC | MEJA | 1020319057 | 10 | 20319057 | 25.3428 | 0.5662 | 0.0798 | 0.8576 | 3.33347E-27 | SIG |
| 141 | TGW | MC | MEJA | 1105109969 | 11 | 5109969 | 26.6095 | 0.6006 | 0.105 | 1.1285 | 2.46289E-27 | SIG |
| 142 | TGW | MC | MEJA | 1117579299 | 11 | 17579299 | 17.0636 | 0.4311 | 0.1716 | 1.8447 | 8.65123E-18 | SIG |
| 143 | GW | RR | 1 | 331660510 | 3 | 31660510 | 11.4522 | -0.0453 | 0.0019 | 2.9624 | 3.8131E-13 | SIG |
| 144 | GW | RR | 1 | 334749626 | 3 | 34749626 | 23.6811 | -0.0773 | 0.0015 | 2.3682 | 2.0881E-24 | SIG |
| 145 | GW | RR | 1 | 504887394 | 5 | 4887394 | 11.7447 | -0.0458 | 0.0019 | 2.8804 | 1.92081E-13 | SIG |
| 146 | GW | RR | 1 | 505357438 | 5 | 5357438 | 10.2018 | -0.0421 | 0.0012 | 1.8724 | 7.17371E-12 | SIG |
| 147 | GW | RR | 1 | 508237047 | 5 | 8237047 | 10.0911 | -0.042 | 0.0013 | 1.9853 | 9.30603E-12 | SIG |
| 148 | GW | RR | 1 | 514400275 | 5 | 14400275 | 13.3007 | -0.0498 | 0.0006 | 0.9141 | 5.02752E-15 | SIG |
| 149 | GW | RR | 1 | 615329899 | 6 | 15329899 | 12.6148 | -0.0518 | 0.001 | 1.5509 | 2.43055E-13 | SIG |
| 150 | GW | RR | 1 | 805990045 | 8 | 5990045 | 10.5446 | -0.044 | 0.0015 | 2.2954 | 2.85617E-11 | SIG |
| 151 | GW | RR | 1 | 809892381 | 8 | 9892381 | 14.9327 | -0.0541 | 0.0007 | 1.0819 | 1.16919E-15 | SIG |
| 152 | GW | RR | 1 | 906961682 | 9 | 6961682 | 16.898 | 0.0582 | 0.0014 | 2.1686 | 1.13144E-18 | SIG |
| 153 | GW | RR | 1 | 1100505957 | 11 | 505957 | 17.5536 | 0.06 | 0.0016 | 2.5041 | 2.79954E-18 | SIG |
| 154 | GW | RR | 1 | 1117167095 | 11 | 17167095 | 6.2798 | -0.0326 | 0.001 | 1.5764 | 5.25298E-07 | SUG |
| 155 | GW | RR | 1 | 1126344986 | 11 | 26344986 | 13.1399 | 0.0496 | 0.0024 | 3.7709 | 7.32358E-15 | SIG |
| 156 | GW | RR | 1 | 1206235646 | 12 | 6235646 | 7.2258 | 0.0349 | 0.0012 | 1.8462 | 8.00164E-09 | SIG |
| 157 | GW | RR | 1 | 1210323387 | 12 | 10323387 | 9.746 | 0.0411 | 0.0015 | 2.2485 | 2.09434E-11 | SIG |
| 158 | GW | RR | 2 | 213494629 | 2 | 13494629 | 7.816 | 0.0096 | 0.0009 | 1.4383 | 1.52849E-08 | SIG |
| 159 | GW | RR | 2 | 223888162 | 2 | 23888162 | 19.3163 | -0.0545 | 0.0011 | 1.649 | 4.04556E-21 | SIG |
| 160 | GW | RR | 2 | 327019634 | 3 | 27019634 | 5.4418 | 0.0253 | 0.0006 | 0.8758 | 3.61745E-06 | SUG |
| 161 | GW | RR | 2 | 331294993 | 3 | 31294993 | 7.3088 | 0.031 | 0.0009 | 1.3494 | 4.91428E-08 | SUG |
| 162 | GW | RR | 2 | 334717822 | 3 | 34717822 | 33.3841 | -0.0852 | 0.0024 | 3.6908 | 2.64801E-35 | SIG |
| 163 | GW | RR | 2 | 414029946 | 4 | 14029946 | 16.5921 | -0.0497 | 0.0015 | 2.3092 | 2.30891E-18 | SIG |
| 164 | GW | RR | 2 | 504890865 | 5 | 4890865 | 14.8471 | -0.0455 | 0.0018 | 2.8467 | 1.42364E-15 | SIG |
| 165 | GW | RR | 2 | 506015422 | 5 | 6015422 | 6.6755 | -0.0286 | 0.0008 | 1.2346 | 2.94935E-08 | SUG |
| 166 | GW | RR | 2 | 514043668 | 5 | 14043668 | 8.7305 | 0.0328 | 0.0011 | 1.6527 | 1.8612E-09 | SIG |
| 167 | GW | RR | 2 | 808322454 | 8 | 8322454 | 7.9285 | 0.0299 | 0.001 | 1.5451 | 1.1797E-08 | SIG |
| 168 | GW | RR | 2 | 907794065 | 9 | 7794065 | 12.7174 | 0.0403 | 0.0008 | 1.1787 | 1.91906E-13 | SIG |
| 169 | GW | RR | 2 | 1100462294 | 11 | 462294 | 21.4543 | 0.059 | 0.0014 | 2.2312 | 2.79757E-23 | SIG |
| 170 | GW | RR | 2 | 1107626245 | 11 | 7626245 | 33.709 | -0.0859 | 0.0015 | 2.3439 | 1.24709E-35 | SIG |
| 171 | GW | RR | 2 | 1119233702 | 11 | 19233702 | 19.7632 | -0.056 | 0.0006 | 1.0036 | 1.728E-20 | SIG |
| 172 | GW | RR | 2 | 1126041008 | 11 | 26041008 | 21.4017 | 0.0591 | 0.0035 | 5.4163 | 3.16148E-23 | SIG |
| 173 | GW | RR | 2 | 1206958480 | 12 | 6958480 | 9.3359 | 0.0346 | 0.0011 | 1.6932 | 4.61764E-10 | SIG |
| 174 | GW | RR | 2 | 1212942520 | 12 | 12942520 | 18.72 | 0.054 | 0.0015 | 2.3457 | 1.90842E-19 | SIG |
| 175 | GW | RR | MEJA | 128170409 | 1 | 28170409 | 7.0988 | 0.0239 | 0.0006 | 0.8736 | 1.081E-08 | SIG |
| 176 | GW | RR | MEJA | 128963821 | 1 | 28963821 | 11.0072 | -0.0299 | 0.0003 | 0.4967 | 1.08279E-12 | SIG |
| 177 | GW | RR | MEJA | 331660510 | 3 | 31660510 | 11.5507 | -0.0308 | 0.0009 | 1.362 | 3.02644E-13 | SIG |
| 178 | GW | RR | MEJA | 334749626 | 3 | 34749626 | 55.6746 | -0.0901 | 0.0018 | 2.7522 | 2.12546E-56 | SIG |
| 179 | GW | RR | MEJA | 413817255 | 4 | 13817255 | 19.574 | -0.0418 | 0.001 | 1.5399 | 2.22087E-21 | SIG |
| 180 | GW | RR | MEJA | 504890865 | 5 | 4890865 | 36.9838 | -0.0641 | 0.0036 | 5.5412 | 1.04116E-37 | SIG |
| 181 | GW | RR | MEJA | 514043673 | 5 | 14043673 | 6.9961 | 0.0233 | 0.0005 | 0.8399 | 1.00959E-07 | SUG |
| 182 | GW | RR | MEJA | 519309079 | 5 | 19309079 | 32.2117 | -0.0574 | 0.0007 | 1.0365 | 4.00764E-34 | SIG |
| 183 | GW | RR | MEJA | 806428588 | 8 | 6428588 | 7.6109 | -0.0009 | 0.0006 | 0.8482 | 2.45136E-08 | SIG |
| 184 | GW | RR | MEJA | 808422704 | 8 | 8422704 | 4.4671 | -0.0184 | 0.0003 | 0.4863 | 3.41224E-05 | SUG |
| 185 | GW | RR | MEJA | 906272138 | 9 | 6272138 | 22.6644 | -0.0455 | 0.0006 | 0.9652 | 2.1701E-23 | SIG |
| 186 | GW | RR | MEJA | 907177752 | 9 | 7177752 | 17.2254 | 0.0388 | 0.0007 | 1.0184 | 5.95923E-18 | SIG |
| 187 | GW | RR | MEJA | 910486075 | 9 | 10486075 | 4.2651 | 0.0181 | 0.0003 | 0.4615 | 9.34471E-06 | SUG |
| 188 | GW | RR | MEJA | 1013144659 | 10 | 13144659 | 9.3313 | 0.0276 | 0.0003 | 0.5281 | 5.55654E-11 | SIG |
| 189 | GW | RR | MEJA | 1100462294 | 11 | 462294 | 30.0347 | 0.0547 | 0.0012 | 1.9062 | 6.23571E-32 | SIG |
| 190 | GW | RR | MEJA | 1119009026 | 11 | 19009026 | 44.0179 | -0.0722 | 0.0014 | 2.2014 | 9.63192E-45 | SIG |
| 191 | GW | RR | MEJA | 1125735889 | 11 | 25735889 | 15.8163 | -0.0372 | 0.0008 | 1.2337 | 1.41029E-17 | SIG |
| 192 | GW | RR | MEJA | 1126041008 | 11 | 26041008 | 21.1015 | 0.0438 | 0.0019 | 2.9502 | 6.35395E-23 | SIG |
| 193 | GW | RR | MEJA | 1205779427 | 12 | 5779427 | 13.4468 | -0.0334 | 0.0011 | 1.6748 | 3.57221E-15 | SIG |
| 194 | GW | RR | MEJA | 1227465140 | 12 | 27465140 | 36.6599 | 0.0646 | 0.0013 | 2.0147 | 2.19487E-37 | SIG |
| 195 | GL | RR | 1 | 105286322 | 1 | 5286322 | 11.0077 | -0.1247 | 0.0125 | 1.9952 | 1.08146E-12 | SIG |
| 196 | GL | RR | 1 | 130551892 | 1 | 30551892 | 10.0797 | 0.1197 | 0.0035 | 0.5539 | 9.55748E-12 | SIG |
| 197 | GL | RR | 1 | 203464709 | 2 | 3464709 | 34.6122 | -0.2973 | 0.0126 | 2.0111 | 1.53861E-36 | SIG |
| 198 | GL | RR | 1 | 211127824 | 2 | 11127824 | 10.9385 | 0.1247 | 0.0077 | 1.2336 | 1.27204E-12 | SIG |
| 199 | GL | RR | 1 | 218536168 | 2 | 18536168 | 20.9306 | -0.1927 | 0.0099 | 1.584 | 9.45657E-23 | SIG |
| 200 | GL | RR | 1 | 220073320 | 2 | 20073320 | 6.5252 | -0.0857 | 0.0072 | 1.1451 | 2.98585E-07 | SUG |
| 201 | GL | RR | 1 | 316708508 | 3 | 16708508 | 32.8677 | 0.2795 | 0.0653 | 10.412 | 8.76352E-35 | SIG |
| 202 | GL | RR | 1 | 325129163 | 3 | 25129163 | 8.0181 | 0.1039 | 0.0098 | 1.5663 | 9.59892E-09 | SIG |
| 203 | GL | RR | 1 | 405647187 | 4 | 5647187 | 10.1194 | -0.1182 | 0.0038 | 0.6092 | 7.60293E-11 | SIG |
| 204 | GL | RR | 1 | 602130119 | 6 | 2130119 | 13.6394 | 0.1436 | 0.0164 | 2.6083 | 2.27726E-15 | SIG |
| 205 | GL | RR | 1 | 604799710 | 6 | 4799710 | 4.5567 | -0.0748 | 0.0044 | 0.7029 | 4.63185E-06 | SUG |
| 206 | GL | RR | 1 | 616448073 | 6 | 16448073 | 11.5144 | -0.0089 | 0.015 | 2.3891 | 3.06202E-12 | SIG |
| 207 | GL | RR | 1 | 616645152 | 6 | 16645152 | 15.1696 | -0.1548 | 0.0234 | 3.728 | 6.37904E-17 | SIG |
| 208 | GL | RR | 1 | 724964429 | 7 | 24964429 | 11.2229 | -0.1268 | 0.0048 | 0.7698 | 6.52746E-13 | SIG |
| 209 | GL | RR | 1 | 1117942058 | 11 | 17942058 | 7.2966 | -0.0997 | 0.0099 | 1.5799 | 6.76696E-09 | SIG |
| 210 | GL | RR | 2 | 105307303 | 1 | 5307303 | 8.6829 | -0.1114 | 0.0102 | 1.5652 | 2.55936E-10 | SIG |
| 211 | GL | RR | 2 | 106382838 | 1 | 6382838 | 8.4984 | -0.1099 | 0.0119 | 1.8251 | 3.95419E-10 | SIG |
| 212 | GL | RR | 2 | 316708508 | 3 | 16708508 | 30.0472 | 0.2662 | 0.0592 | 9.1082 | 6.05662E-32 | SIG |
| 213 | GL | RR | 2 | 323679384 | 3 | 23679384 | 18.7845 | 0.1845 | 0.0081 | 1.2481 | 1.39552E-20 | SIG |
| 214 | GL | RR | 2 | 334087282 | 3 | 34087282 | 19.3813 | 0.1879 | 0.0072 | 1.1105 | 3.47825E-21 | SIG |
| 215 | GL | RR | 2 | 404758399 | 4 | 4758399 | 32.5564 | -0.286 | 0.0141 | 2.1642 | 1.80279E-34 | SIG |
| 216 | GL | RR | 2 | 420737161 | 4 | 20737161 | 13.4944 | 0.1465 | 0.0057 | 0.8828 | 3.19574E-15 | SIG |
| 217 | GL | RR | 2 | 422021281 | 4 | 22021281 | 10.8665 | 0.1285 | 0.0155 | 2.3894 | 1.36112E-11 | SIG |
| 218 | GL | RR | 2 | 602000963 | 6 | 2000963 | 10.5265 | 0.1269 | 0.0126 | 1.9447 | 3.34596E-12 | SIG |
| 219 | GL | RR | 2 | 627016141 | 6 | 27016141 | 13.6259 | -0.1481 | 0.0103 | 1.5888 | 2.35004E-15 | SIG |
| 220 | GL | RR | 2 | 702255614 | 7 | 2255614 | 10.5597 | -0.1271 | 0.0161 | 2.4814 | 3.09492E-12 | SIG |
| 221 | GL | RR | 2 | 725124146 | 7 | 25124146 | 32.1281 | -0.2851 | 0.0195 | 3.0058 | 4.86461E-34 | SIG |
| 222 | GL | RR | 2 | 820715449 | 8 | 20715449 | 9.7133 | -0.1243 | 0.0105 | 1.6108 | 1.93653E-10 | SIG |
| 223 | GL | RR | 2 | 1116841934 | 11 | 16841934 | 8.7542 | -0.1193 | 0.0103 | 1.5856 | 1.7626E-09 | SIG |
| 224 | GL | RR | 2 | 1222906272 | 12 | 22906272 | 5.604 | -0.0869 | 0.0072 | 1.1063 | 3.77406E-07 | SUG |
| 225 | GL | RR | MEJA | 105286322 | 1 | 5286322 | 19.8025 | -0.1052 | 0.0089 | 1.3989 | 1.30473E-21 | SIG |
| 226 | GL | RR | MEJA | 131330968 | 1 | 31330968 | 26.8628 | -0.1351 | 0.0042 | 0.666 | 1.37455E-27 | SIG |
| 227 | GL | RR | MEJA | 316708508 | 3 | 16708508 | 78.1825 | 0.2975 | 0.074 | 11.6104 | 2.77318E-80 | SIG |
| 228 | GL | RR | MEJA | 334087282 | 3 | 34087282 | 36.3241 | 0.1565 | 0.005 | 0.7866 | 2.91665E-38 | SIG |
| 229 | GL | RR | MEJA | 406883844 | 4 | 6883844 | 20.9996 | -0.1106 | 0.0045 | 0.7044 | 1.00264E-21 | SIG |
| 230 | GL | RR | MEJA | 514607988 | 5 | 14607988 | 19.425 | -0.104 | 0.0035 | 0.5565 | 3.14169E-21 | SIG |
| 231 | GL | RR | MEJA | 602130119 | 6 | 2130119 | 24.7884 | 0.1216 | 0.0117 | 1.841 | 1.20787E-26 | SIG |
| 232 | GL | RR | MEJA | 606777283 | 6 | 6777283 | 17.7358 | 0.0984 | 0.0088 | 1.3866 | 1.60542E-19 | SIG |
| 233 | GL | RR | MEJA | 611986480 | 6 | 11986480 | 5.4758 | -0.0512 | 0.0015 | 0.2431 | 5.12568E-07 | SUG |
| 234 | GL | RR | MEJA | 616291330 | 6 | 16291330 | 10.6426 | -0.0744 | 0.0054 | 0.8493 | 2.54781E-12 | SIG |
| 235 | GL | RR | MEJA | 616496495 | 6 | 16496495 | 23.581 | -0.1222 | 0.0079 | 1.2334 | 2.62937E-24 | SIG |
| 236 | GL | RR | MEJA | 625506895 | 6 | 25506895 | 6.1836 | -0.0546 | 0.0024 | 0.3842 | 9.48995E-08 | SUG |
| 237 | GL | RR | MEJA | 714471382 | 7 | 14471382 | 23.272 | -0.1229 | 0.0054 | 0.8477 | 5.35622E-24 | SIG |
| 238 | GL | RR | MEJA | 724964429 | 7 | 24964429 | 28.4517 | -0.133 | 0.0053 | 0.8342 | 2.45096E-30 | SIG |
| 239 | GL | RR | MEJA | 728370748 | 7 | 28370748 | 8.0007 | -0.0627 | 0.0016 | 0.2554 | 1.28025E-09 | SIG |
| 240 | GL | RR | MEJA | 818538960 | 8 | 18538960 | 29.7184 | 0.1363 | 0.0055 | 0.8669 | 1.91739E-30 | SIG |
| 241 | GL | RR | MEJA | 820715449 | 8 | 20715449 | 25.8924 | -0.1315 | 0.0117 | 1.8438 | 1.28399E-26 | SIG |
| 242 | GL | RR | MEJA | 1117749520 | 11 | 17749520 | 9.648 | 0.0701 | 0.0046 | 0.7233 | 2.25077E-10 | SIG |
| 243 | GL | RR | MEJA | 1220922577 | 12 | 20922577 | 13.8147 | -0.0788 | 0.0069 | 1.083 | 1.5339E-14 | SIG |
| 244 | GL | RR | MEJA | 1224378744 | 12 | 24378744 | 53.7492 | 0.2113 | 0.0106 | 1.6567 | 9.03984E-56 | SIG |
| 245 | TGW | RR | 1 | 116843929 | 1 | 16843929 | 3.5053 | -0.4602 | 0.1991 | 1.9767 | 5.8775E-05 | SUG |
| 246 | TGW | RR | 1 | 225050031 | 2 | 25050031 | 17.5623 | -1.1933 | 0.3907 | 3.8793 | 2.40511E-19 | SIG |
| 247 | TGW | RR | 1 | 231234763 | 2 | 31234763 | 16.5073 | 1.1273 | 0.9589 | 9.521 | 2.8138E-18 | SIG |
| 248 | TGW | RR | 1 | 316860759 | 3 | 16860759 | 14.9949 | 1.0559 | 0.5502 | 5.4634 | 9.59182E-17 | SIG |
| 249 | TGW | RR | 1 | 330465188 | 3 | 30465188 | 18.262 | -1.2156 | 0.3522 | 3.4974 | 4.71174E-20 | SIG |
| 250 | TGW | RR | 1 | 334603105 | 3 | 34603105 | 6.4123 | 0.6333 | 0.2671 | 2.652 | 5.5101E-08 | SUG |
| 251 | TGW | RR | 1 | 404708103 | 4 | 4708103 | 11.4124 | -0.9139 | 0.4568 | 4.5361 | 4.18555E-13 | SIG |
| 252 | TGW | RR | 1 | 909860435 | 9 | 9860435 | 16.0162 | 1.1092 | 0.3697 | 3.6709 | 8.84509E-18 | SIG |
| 253 | TGW | RR | 1 | 1000463104 | 10 | 463104 | 12.6179 | -0.9918 | 0.273 | 2.7111 | 2.41328E-13 | SIG |
| 254 | TGW | RR | 1 | 1124975065 | 11 | 24975065 | 10.696 | 0.8496 | 0.4429 | 4.3975 | 2.2476E-12 | SIG |
| 255 | TGW | RR | 1 | 1212726597 | 12 | 12726597 | 6.2594 | -0.6247 | 0.3626 | 3.6003 | 7.92465E-08 | SUG |
| 256 | TGW | RR | 1 | 1218076754 | 12 | 18076754 | 5.2973 | -0.5822 | 0.2606 | 2.5881 | 7.85124E-07 | SUG |
| 257 | TGW | RR | 2 | 136231866 | 1 | 36231866 | 15.8551 | 1.1778 | 0.5859 | 6.5847 | 1.39807E-16 | SIG |
| 258 | TGW | RR | 2 | 316746142 | 3 | 16746142 | 8.6665 | 0.7924 | 0.5153 | 5.7912 | 2.66021E-10 | SIG |
| 259 | TGW | RR | 2 | 321432053 | 3 | 21432053 | 6.9048 | 0.689 | 0.3114 | 3.5001 | 1.24593E-07 | SUG |
| 260 | TGW | RR | 2 | 330923484 | 3 | 30923484 | 15.8324 | 1.1387 | 1.3302 | 14.949 | 1.47308E-16 | SIG |
| 261 | TGW | RR | 2 | 912157401 | 9 | 12157401 | 7.0546 | 0.6978 | 0.4698 | 5.28 | 8.82355E-08 | SUG |
| 262 | TGW | RR | 2 | 1113147262 | 11 | 13147262 | 8.5139 | -0.8343 | 0.1799 | 2.0215 | 3.065E-09 | SIG |
| 263 | TGW | RR | 2 | 1114219838 | 11 | 14219838 | 7.9381 | 0.7553 | 0.552 | 6.2033 | 1.15395E-08 | SIG |
| 264 | TGW | RR | 2 | 1122488893 | 11 | 22488893 | 10.0497 | 0.864 | 0.4302 | 4.8348 | 8.92527E-11 | SIG |
| 265 | TGW | RR | 2 | 1125403331 | 11 | 25403331 | 19.0333 | 1.321 | 0.6731 | 7.5642 | 7.81912E-21 | SIG |
| 266 | TGW | RR | 2 | 1226496493 | 12 | 26496493 | 10.4215 | 0.8943 | 0.6892 | 7.7453 | 3.79191E-11 | SIG |
| 267 | TGW | RR | MEJA | 130178104 | 1 | 30178104 | 24.3908 | 0.8067 | 0.1551 | 1.625 | 3.04119E-26 | SIG |
| 268 | TGW | RR | MEJA | 135730043 | 1 | 35730043 | 8.215 | -0.3776 | 0.1548 | 1.6215 | 6.09993E-09 | SIG |
| 269 | TGW | RR | MEJA | 209639175 | 2 | 9639175 | 11.8507 | 0.5346 | 0.2222 | 2.3277 | 1.49817E-13 | SIG |
| 270 | TGW | RR | MEJA | 220073320 | 2 | 20073320 | 14.1549 | -0.5867 | 0.2965 | 3.1067 | 7.00853E-15 | SIG |
| 271 | TGW | RR | MEJA | 234221194 | 2 | 34221194 | 23.2131 | -0.7783 | 0.1805 | 1.8912 | 4.69056E-25 | SIG |
| 272 | TGW | RR | MEJA | 316746142 | 3 | 16746142 | 15.3296 | 0.6055 | 0.3009 | 3.1521 | 4.39123E-17 | SIG |
| 273 | TGW | RR | MEJA | 330465188 | 3 | 30465188 | 19.7028 | -0.7063 | 0.1189 | 1.2457 | 1.64554E-21 | SIG |
| 274 | TGW | RR | MEJA | 334603105 | 3 | 34603105 | 7.6097 | 0.4129 | 0.1136 | 1.1896 | 3.22553E-09 | SIG |
| 275 | TGW | RR | MEJA | 335460643 | 3 | 35460643 | 24.1383 | -0.8017 | 0.3379 | 3.5401 | 7.28844E-25 | SIG |
| 276 | TGW | RR | MEJA | 615164468 | 6 | 15164468 | 13.1059 | -0.5467 | 0.3059 | 3.2054 | 7.84491E-14 | SIG |
| 277 | TGW | RR | MEJA | 809047476 | 8 | 9047476 | 8.1283 | -0.4299 | 0.0981 | 1.0274 | 9.47197E-10 | SIG |
| 278 | TGW | RR | MEJA | 909834183 | 9 | 9834183 | 32.1967 | 0.9645 | 0.3051 | 3.196 | 4.14947E-34 | SIG |
| 279 | TGW | RR | MEJA | 919802752 | 9 | 19802752 | 7.3308 | -0.4028 | 0.1449 | 1.5181 | 6.24072E-09 | SIG |
| 280 | TGW | RR | MEJA | 1003974680 | 10 | 3974680 | 10.5848 | 0.5107 | 0.2424 | 2.5395 | 2.60382E-11 | SIG |
| 281 | TGW | RR | MEJA | 1116327428 | 11 | 16327428 | 14.4138 | 0.5828 | 0.3349 | 3.5083 | 3.86163E-15 | SIG |
| 282 | TGW | RR | MEJA | 1117803729 | 11 | 17803729 | 7.907 | 0.4176 | 0.1572 | 1.6465 | 1.23966E-08 | SIG |
| 283 | TGW | RR | MEJA | 1125987286 | 11 | 25987286 | 32.6882 | 0.9835 | 0.3224 | 3.378 | 1.32826E-34 | SIG |

GW: grain width; GL: grain length; TGW: thousand grain weight; MC: main crop; RR: ratoon rice; MEJA: multi-environment joint analysis

**Table S25 QTN-by-environment interactions for grain width, grain length, and thousand grain weight in main crop and ratoon rice of 159 rice accessions using evolutionary population**

| **No.** | **Trait** | **MC or RR** | **Marker** | **Chromosome** | **Position (bp)** | **LOD (QE)** | **add*env1** | **add*env2** | **variance** | **r2(%)** | **P-value** | **significance** |
| --- | --- | --- | --- | --- | --- | --- | --- | --- | --- | --- | --- | --- |
| 1 | TGW | MC | 1221081445 | 12 | 21081445 | 22.7628 | -0.5975 | 0.5975 | 0.324 | 3.4818 | 1.72988E-23 | SIG |
| 2 | GW | RR | 207733574 | 2 | 7733574 | 11.5029 | -0.0246 | 0.0246 | 0.0006 | 0.7436 | 3.38529E-13 | SIG |
| 3 | GW | RR | 1000710130 | 10 | 710130 | 6.7857 | -0.0186 | 0.0186 | 0.0003 | 0.4236 | 2.27061E-08 | SIG |
| 4 | GL | RR | 420414980 | 4 | 20414980 | 20.7638 | -0.0765 | 0.0765 | 0.0058 | 0.9097 | 1.39379E-22 | SIG |
| 5 | GL | RR | 626752211 | 6 | 26752211 | 23.6848 | 0.0818 | -0.0818 | 0.0068 | 1.0544 | 2.07062E-24 | SIG |
| 6 | TGW | RR | 419524638 | 4 | 19524638 | 6.503 | -0.3098 | 0.3098 | 0.0966 | 1.0919 | 3.14239E-07 | SUG |
| 7 | TGW | RR | 516578452 | 5 | 16578452 | 21.4266 | -0.606 | 0.606 | 0.3673 | 4.1535 | 2.98334E-23 | SIG |
| 8 | TGW | RR | 715307889 | 7 | 15307889 | 9.2854 | -0.3787 | 0.3787 | 0.1434 | 1.6221 | 6.19102E-11 | SIG |
| 9 | TGW | RR | 1116753283 | 11 | 16753283 | 17.0483 | 0.5317 | -0.5317 | 0.2827 | 3.197 | 7.97074E-19 | SIG |

GW: grain width; GL: grain length; TGW: thousand grain weight; MC: main crop; RR: ratoon rice

**Table S26 QTN-by-environment interactions for grain width, grain length, and thousand grain weight in main crop and ratoon rice of 159 rice accessions using Q matrix**

| **Trait** | **MC or RR** | **Marker** | **Chromosome** | **Position (bp)** | **LOD (QE)** | **add*env1** | **add*env2** | **variance** | **r2(%)** | **P-value** | **significance** |
| --- | --- | --- | --- | --- | --- | --- | --- | --- | --- | --- | --- |
| TGW | MC | 420947566 | 4 | 20947566 | 4.5597 | -0.2086 | 0.2086 | 0.0435 | 0.4675 | 4.60E-06 | SUG |
| TGW | MC | 805244100 | 8 | 5244100 | 5.1678 | -0.2228 | 0.2228 | 0.0496 | 0.5333 | 1.07E-06 | SUG |
| TGW | MC | 1221081445 | 12 | 21081445 | 20.3266 | -0.5096 | 0.5096 | 0.2364 | 2.5403 | 4.72E-21 | SIG |
| TGW | MC | 1223044143 | 12 | 23044143 | 5.1663 | 0.2237 | -0.2237 | 0.0501 | 0.538 | 1.07E-06 | SUG |
| TGW | RR | 505434037 | 5 | 5434037 | 9.473 | -0.4632 | 0.4632 | 0.2146 | 2.2479 | 3.98E-11 | SIG |
| TGW | RR | 516648952 | 5 | 16648952 | 64.3049 | -1.6589 | 1.6589 | 2.788 | 29.21 | 4.98E-65 | SIG |

GW: grain width; GL: grain length; TGW: thousand grain weight; MC: main crop; RR: ratoon rice

**Table S27 Known genes around main-effect QTNs and QEIs of rice grain size in main crop and ratoon rice using evolutionary population structure**

| **Trait** | **MC/RR** | **Chr** | **Posi (bp)** | **LOD scores of Q and QEI detection in different datasets** | | | | **R2 (%)** | **Known genes** | **Reference** |
| --- | --- | --- | --- | --- | --- | --- | --- | --- | --- | --- |
|  |  |  |  | **I** | | **II** | **I+II** |  |  |  |
| GW | Both | 5 | 5357438~5361276 | 28.69~20.2645 | 16.6238~17.4274 | | 52.7153~41.1839 | 2.0292~10.2534 | *GW5* | Weng et al., 2008 |
|  | Both | 5 | 6015422~6052088 |  | 11.791 | | 9.3278 | 0.8856~2.6493 | *JMJ703* | Cui et al., 2013 |
|  | MC | 8 | 26714889 | 12.7967 |  | | 42.6112 | 1.0379~1.4184 | *WTG1* | Huang et al., 2017 |
| GL | Both | 3 | 16708508~16746142 | 26.1652~33.5357 | 35.1278~46.204 | | 88.325~119.44 | 6.0501~17.7195 | *GS3* | Mao et al., 2010 |
|  | MC | 3 | 25129163 |  |  | | 22.5203 | 1.8712 | *GL3.1* | Qi et al., 2012 |
|  | RR | 5 | 5413770~5914985 |  | 14.3552 | | 48.789 | 0.9154~1.7693 | *GW5* | Weng et al., 2008 |
|  | RR | 6 | 1623846 |  | 8.2691 | |  | 1.5926 | *OsACS6* | Matsushima et al., 2016 |
|  | Both | 7 | 24533051 | 19.2738 |  | | 43.7233 | 0.8801~3.178 | *GW7* | Wang et al., 2015 |
| TGW | RR | 2 | 31102971 | 10.8632 |  | |  | 3.4486 | *OsNF-YB1* | Xu et al., 2016 |
|  | MC | 3 | 35437797~35486847 | 15.9056 | 8.5083 | | 12.3697 | 0.866~4.5181 | *qTGW3* | Ying et al., 2018 |
|  | RR | 4 | 4570606 | 14.2645 |  | | 21.6974 | 1.5592~2.5515 | *ETR2* | Wuriyanghan et al., 2009 |

GW: grain width; GL: grain length; TGW: thousand grain weight; MC: main crop; RR: ratoon rice

**Table S28 Known genes around main-effect QTNs and QEIs of rice grain size in main crop and ratoon rice using Q matrix**

| **Trait** | **MC/RR** | **Chr** | **Posi (bp)** | **LOD scores of Q and QEI detection in different datasets** | | | **R2 (%)** | **Known genes** | **Reference** |
| --- | --- | --- | --- | --- | --- | --- | --- | --- | --- |
|  |  |  |  | I | II | I+II |  |  |  |
| GW | MC | 3 | 13813715~14763587 |  | 6.5998 | 27.7314 | 1.3254~1.4914 | *VLN2* | Wu et al., 2015 |
|  | MC | 4 | 23538685 |  | 5.6833 |  | 1.1622 | *D11* | Zhu et al., 2015 |
|  | Both | 5 | 5357438~5466023 | 9.2478~10.2018 | 28.7431 | 12.6484 | 0.9537~6.9650 | *GW5* | Weng et al., 2008 |
|  | RR | 5 | 6015422 |  | 6.6755 |  | 1.2346 | *JMJ703* | Cui et al., 2013 |
| GL | Both | 3 | 16708508~16717631 | 32.8677~35.051 | 30.0472~34.822 | 52.5223~78.1825 | 4.7681~15.1157 | *GS3* | Mao et al., 2010 |
|  | RR | 3 | 25129163 | 8.0181 |  |  | 1.5663 | *GL3.1* | Qi et al., 2012 |
|  | MC | 7 | 24533051 | 14.8238 |  | 62.4943 | 1.7313~2.8369 | *GW7* | Wang et al., 2015 |
|  | MC | 3 | 4386976 |  |  | 7.8796 | 0.7822 | *OsLG3* | Yu et al., 2017 |
|  | Both | 3 | 35486847~35460643 |  |  | 24.1383~44.6591 | 3.5401~3.7913 | *qTGW3* | Ying et al., 2018 |
|  | RR | 4 | 4708103 | 11.4124 |  |  | 4.5361 | *ETR2* | Wuriyanghan et al., 2009 |
|  | Both | 5 | 5434037 |  |  | 9.473 ^QE^ | 2.2479 | *GW5* | Weng et al., 2008 |
|  | MC | 12 | 23044143 |  |  | 5.1663^QE^ | 0.538 | *OsSar1b* | Tian et al., 2013 |

GW: grain width; GL: grain length; TGW: thousand grain weight; MC: main crop; RR: ratoon rice

**Reference:**

1. Cui, X., Jin, P., Cui, X., Gu, L., Lu, Z., Xue, Y., et al. (2013). Control of transposon activity by a histone H3K4 demethylase in rice. *Proc. Natl. Acad. Sci.* *U. S. A*. 110, 1953–1958. [doi: 10.1073/pnas.1217020110](https://doi.org/10.1073/pnas.1217020110)
2. Huang, K., Wang, D., Duan, P., Zhang, B., Xu, R., Li, N., et al. (2017). WIDE AND THICK GRAIN 1, which encodes an otubain-like protease with deubiquitination activity, influences grain size and shape in rice. *Plant J*. 91, 849–860. [doi: 10.1111/tpj.13613](https://doi.org/10.1111/tpj.13613)
3. Mao, H., Sun, S., Yao, J., Wang, C., Yu, S., Xu, C., et al. (2010). Linking differential domain functions of the *GS3* protein to natural variation of grain size in rice. *Proc. Natl. Acad. Sci. U. S. A.* *107*, 19579–19584. doi: 10.1073/pnas.1014419107
4. Matsushima, R., Maekawa, M., Kusano, M., Tomita, K., Kondo, H., Nishimura, H., et al. (2016). Amyloplast membrane protein SUBSTANDARD STARCH grain6 controls starch grain size in rice endosperm. *Plant Physiol*. 170, 1445–1459. [doi: 10.1104/pp.15.01811](https://doi.org/10.1104/pp.15.01811)
5. Qi, P., Lin, Y. S., Song, X. J., Shen, J. B., Huang, W., Shan, J. X., et al. (2012). The novel quantitative trait locus *GL3.1* controls rice grain size and yield by regulating *Cyclin-T1;3*. *Cell Res.* 22, 1666–1680. doi: 10.1038/cr.2012.151
6. Tian, L., Dai, L. L., Yin, Z. J., Fukuda, M., Kumamaru, T., Dong, X. B., et al. (2013). Small GTPase Sar1 is crucial for proglutelin and α-globulin export from the endoplasmic reticulum in rice endosperm. *J. Exp. Bot*. 64, 2831–2845. doi: 10.1093/jxb/ert128
7. Wang, S., Li, S., Liu, Q., Wu, K., Zhang, J., Wang, S., et al. (2015). The *OsSPL16-GW7* regulatory module determines grain shape and simultaneously improves rice yield and grain quality. *Nat. Genet.* 47, 949–954. doi: 10.1038/ng.3352
8. Weng, J., Gu, S., Wan, X., Gao, H., Guo, T., Su, N., et al. (2008). Isolation and initial characterization of *GW5*, a major QTL associated with rice grain width and weight. *Cell Res*. 18, 1199–1209. [doi: 10.1038/cr.2008.307](https://doi.org/10.1038/cr.2008.307)
9. Wu, S., Xie, Y., Zhang, J., Ren, Y., Zhang, X., Wang, J., et al. (2015). *VLN2* regulates plant architecture by affecting microfilament dynamics and polar auxin transport in rice. *Plant Cell*. *27*, 2829–2845. doi: 10.1105/tpc.15.00581
10. Wuriyanghan, H., Zhang, B., Cao, W. H., Ma, B., Lei, G., Liu, Y. F., et al. (2009). The ethylene receptor *ETR2* delays floral transition and affects starch accumulation in rice. *Plant Cell*. 21, 1473-1494. doi: 10.1105/tpc.108.065391
11. Xu, J. J., Zhang, X. F., and Xue, H. W. (2016). Rice aleurone layer specific *OsNF-YB1* regulates grain filling and endosperm development by interacting with an ERF transcription factor. *J. Exp. Bot.* 67, 6399–6411. doi: 10.1093/jxb/erw409
12. Ying, J. Z., Ma, M., Bai, C., Huang, X. H., Liu, J. L., Fan, Y. Y., et al. (2018). *TGW3*, a major QTL that negatively modulates grain length and weight in rice. *Mol. Plant* 11, 750–753. doi: 10.1016/j.molp.2018.03.007
13. Yu, J., Xiong, H., Zhu, X., Zhang, H., Li, H., Miao, J., et al. (2017). *OsLG3* contributing to rice grain length and yield was mined by Ho-LAMap. *BMC biology*. 15, 28. doi: 10.1186/s12915-017-0365-7
14. Zhu, X., Liang, W., Cui, X., Chen, M., Yin, C., Luo, Z., et al. (2015). Brassinosteroids promote development of rice pollen grains and seeds by triggering expression of Carbon Starved Anther, a MYB domain protein. *Plant J*. 82, 570–581. [doi: 10.1111/tpj.12820](https://doi.org/10.1111/tpj.12820)

**Table S29 Main-effect QTNs interactions and QTN-by-environment interactions for grain width when the main crop and ratoon rice datasets were jointly analyzed in environment 1 via 3VmrMLM**

| Main effect QTNs for grain width | | | | | |  | QTN-by-environment interactions for grain width | | | | | | |
| --- | --- | --- | --- | --- | --- | --- | --- | --- | --- | --- | --- | --- | --- |
| No. | Chr | Position (bp) | LOD (Q) | add | r^2^(%) |  | No. | Chr | Position (bp) | LOD (QE) | add*env1 | add*env2 | r^2^(%) |
| ***Significant QTNs at the critical P-value of 2.48e-08 (=0.05/m, where m is the number of markers)*** | | | | | |  | ***Significant QTNs at the critical P-value of 2.48e-08 (=0.05/m, where m is the number of markers)*** | | | | | | |
|  |  |  |  |  |  |  |  |  |  |  |  |  |  |
| 1 | 2 | 1236423 | 13.9233 | 0.0376 | 0.6609 |  | 1 | 5 | 18761512 | 9.1794 | -0.0297 | 0.0297 | 1.1592 |
| 2 | 3 | 26622488 | 11.9991 | -0.0353 | 1.2717 |  |  |  |  |  |  |  |  |
| 3 | 3 | 31660510 | 22.1448 | -0.0498 | 3.0409 |  |  |  |  |  |  |  |  |
| 4 | 3 | 33307806 | 10.8549 | -0.0326 | 0.4576 |  |  |  |  |  |  |  |  |
| 5 | 4 | 21031164 | 36.4387 | -0.069 | 2.4074 |  |  |  |  |  |  |  |  |
| 6 | 5 | 5361276 | 33.008 | -0.065 | 3.8003 |  |  |  |  |  |  |  |  |
| 7 | 5 | 14043668 | 20.7247 | 0.0485 | 2.9809 |  |  |  |  |  |  |  |  |
| 8 | 7 | 3821846 | 14.983 | -0.039 | 0.5499 |  |  |  |  |  |  |  |  |
| 9 | 7 | 7640833 | 9.7088 | -0.0307 | 0.6701 |  |  |  |  |  |  |  |  |
| 10 | 8 | 5990045 | 18.1193 | -0.045 | 2.097 |  |  |  |  |  |  |  |  |
| 11 | 8 | 9925342 | 17.7994 | -0.0432 | 0.8156 |  |  |  |  |  |  |  |  |
| 12 | 8 | 10329229 | 14.9132 | 0.0404 | 1.6442 |  |  |  |  |  |  |  |  |
| 13 | 11 | 1239118 | 40.6084 | 0.0779 | 1.4798 |  |  |  |  |  |  |  |  |
| 14 | 11 | 7833619 | 9.9521 | -0.0313 | 1.0156 |  |  |  |  |  |  |  |  |
| 15 | 11 | 26344986 | 35.0984 | 0.0678 | 5.9665 |  |  |  |  |  |  |  |  |
| 16 | 12 | 6951278 | 8.938 | 0.0274 | 1.0193 |  |  |  |  |  |  |  |  |
| 17 | 12 | 11103678 | 22.6023 | 0.0505 | 3.0877 |  |  |  |  |  |  |  |  |
| ***Suggested QTNs with the LOD score ≥ 3.0 but the P-value > 0.05/m, where m is the number of markers*** | | | | | |  | ***Suggested QTNs with the LOD score ≥ 3.0 but the P-value > 0.05/m, where m is the number of markers*** | | | | | | |
|  |  |  |  |  |  |  | 1 | 11 | 6601331 | 5.7735 | 0.0234 | -0.0234 | 0.716 |

**Table S30 Main-effect QTNs interactions and QTN-by-environment interactions for grain width when the main crop and ratoon rice datasets were jointly analyzed in environment 2 via 3VmrMLM**

| Main effect QTNs for grain width | | | | | |  | QTN-by-environment interactions for grain width | | | | | | |
| --- | --- | --- | --- | --- | --- | --- | --- | --- | --- | --- | --- | --- | --- |
| No. | Chr | Position (bp) | LOD (Q) | add | r^2^(%) |  | No. | Chr | Position (bp) | LOD (QE) | add*env1 | add*env2 | r^2^(%) |
| ***Significant QTNs at the critical P-value of 2.48e-08 (=0.05/m, where m is the number of markers)*** | | | | | |  | ***Significant QTNs at the critical P-value of 2.48e-08 (=0.05/m, where m is the number of markers)*** | | | | | | |
|  |  |  |  |  |  |  |  |  |  |  |  |  |  |
| 1 | 1 | 28963821 | 23.4791 | -0.0488 | 1.1115 |  | 1 | 2 | 3127611 | 10.8653 | -0.0308 | 0.0308 | 1.2327 |
| 2 | 2 | 1186658 | 23.333 | 0.0486 | 1.0127 |  | 2 | 7 | 497250 | 20.6014 | -0.0448 | 0.0448 | 2.5999 |
| 3 | 3 | 26661478 | 7.6167 | 0.0253 | 0.7601 |  | 3 | 7 | 6720541 | 8.0963 | 0.0264 | -0.0264 | 0.8998 |
| 4 | 3 | 35144023 | 9.7719 | 0.0285 | 1.1014 |  |  |  |  |  |  |  |  |
| 5 | 5 | 4631781 | 40.5099 | -0.0707 | 4.2871 |  |  |  |  |  |  |  |  |
| 6 | 5 | 4775669 | 17.4243 | -0.0412 | 1.9806 |  |  |  |  |  |  |  |  |
| 7 | 5 | 5358771 | 14.8852 | -0.0358 | 1.232 |  |  |  |  |  |  |  |  |
| 8 | 5 | 14043668 | 7.683 | 0.0256 | 0.849 |  |  |  |  |  |  |  |  |
| 9 | 8 | 8422704 | 24.1714 | -0.0496 | 2.9834 |  |  |  |  |  |  |  |  |
| 10 | 9 | 6986114 | 14.1681 | 0.0365 | 0.7945 |  |  |  |  |  |  |  |  |
| 11 | 11 | 25125749 | 23.2151 | -0.0483 | 1.9175 |  |  |  |  |  |  |  |  |
| 12 | 11 | 26344986 | 8.0167 | 0.0263 | 0.8893 |  |  |  |  |  |  |  |  |
| 13 | 12 | 10942857 | 16.2536 | 0.0388 | 1.6618 |  |  |  |  |  |  |  |  |
| 14 | 12 | 11690509 | 22.9219 | -0.0497 | 1.2527 |  |  |  |  |  |  |  |  |
| 15 | 12 | 27465140 | 33.6745 | 0.0633 | 1.6138 |  |  |  |  |  |  |  |  |
| ***Suggested QTNs with the LOD score ≥ 3.0 but the P-value > 0.05/m, where m is the number of markers*** | | | | | |  | ***Suggested QTNs with the LOD score ≥ 3.0 but the P-value > 0.05/m, where m is the number of markers*** | | | | | | |
| 1 | 6 | 23061482 | 5.4834 | -0.0216 | 0.3692 |  | 1 | 5 | 4781561 | 4.8934 | 0.0205 | 0.0205 | 0.5455 |
| 2 | 8 | 28419017 | 6.7614 | 0.0266 | 0.5783 |  | 2 | 10 | 11641409 | 5.2518 | -0.0208 | -0.0208 | 0.5612 |

**Table S31 Main-effect QTNs interactions and QTN-by-environment interactions for grain length when the main crop and ratoon rice datasets were jointly analyzed in environment 1 via 3VmrMLM**

| **No.** | **Marker** | **Chr** | **Position (bp)** | **LOD score** | **Additive** | **Variance** | **r^2^(%)** | **P-value** | **Significance** |
| --- | --- | --- | --- | --- | --- | --- | --- | --- | --- |
| ***Significant QTNs at the critical P-value of 2.48e-08 (=0.05/m, where m is the number of markers)*** | | | | | | | | | |
| 1 | 105376057 | 1 | 5376057 | 12.4027 | -0.0978 | 0.0077 | 1.086 | 4.1118E-14 | SIG |
| 2 | 203464709 | 2 | 3464709 | 69.6256 | -0.3293 | 0.0155 | 2.1833 | 1.05827E-71 | SIG |
| 3 | 316708508 | 3 | 16708508 | 59.7811 | 0.2829 | 0.0669 | 9.4351 | 7.97198E-62 | SIG |
| 4 | 321162002 | 3 | 21162002 | 10.1926 | 0.0876 | 0.0061 | 0.8539 | 7.3306E-12 | SIG |
| 5 | 323679384 | 3 | 23679384 | 39.8181 | 0.2055 | 0.0101 | 1.4199 | 8.93891E-42 | SIG |
| 6 | 421998574 | 4 | 21998574 | 13.7777 | 0.1038 | 0.0093 | 1.3134 | 1.64783E-15 | SIG |
| 7 | 514607988 | 5 | 14607988 | 13.2037 | -0.1013 | 0.0034 | 0.475 | 6.30836E-15 | SIG |
| 8 | 602130119 | 6 | 2130119 | 9.0506 | 0.0824 | 0.0054 | 0.7601 | 1.07608E-10 | SIG |
| 9 | 605429644 | 6 | 5429644 | 34.5825 | 0.1857 | 0.0124 | 1.752 | 1.64823E-36 | SIG |
| 10 | 616645152 | 6 | 16645152 | 17.686 | -0.1213 | 0.0143 | 2.0245 | 1.80283E-19 | SIG |
| 11 | 702288888 | 7 | 2288888 | 10.8368 | -0.0893 | 0.007 | 0.981 | 1.45739E-11 | SIG |
| 12 | 724964429 | 7 | 24964429 | 21.0042 | -0.134 | 0.0054 | 0.7612 | 7.96852E-23 | SIG |
| 13 | 818394489 | 8 | 18394489 | 39.4415 | 0.2031 | 0.0043 | 0.6124 | 2.13763E-41 | SIG |
| 14 | 823153658 | 8 | 23153658 | 11.8865 | -0.0607 | 0.008 | 1.1321 | 1.30005E-12 | SIG |
| 15 | 908584051 | 9 | 8584051 | 11.4112 | -0.0937 | 0.0087 | 1.2278 | 3.8834E-12 | SIG |
| 16 | 1015482498 | 10 | 15482498 | 32.6897 | -0.1794 | 0.0107 | 1.5139 | 1.32372E-34 | SIG |
| ***Suggested QTNs with the LOD score ≥ 3.0 but the P-value > 0.05/m, where m is the number of markers*** | | | | | | | | | |
| 1 | 231287545 | 2 | 31287545 | 3.7323 | -0.0519 | 0.0027 | 0.3757 | 3.38696E-05 | SUG |
| 2 | 1224137319 | 12 | 24137319 | 5.6547 | -0.0643 | 0.004 | 0.561 | 3.34E-07 | SUG |

**Table S32 Main-effect QTNs interactions and QTN-by-environment interactions for grain length when the main crop and ratoon rice datasets were jointly analyzed in environment 2 via 3VmrMLM**

| Main effect QTNs for grain length | | | | | |  | QTN-by-environment interactions for grain length | | | | | | |
| --- | --- | --- | --- | --- | --- | --- | --- | --- | --- | --- | --- | --- | --- |
| No. | Chr | Position (bp) | LOD (Q) | add | r^2^(%) |  | No. | Chr | Position (bp) | LOD (QE) | add*env1 | add*env2 | r^2^(%) |
| ***Significant QTNs at the critical P-value of 2.48e-08 (=0.05/m, where m is the number of markers)*** | | | | | |  | ***Significant QTNs at the critical P-value of 2.48e-08 (=0.05/m, where m is the number of markers)*** | | | | | | |
|  |  |  |  |  |  |  |  |  |  |  |  |  |  |
| 1 | 1 | 12352948 | 32.9666 | -0.1932 | 1.461 |  |  |  |  |  |  |  |  |
| 2 | 2 | 20073320 | 8.6547 | -0.0927 | 1.0107 |  |  |  |  |  |  |  |  |
| 3 | 3 | 10436032 | 24.4342 | 0.1599 | 1.2469 |  |  |  |  |  |  |  |  |
| 4 | 3 | 16708508 | 70.1377 | 0.3503 | 14.7467 |  |  |  |  |  |  |  |  |
| 5 | 3 | 34087282 | 20.3814 | 0.1402 | 0.5783 |  |  |  |  |  |  |  |  |
| 6 | 4 | 31182803 | 7.1216 | 0.0778 | 0.8447 |  |  |  |  |  |  |  |  |
| 7 | 5 | 25364698 | 34.476 | -0.1984 | 1.3906 |  |  |  |  |  |  |  |  |
| 8 | 6 | 2130119 | 8.6569 | 0.0861 | 0.8462 |  |  |  |  |  |  |  |  |
| 9 | 7 | 2255614 | 12.5942 | -0.1071 | 1.6465 |  |  |  |  |  |  |  |  |
| 10 | 7 | 13252917 | 28.0827 | -0.1706 | 1.4847 |  |  |  |  |  |  |  |  |
| 11 | 12 | 4146868 | 24.3972 | -0.1591 | 2.2795 |  |  |  |  |  |  |  |  |
| 12 | 12 | 8047799 | 15.4009 | -0.1277 | 0.3736 |  |  |  |  |  |  |  |  |
| 13 | 12 | 14683417 | 12.186 | 0.1041 | 0.6929 |  |  |  |  |  |  |  |  |
| 14 | 12 | 22906272 | 12.4488 | -0.1054 | 1.5216 |  |  |  |  |  |  |  |  |
| ***Suggested QTNs with the LOD score ≥ 3.0 but the P-value > 0.05/m, where m is the number of markers*** | | | | | |  | ***Suggested QTNs with the LOD score ≥ 3.0 but the P-value > 0.05/m, where m is the number of markers*** | | | | | | |
| 1 | 6 | 6533545 | 4.3174 | 0.0594 | 0.5038 |  | 1 | 9 | 22674685 | 6.0692 | 0.0711 | -0.0711 | 0.7275 |
| 2 | 8 | 20719085 | 5.3392 | -0.0663 | 0.5171 |  |  |  |  |  |  |  |  |
| 3 | 12 | 12278648 | 4.2123 | 0.0584 | 0.4572 |  |  |  |  |  |  |  |  |

**Table S33 Main-effect QTNs interactions and QTN-by-environment interactions for thousand grain weigth when the MC and RR datasets were jointly analyzed in environment 1 via 3VmrMLM**

| Main effect QTNs for thousand grain weight | | | | | |  | QTN-by-environment interactions for thousand grain weight | | | | | | |
| --- | --- | --- | --- | --- | --- | --- | --- | --- | --- | --- | --- | --- | --- |
| No. | Chr | Position (bp) | LOD (Q) | add | r^2^(%) |  | No. | Chr | Position (bp) | LOD (QE) | add*env1 | add*env2 | r^2^(%) |
| ***Significant QTNs at the critical P-value of 2.48e-08 (=0.05/m, where m is the number of markers)*** | | | | | |  | ***Significant QTNs at the critical P-value of 2.48e-08 (=0.05/m, where m is the number of markers)*** | | | | | | |
|  |  |  |  |  |  |  |  |  |  |  |  |  |  |
| 1 | 1 | 22418320 | 29.6574 | 0.8972 | 1.926 |  | 1 | 1 | 23822271 | 8.0595 | 0.4171 | -0.4171 | 1.6849 |
| 2 | 1 | 30228079 | 24.1189 | 0.7735 | 1.5792 |  | 2 | 3 | 34731922 | 11.2702 | -0.4926 | 0.4926 | 2.3947 |
| 3 | 3 | 35491798 | 18.7588 | -0.6621 | 2.3506 |  | 3 | 4 | 3684764 | 15.4115 | 0.5973 | -0.5973 | 3.5203 |
| 4 | 5 | 5651540 | 15.7353 | 0.6046 | 3.5625 |  | 4 | 4 | 28669807 | 15.2536 | -0.5883 | 0.5883 | 3.415 |
| 5 | 5 | 12385186 | 17.6721 | -0.6524 | 2.8596 |  | 5 | 6 | 2928548 | 13.1896 | -0.5434 | 0.5434 | 2.9139 |
| 6 | 7 | 10299436 | 27.8927 | -0.8497 | 1.2258 |  |  |  |  |  |  |  |  |
| 7 | 10 | 13915559 | 19.5395 | -0.7335 | 2.0254 |  |  |  |  |  |  |  |  |
| 8 | 11 | 17579299 | 16.7535 | 0.6199 | 3.4871 |  |  |  |  |  |  |  |  |
| 9 | 11 | 20935140 | 21.5605 | -0.7309 | 0.9309 |  |  |  |  |  |  |  |  |
| 10 | 11 | 24662073 | 10.5609 | -0.459 | 1.046 |  |  |  |  |  |  |  |  |
| 11 | 12 | 774947 | 36.2048 | 1.024 | 9.7164 |  |  |  |  |  |  |  |  |
| 12 | 12 | 18076754 | 18.4828 | -0.6779 | 3.4865 |  |  |  |  |  |  |  |  |
| 13 | 12 | 20867041 | 8.3727 | 0.4125 | 1.1219 |  |  |  |  |  |  |  |  |
| ***Suggested QTNs with the LOD score ≥ 3.0 but the P-value > 0.05/m, where m is the number of markers*** | | | | | |  | ***Suggested QTNs with the LOD score ≥ 3.0 but the P-value > 0.05/m, where m is the number of markers*** | | | | | | |
| 1 | 2 | 31234763 | 5.9872 | 0.3491 | 0.9072 |  | 1 | 7 | 1176205 | 4.2237 | -0.2905 | 0.2905 | 0.8326 |
| 2 | 2 | 31262967 | 5.9872 | 0.3491 | 0.9072 |  | 2 | 11 | 22930659 | 5.2075 | -0.3302 | 0.3302 | 1.076 |
| 3 | 2 | 31309615 | 5.9872 | 0.3491 | 0.9072 |  |  |  |  |  |  |  |  |

**Table S34 Main-effect QTNs interactions and QTN-by-environment interactions for thousand grain weigth when the MC and RR datasets were jointly analyzed in environment 2 via 3VmrMLM**

| Main effect QTNs for thousand grain weight | | | | | |  | QTN-by-environment interactions for thousand grain weight | | | | | | |
| --- | --- | --- | --- | --- | --- | --- | --- | --- | --- | --- | --- | --- | --- |
| No. | Chr | Position (bp) | LOD (Q) | add | r^2^(%) |  | No. | Chr | Position (bp) | LOD (QE) | add*env1 | add*env2 | r^2^(%) |
| ***Significant QTNs at the critical P-value of 2.48e-08 (=0.05/m, where m is the number of markers)*** | | | | | |  | ***Significant QTNs at the critical P-value of 2.48e-08 (=0.05/m, where m is the number of markers)*** | | | | | | |
|  |  |  |  |  |  |  |  |  |  |  |  |  |  |
| 1 | 1 | 4921429 | 8.477 | 0.1293 | 1.1634 |  | 1 | 1 | 3937469 | 9.9189 | -0.347 | 0.347 | 1.4093 |
| 2 | 1 | 21033494 | 25.4064 | 0.6287 | 0.8667 |  | 2 | 1 | 13571544 | 7.9299 | 0.3309 | -0.3309 | 1.1244 |
| 3 | 1 | 39719661 | 10.0678 | -0.3771 | 0.6525 |  | 3 | 2 | 15185862 | 9.4869 | -0.3591 | 0.3591 | 1.3521 |
| 4 | 2 | 1334322 | 12.7588 | 0.4247 | 0.7072 |  | 4 | 5 | 16535916 | 12.5831 | -0.4228 | 0.4228 | 1.8352 |
| 5 | 2 | 25079124 | 41.8943 | -0.8722 | 1.7083 |  | 5 | 6 | 3041865 | 12.7082 | -0.4251 | 0.4251 | 1.8551 |
| 6 | 3 | 6596936 | 22.0626 | 0.5784 | 0.9586 |  |  |  |  |  |  |  |  |
| 7 | 3 | 13505333 | 12.4442 | 0.429 | 0.7454 |  |  |  |  |  |  |  |  |
| 8 | 3 | 21041714 | 8.4363 | 0.3398 | 1.1776 |  |  |  |  |  |  |  |  |
| 9 | 3 | 34099731 | 11.9966 | 0.4159 | 0.4413 |  |  |  |  |  |  |  |  |
| 10 | 4 | 27688228 | 11.4517 | -0.4028 | 1.6542 |  |  |  |  |  |  |  |  |
| 11 | 4 | 31939665 | 54.8476 | -1.0415 | 3.3633 |  |  |  |  |  |  |  |  |
| 12 | 5 | 6677633 | 10.5732 | -0.3847 | 0.9347 |  |  |  |  |  |  |  |  |
| 13 | 6 | 11905469 | 22.3925 | -0.5928 | 1.1927 |  |  |  |  |  |  |  |  |
| 14 | 8 | 6562776 | 42.518 | 0.9363 | 1.9633 |  |  |  |  |  |  |  |  |
| 15 | 8 | 17687290 | 11.6247 | -0.4098 | 1.7144 |  |  |  |  |  |  |  |  |
| 16 | 8 | 22332745 | 21.9449 | -0.5784 | 0.8899 |  |  |  |  |  |  |  |  |
| 17 | 10 | 6151974 | 10.6351 | 0.3896 | 1.0674 |  |  |  |  |  |  |  |  |
| 18 | 10 | 20319057 | 18.3668 | 0.5305 | 0.7059 |  |  |  |  |  |  |  |  |
| 19 | 11 | 3518389 | 8.0601 | -0.3317 | 0.7092 |  |  |  |  |  |  |  |  |
| 20 | 11 | 16528346 | 8.2893 | -0.3324 | 1.1 |  |  |  |  |  |  |  |  |
| 21 | 11 | 17131649 | 15.5669 | 0.4831 | 0.7597 |  |  |  |  |  |  |  |  |
| 22 | 11 | 18422045 | 17.1965 | -0.5028 | 2.1818 |  |  |  |  |  |  |  |  |
| 23 | 11 | 24346080 | 9.5536 | -0.3631 | 1.3524 |  |  |  |  |  |  |  |  |
| 24 | 12 | 10698465 | 12.6813 | -0.4315 | 1.3214 |  |  |  |  |  |  |  |  |
| ***Suggested QTNs with the LOD score ≥ 3.0 but the P-value > 0.05/m, where m is the number of markers*** | | | | | |  | ***Suggested QTNs with the LOD score ≥ 3.0 but the P-value > 0.05/m, where m is the number of markers*** | | | | | | |
| 1 | 6 | 28648301 | 5.0909 | 0.261 | 0.6777 |  | 1 | 5 | 27885076 | 6.1393 | -0.2893 | 0.2893 | 0.8591 |
| 2 | 12 | 424245 | 4.8449 | -0.224 | 0.6631 |  | 2 | 11 | 19057049 | 6.5466 | 0.2982 | -0.2982 | 0.913 |

**Table S35 Known genes around QTNs that were mined by the main crop and ratoon rice joint analysis in each environment**

| **Trait** | **No.** | **Chr** | **Posi (bp)** | **LOD scores of QTN in two environmets** | | **R^2^ (%)** | **Significance** | **Comparative genomics analysis** | | **Reference** |
| --- | --- | --- | --- | --- | --- | --- | --- | --- | --- | --- |
|  |  |  |  |  |  |  |  |  |  |  |
|  |  |  |  | **I** | **II** |  |  | **Known genes** | **Distance (kb)** |  |
| Grain width | 1 | 5 | 5358771~5361276 | 33.01 | 14.89 | 1.23~3.80 | Significant | *GW5* | 3.846~6.351 | Liu et al., 2017 |
| Grain length | 2 | 2 | 31287545 | 3.73 |  | 0.38 | Significant | *PGL2* | 136.398 | Jang et al., 2017 |
|  | 3 | 3 | 16708508 | 59.78 | 70.14 | 9.44~14.75 | Significant | *GS3* | 20.94 | Mao et al., 2010 |
| Thousand grain weight | 1 | 3 | 35491798 | 18.76 |  | 2.35 | Significant | *qTGW3* | 99.736 | Ying et al., 2018 |

**Reference:**

Jang, S., An, G., and Li, H. Y. (2017). Rice Leaf Angle and Grain Size Are Affected by the *OsBUL1* Transcriptional Activator Complex. *Plant Physiol*. 173, 688–702. [doi: 10.1104/pp.16.01653](https://doi.org/10.1104/pp.16.01653)

Liu, J., Chen, J., Zheng, X., Wu, F., Lin, Q., Heng, Y., et al. (2017). *GW5* acts in the brassinosteroid signalling pathway to regulate grain width and weight in rice. *Nat. Plants* 3, 17043. doi: 10.1038/nplants.2017.43

Mao, H., Sun, S., Yao, J., Wang, C., Yu, S., Xu, C., et al. (2010). Linking differential domain functions of the *GS3* protein to natural variation of grain size in rice. *Proc. Natl. Acad. Sci. U. S. A.* 107, 19579–19584. doi: 10.1073/pnas.1014419107

Ying, J. Z., Ma, M., Bai, C., Huang, X. H., Liu, J. L., Fan, Y. Y., et al. (2018). *TGW3*, a major QTL that negatively modulates grain length and weight in rice. *Mol. Plant* 11, 750–753. doi: 10.1016/j.molp.2018.03.007

**Table S36 Candidate genes around main-effect QTNs that were mined by the main crop and ratoon rice datasets jointly analysis**

| **Trait** | **No.** | **Locus** | | **LOD scores** | **r^2^ (%)** | **Gene differential expression analysis** | | |  | **GO annotation** | | | |  |  |
| --- | --- | --- | --- | --- | --- | --- | --- | --- | --- | --- | --- | --- | --- | --- | --- |
|  |  | **Chr** | **Posi (bp)** |  |  | **Gene_ID** | **log2(Fold Change)** | **P-value** |  | **GO_ID** | **GO_name** | **E-value** | **Reference** |  |  |
| Grain width | 1 | 2 | 1236423 | 13.92 | 0.66 | Os02g0126400 | 1.03 | 2.43E-02 |  | GO:0009738 | abscisic acid-activated signaling pathway | 0 | Qin et al., 2021 |  |  |
|  | 2 | 3 | 35144023 | 9.77 | 1.10 | Os03g0838100 | 1.12 | 4.90E-03 |  | GO:0006468 | protein phosphorylation | 0 | Qiu et al., 2016 |  |  |
|  | 3 | 5 | 27883451 | 7.53 | 0.82 | Os05g0563400 | 1.21 | 3.27E-03 |  | GO:0009850 | auxin metabolic process | 0 | Liu et al., 2015 |  |  |
|  | 4 | 8 | 5990045 | 18.12 | 2.10 | Os08g0200500 | 1.63 | 1.04E-03 |  | GO:0006468 | protein phosphorylation | 1.59E-268 | Qiu et al., 2016 |  |  |
|  | 5 | 9 | 6986114 | 14.17 | 0.79 | Os09g0294300 | -1.6 | 3.46E-03 |  | GO:0016567 | protein ubiquitination | 2.71E-288 | Li et al., 2018 |  |  |
|  | 6 | 11 | 7833619 | 9.95 | 1.02 | Os11g0247000 | -1.87 | 7.41E-03 |  | GO:0009733 | response to auxin | 3.53E-50 | Liu et al., 2015 |  |  |
|  | 7 | 12 | 11103678 | 22.60 | 3.09 | Os12g0288000 | 1.76 | 1.42E-03 |  | GO:0009734 | auxin-activated signaling pathway | 1.27E-264 | Liu et al., 2015 |  |  |
|  | 8 | 12 | 11690509 | 22.92 | 1.25 | Os12g0297500 | 1.2 | 4.35E-02 |  | GO:0006468 | protein phosphorylation | 0 | Qiu et al., 2016 |  |  |
| Grain length | 1 | 1 | 5376057 | 12.40 | 1.09 | Os01g0197100 | 1.04 | 4.50E-03 |  | GO:0016131 | brassinosteroid metabolic process | 0 | Liu et al., 2017 |  |  |
|  | 2 | 2 | 20073320 | 8.65 | 1.01 | Os02g0538000 | 1.23 | 6.74E-03 |  | GO:0009793 | embryo development ending in seed dormancy | 0 | Figueiredo et al., 2014 | |  |
|  | 3 | 5 | 14607988 | 13.20 | 0.48 | Os05g0314700 | 1.11 | 3.26E-02 |  | GO:0031398 | positive regulation of protein ubiquitination | 4.82E-67 | Li et al., 2018 | |  |
|  | 4 | 5 | 25364698 | 34.48 | 1.39 | Os05g0514200 | 1.05 | 0.0173 |  | GO:0006468 | protein phosphorylation | 0 | Qiu et al., 2016 | | |
|  | 5 | 6 | 6533545 | 4.32 | 0.50 | Os06g0226700 | 1.06 | 0.0163 |  | GO:0009735 | response to cytokinin | 7.01E-203 | Jameson et al., 2016 | | |
|  | 6 | 8 | 20719085 | 5.34 | 0.52 | Os08g0428100 | 1.13 | 0.00712 |  | GO:0009793 | embryo development ending in seed dormancy | 2.46E-248 | Figueiredo et al., 2014 | |  |
|  | 7 | 12 | 22906272 | 12.45 | 1.52 | Os12g0557800 | 1.79 | 0.00495 |  | GO:0009737 | response to abscisic acid | 5.65E-216 | Qin et al., 2021 |  |  |
| Thousand grain weight | 1 | 1 | 30228079 | 24.12 | 1.58 | Os01g0724500 | 1.58 | 0.00208 |  | GO:0071365 | cellular response to auxin stimulus | 0 | Liu et al., 2015 |  |  |
|  | 2 | 5 | 6677633 | 10.57 | 0.93 | Os05g0210600 | -1.41 | 0.00625 |  | GO:0009742 | brassinosteroid mediated signaling pathway | 8.64E-316 | Liu et al., 2017 |  |  |
|  | 3 | 8 | 17687290 | 11.62 | 1.71 | Os08g0379300 | -1.18 | 0.0272 |  | GO:0005983 | starch catabolic process | 0 | Zhang et al., 2016 |  |  |
|  | 4 | 8 | 22332745 | 21.94 | 0.89 | Os08g0457600 | 1.04 | 0.00928 |  | GO:0009737 | response to abscisic acid | 0 | Qin et al., 2021 |  |  |
|  | 5 | 11 | 17579299 | 16.75 | 3.49 | Os11g0498600 | -1.12 | 0.0378 |  | GO:0009737 | response to abscisic acid | 1.21E-80 | Qin et al., 2021 |  |  |
|  | 6 | 11 | 24346080 | 9.55 | 1.35 | Os11g0621300 | -1.01 | 0.00979 |  | GO:0010928 | regulation of auxin mediated signaling pathway | 0 | Liu et al., 2015 |  |  |
|  | 7 | 12 | 774947 | 36.20 | 9.72 | Os12g0111500 | 1.81 | 4.64E-04 |  | GO:0006511 | ubiquitin-dependent protein catabolic process | 7.49E-197 | Li et al., 2018 |  |  |

**Reference:**

1. Qin, P., Zhang, G., Hu, B., Wu, J., Chen, W., Ren, Z., et al. (2021). Leaf-derived ABA regulates rice seed development via a transporter-mediated and temperature-sensitive mechanism. *Sci. Adv*. 7, eabc8873. [doi](https://doi): 10.1126/sciadv.abc8873
2. Qiu, J., Hou, Y., Tong, X., Wang, Y., Lin, H., Liu, Q., et al. (2016). Quantitative phosphoproteomic analysis of early seed development in rice (*Oryza sativa* L.). *Plant Mol. Biol*. 90, 249–265. doi: 10.1007/s11103-015-0410-2
3. Liu, L., Tong, H., Xiao, Y., Che, R., Xu, F., Hu, B., et al. (2015). Activation of big grain1 significantly improves grain size by regulating auxin transport in rice. *Proc. Natl. Acad. Sci. U. S. A*. 112, 11102–11107. doi: 10.1073/pnas.1512748112
4. Li, N., Xu, R., Duan, P., and Li, Y. (2018). Control of grain size in rice. *Plant Reprod*. 31, 237–251. doi: 10.1007/s00497-018-0333-6
5. Liu, J., Chen, J., Zheng, X., Wu, F., Lin, Q., Heng, Y., et al. (2017). *GW5* acts in the brassinosteroid signalling pathway to regulate grain width and weight in rice. *Nat. plants* 3, 17043. doi: 10.1038/nplants.2017.43
6. Figueiredo, D. D., and Köhler, C. (2014). Signalling events regulating seed coat development. *Biochem. Soc. Trans*. 42, 358–363. doi: 10.1042/BST20130221
7. Jameson, P. E., and Song, J. (2016). Cytokinin: a key driver of seed yield. *J. Exp. Bot.* 67, 593–606. doi: 10.1093/jxb/erv461
8. Zhang, H., Lu, Y., Zhao, Y., and Zhou, D. X. (2016). *OsSRT1* is involved in rice seed development through regulation of starch metabolism gene expression. *Plant Sci*. 248, 28–36. doi: 10.1016/j.plantsci.2016.04.004

**Table S37 Known genes within 1000 kb upstream and downstream of QTNs and 1500 kb upstream and downstream of QEIs in main crop and ratoon rice**

| **Trait** | **MC/RR** | **No.** | **Chr** | **Posi (bp)** | **LOD scores of QTN and QEI detection in two environments** | | | **R^2^ (%)** | **Comparative genomics analysis** | | **Reference** |
| --- | --- | --- | --- | --- | --- | --- | --- | --- | --- | --- | --- |
|  |  |  |  |  | **I** | **II** | **I + II** |  | **Known genes** | **Distance (kb)** |  |
| GW | RR | 1 | 2 | 7733574 |  |  | 12.92QE | 0.74 | *GW2* | 401.548 | Song et al. 2007 |
|  | RR | 2 | 2 | 25565194 |  | 22.5 |  | 1.13 | *OsVPE3* | 325.251 | Lu et al. 2016 |
|  | RR | 3 | 2 | 29241853~29413021 | 10.82 |  | 11.49 | 0.46~0.78 | *GS2* | 374.856~546.024 | Hu et al. 2015 |
|  | Both | 4 | 3 | 850101~2278621 | 4.42~12.51 |  | 5.88 | 0.26~1.95 | *OsCDPK1* | 618.593~774.997 | Ho et al. 2013 |
|  | RR | 5 | 3 | 3390977 |  |  | 9.59 | 0.2 | *BG1* | 647.364 | Liu et al. 2015 |
|  | Both | 6 | 3 | 13768754~14636351 | 14.58 |  | 11.69~34.25 | 0.44~1.57 | *VLN2* | 8.379~847.666 | Wu et al., 2015 |
|  | MC | 7 | 5 | 636512 |  |  | 14.7 | 0.4 | *GSN1* | 221.887 | Guo et al. 2018 |
|  | Both | 8 | 5 | 5357438~5361276 | 28.87~32.99 | 21.43~44.21 | 55.83~93.69 | 3.75~17.29 | *GW5* | 3.846~7.684 | Liu et al. 2017 |
|  | Both | 9 | 7 | 24208049~25284287 | 24.84 | 18.09~29.02 | 32.31 | 0.49~1.96 | *GW7* | 102.037~614.966 | Wang et al. 2015a |
|  | MC | 10 | 8 | 27378608 |  |  | 66.21 | 0.99 | *OsSPL16* | 872.41 | Wang et al. 2012 |
|  | RR | 11 | 10 | 710130 |  |  | 7.65QE | 0.42 | *OsSCP46* | 599.992 | Li et al. 2016 |
| GL | RR | 1 | 3 | 1294521 |  | 25.37 |  | 1.58 | *OsCDPK1* | 330.577 | Ho et al. 2013 |
|  | Both | 2 | 3 | 16708508~16845802 | 32.72~40.25 | 47.94 | 22.1~36.57 | 2.02~13.09 | *GS3* | 11.033~110.693 | Mao et al. 2010 |
|  | MC | 3 | 3 | 24589241 |  |  | 10.27 | 0.12 | *qGL3* | 453.186 | Zhang et al. 2012 |
|  | MC | 4 | 3 | 35504491 |  | 5.28 |  | 0.68 | *qTGW3* | 112.509 | Ying et al. 2018 |
|  | RR | 5 | 4 | 20228091 |  |  | 15.77QE | 0.74 | *OsACOT* | 146.749 | Zhao et al. 2019 |
|  | Both | 6 | 5 | 5357676~5914985 |  | 10.46 | 33.37~45.93 | 0.73~1.41 | *GW5* | 7.446~548.284 | Liu et al. 2017 |
|  | RR | 7 | 6 | 26752211 |  |  | 22.26QE | 1.03 | *GW6a* | 158.750 | Song et al.2015b |
|  | Both | 8 | 7 | 24206745~24964429 | 10.73~26.22 | 9.07~10.3 | 12.68~67.68 | 0.15~1.52 | *GW7* | 131.277~457.583 | Wang et al. 2015a |
|  | RR | 9 | 9 | 16419362 |  | 16.74 |  | 1.25 | *SG1* | 931.578 | Nakagawa et al. 2012 |
|  | MC | 10 | 11 | 1936516 | 17.84 |  |  | 0.53 | *CycT1;3* | 793.608 | Qi et al. 2012 |
|  | Both | 11 | 12 | 27064916~27144058 | 7.86~8.66 | 8.62 | 40.38 | 0.95~1.68 | *OsPPKL3* | 749.113~828.255 | Zhang et al. 2012 |
| TGW | RR | 1 | 1 | 4921429 |  |  | 8.6 | 1.96 | *smg11* | 315.194 | Fang et al. 2016 |
|  | MC | 2 | 1 | 580218~800544 |  | 5.97 | 17.65 | 0.65~0.88 | *SPL33* | 129.34~349.666 | Wang et al. 2017 |
|  | MC | 3 | 1 | 13552450 |  | 6.85 |  | 1.88 | *OsSar1a* | 264.006 | Tian et al. 2013 |
|  | MC | 4 | 2 | 4346020 |  |  | 22.43 | 0.65 | *OsMADS29* | 508.885 | Nayar et al. 2013 |
|  | MC | 5 | 2 | 8196020 |  | 13.19 |  | 2.24 | *GW2* | 74.369 | Song et al. 2007 |
|  | Both | 6 | 2 | 25079124~26049877 | 17.19 | 17.15~18.25 |  | 1.27~2.43 | *OsVPE3* | 148.959~811.321 | Lu et al. 2016 |
|  | RR | 7 | 2 | 28749717 | 15.39 |  |  | 1.74 | *GS2* | 113.557 | Hu et al. 2015 |
|  | MC | 8 | 2 | 30953174 | 6.37 |  |  | 2.51 | *OsNF-YB1* | 760.686 | Xu et al. 2016 |
|  | Both | 9 | 2 | 31404034~31234763 | 10.92~12.69 |  |  | 3.11~3.83 | *KRP1* | 707.277~876.548 | Barrôco et al. 2006 |
|  | MC | 10 | 2 | 33674994 |  |  | 5.14 | 0.32 | *SMG1* | 231.046 | Duan et al. 2014 |
|  | MC | 11 | 2 | 35005427 |  |  | 10.91 | 0.78 | *BSG1* | 315.704 | Yan et al. 2013 |
|  | RR | 12 | 3 | 6596936 |  | 23.59 |  | 4.26 | *LPA1* | 640.226 | Sun et al. 2019 |
|  | RR | 13 | 3 | 30587749 |  | 6.78 |  | 2.17 | *OsPho1* | 744.284 | Satoh et al. 2008 |
|  | MC | 14 | 3 | 35437797 |  |  | 11.11 | 0.43 | *qTGW3* | 45.815 | Ying et al. 2018 |
|  | RR | 15 | 4 | 4570606 | 24.24 |  |  | 4.63 | *ETR2* | 167.769 | Wuriyanghan et al. 2009 |
|  | MC | 16 | 4 | 10654189 |  | 14.47 |  | 1.52 | *OscZOG1* | 721.294 | Shang et al. 2016 |
|  | RR | 17 | 4 | 22027451 |  | 6.713 |  | 3.12 | *OsACOT* | 328.841 | Zhao et al. 2019 |
|  | RR | 18 | 4 | 24390487 |  |  | 21.48 | 1.66 | *D11* | 918.895 | Zhu et al. 2015 |
|  | RR | 19 | 4 | 27688228 |  |  | 9.08 | 2.14 | *OsMKKK10* | 361.018 | Xu et al. 2018 |
|  | Both | 20 | 4 | 31490102~31939665 | 26.49~29.73 | 12.46 | 46.01 | 1.25~4.60 | *FC1* | 416.375~865.938 | Li et al. 2009 |
|  | MC | 21 | 4 | 33256631~33294138 |  | 22.65 | 24.74 | 1.21~4.08 | *flo2* | 408.346~445.853 | She et al. 2010 |
|  | MC | 22 | 5 | 630924~1479400 | 10.48 |  | 11.09 | 0.62~3.78 | *GSN1* | 227.475~616.661 | Guo et al. 2018 |
|  | Both | 23 | 5 | 5356835~5895833 | 6.32 |  | 31.77 | 2.38~3.19 | *GW5* | 8.287~529.132 | Liu et al. 2017 |
|  | RR | 24 | 5 | 14579088 |  |  | 32.4 | 3.05 | *OsAGSW1* | 461.709 | Li et al. 2015 |
|  | MC | 25 | 6 | 1540336 | 4.83 |  |  | 1.91 | *SSG6* | 89.442 | Matsushima et al. 2016 |
|  | MC | 26 | 6 | 5384404 |  |  | 11.09 | 0.96 | *OsKASI* | 483.84 | Ding et al. 2015 |
|  | MC | 27 | 6 | 8254727 |  |  | 18.22 | 0.48 | *PFPβ* | 600.42 | Duan et al. 2016 |
|  | MC | 28 | 7 | 7640833~8364433 |  | 16.51 | 50.71 | 1.19~2.47 | *SSH1* | 90.919~814.519 | Jiang et al. 2019a |
|  | RR | 29 | 8 | 6110721 | 6.11 |  |  | 2.25 | *UAP1* | 126.728 | Wang et al. 2015b |
|  | MC | 30 | 8 | 25154283 | 13.02 |  |  | 3.19 | *OsSPL14* | 120.258 | Jiao et al. 2010 |
|  | MC | 31 | 8 | 27495394 |  |  | 16.65QE | 1.52 | *OsSPL16* | 989.196 | Wang et al. 2012 |
|  | Both | 32 | 10 | 463104~1426946 | 16~21.09 |  |  | 2.15~3.4 | *OsPCR1* | 361.731~600.637 | Song et al. 2015a |
|  | RR | 33 | 11 | 10422612 | 12.59 |  |  | 1.13 | *OsMPK15* | 943.258 | Hong et al. 2019 |

**References**

1. Barrôco, R. M., Peres, A., Droual, A. M., De Veylder, L., Nguyen, l., De Wolf, J., et al. (2006). The cyclin-dependent kinase inhibitor Orysa; *KRP1* plays an important role in seed development of rice. *Plant Physiol.* 142, 1053–1064. doi: 10.1104/pp.106.087056
2. Ding, W., Lin, L., Zhang, B., Xiang, X., Wu, J., Pan, Z., et al. (2015). *OsKASI*, a β-ketoacyl-[acyl carrier protein] synthase I, is involved in root development in rice (*Oryza sativa* L*.*). *Planta* 242, 203–213. doi: 10.1007/s00425-015-2296-2
3. Duan, E., Wang, Y., Liu, L., Zhu, J., Zhong, M., Zhang, H., et al. (2016). *Pyrophosphate: fructose-6-phosphate 1-phosphotransferase (PFP)* regulates carbon metabolism during grain filling in rice. *Plant Cell Rep.* 35, 1321–1331. doi: 10.1007/s00299-016-1964-4
4. Duan, P., Rao, Y., Zeng, D., Yang, Y., Xu, R., Zhang, B., et al. (2014). *SMALL GRAIN 1*, which encodes a mitogen-activated protein kinase kinase 4, influences grain size in rice. *Plant J. Cell Mol. Biol.* 77, 547–557. doi: 10.1111/tpj.12405
5. Fang, N., Xu, R., Huang, L., Zhang, B., Duan, P., Li, N., et al. (2016). *SMALL GRAIN 11* controls grain size, grain number and grain yield in rice. *Rice* 9, 64. doi: 10.1186/s12284-016-0136-z
6. Guo, T., Chen, K., Dong, N. Q., Shi, C. L., Ye, W. W., Gao, J. P., et al. (2018). *GRAIN SIZE AND NUMBER1* negatively regulates the OsMKKK10-OsMKK4-OsMPK6 cascade to coordinate the trade-off between grain number per panicle and grain size in rice. *Plant cell*. 30, 871–888. doi: 10.1105/tpc.17.00959
7. Ho, S. L., Huang, L. F., Lu, C. A., He, S. L., Wang, C. C., Yu, S. P., et al. (2013). Sugar starvation- and GA-inducible calcium-dependent protein kinase 1 feedback regulates GA biosynthesis and activates a 14-3-3 protein to confer drought tolerance in rice seedlings. *Plant Mol. Biol.* *81*, 347–361. doi: 10.1007/s1110-012-0006-z
8. Hong, Y., Liu, Q., Cao, Y., Zhang, Y., Chen, D., Lou, X., et al. (2019). The *OsMPK15* negatively regulates magnaporthe oryza and xoo disease resistance via SA and JA signaling pathway in rice. *Front. Plant Sci.* 10, 752. doi: 10.3389/fpls.2019.00752
9. Hu, J., Wang, Y., Fang, Y., Zeng, L., Xu, J., Yu, H., et al. (2015). A rare allele of *GS2* enhances grain size and grain yield in rice. *Mol. Plant* 8, 1455–1465. [doi: 10.1016/j.molp.2015.07.002](https://doi.org/10.1016/j.molp.2015.07.002)
10. Jiang, L., Ma, X., Zhao, S., Tang, Y., Liu, F., Gu, P., et al. (2019). The APETALA2-like transcription factor *SUPERNUMERARY BRACT* controls rice seed shattering and seed size. *Plant Cell*, 31, 17–36. doi: 10.1105/tpc.18.00304
11. Jiao, Y., Wang, Y., Xue, D., Wang, J., Yan, M., Liu, G., et al. (2010). Regulation of *OsSPL14* by OsmiR156 defines ideal plant architecture in rice. *Nat. Genet.* 42, 541–544. doi: 10.1038/ng.591
12. Li, X., Yang, Y., Yao, J., Chen, G., Li, X., Zhang, Q., et al. (2009). *FLEXIBLE CULM 1* encoding a cinnamyl-alcohol dehydrogenase controls culm mechanical strength in rice. *Plant Mol. Biol.*, 69(6), 685–697.
13. Li, Z., Tang, L., Qiu, J., Zhang, W., Wang, Y., Tong, X., et al (2016). Serine carboxypeptidase 46 regulates grain filling and seed germination in rice (*Oryza sativa* L.). *PloS one* 11, e0159737. doi: 10.1371/journal.pone.0159737
14. Liu, J., Chen, J., Zheng, X., Wu, F., Lin, Q., Heng, Y., et al. (2017). *GW5* acts in the brassinosteroid signalling pathway to regulate grain width and weight in rice. *Nat. Plants* 3, 17043. doi: 10.1038/nplants.2017.43
15. Liu, L., Tong, H., Xiao, Y., Che, R., Xu, F., Hu, B., et al. (2015). Activation of *Big Grain1* significantly improves grain size by regulating auxin transport in rice. *Proc. Natl. Acad. Sci.* *U. S. A.* 112, 11102–11107. doi: 10.1073/pnas.1512748112
16. Lu, W., Deng, M., Guo, F., Wang, M., Zeng, Z., Han, N., et al. (2016). Suppression of *OsVPE3* enhances salt tolerance by attenuating vacuole rupture during programmed cell death and affects stomata development in rice. *Rice* 9, 65. doi: 10.1186/s12284-016-0138-x
17. Mao, H., Sun, S., Yao, J., Wang, C., Yu, S., Xu, C., et al. (2010). Linking differential domain functions of the *GS3* protein to natural variation of grain size in rice. *Proc. Natl. Acad. Sci. U. S. A.* 107, 19579–19584. doi: 10.1073/pnas.1014419107
18. Matsushima, R., Maekawa, M., Kusano, M., Tomita, K., Kondo, H., Nishimura, H., et al. (2016). Amyloplast membrane protein *SUBSTANDARD STARCH GRAIN6* controls starch grain size in rice endosperm. *Plant Physiol.* 170, 1445–1459. doi: 10.1104/pp.15.01811
19. Nakagawa, H., Tanaka, A., Tanabata, T., Ohtake, M., Fujioka, S., Nakamura, H., et al. (2012). *Short grain1* decreases organ elongation and brassinosteroid response in rice. *Plant Physiol.* 158, 1208–1219. doi: 10.1104/pp.111.187567
20. Nayar, S., Sharma, R., Tyagi, A. K., and Kapoor, S. (2013). Functional delineation of rice *MADS29* reveals its role in embryo and endosperm development by affecting hormone homeostasis. *J. Exp. Bot.* 64, 4239–4253. doi: 10.1093/jxb/ert231
21. Qi, P., Lin, Y. S., Song, X. J., Shen, J. B., Huang, W., Shan, J. X., et al. (2012). The novel quantitative trait locus *GL3.1* controls rice grain size and yield by regulating *Cyclin-T1;3*. *Cell Res.* 22, 1666–1680. doi: 10.1038/cr.2012.151
22. Satoh, H., Shibahara, K., Tokunaga, T., Nishi, A., Tasaki, M., Hwang, S. K., et al. (2008). Mutation of the plastidial alpha-glucan phosphorylase gene in rice affects the synthesis and structure of starch in the endosperm. *Plant Cell* 20, 1833–1849. doi: 10.1105/tpc.107.054007
23. Shang, X. L., Xie, R. R., Tian, H., Wang, Q. L., and Guo, F. Q. (2016). Putative zeatin O-glucosyltransferase *OscZOG1* regulates root and shoot development and formation of agronomic traits in rice. *J. Integr. Plant Biol.* 58, 627–641. doi: 10.1111/jipb.12444
24. She, K. C., Kusano, H., Koizumi, K., Yamakawa, H., Hakata, M., Imamura, T., et al. (2010). A novel factor *FLOURY ENDOSPERM2* is involved in regulation of rice grain size and starch quality. *Plant Cell* 22, 3280–3294. doi: 10.1105/tpc.109.070821
25. Song, W. Y., Lee, H. S., Jin, S. R., Ko, D., Martinoia, E., Lee, Y., et al. (2015a). Rice *PCR1* influences grain weight and Zn accumulation in grains. *Plant cell Environ.* 38, 2327–2339. doi: 10.1111/pce.12553
26. Song, X. J., Huang, W., Shi, M., Zhu, M. Z., and Lin, H. X. (2007). A QTL for rice grain width and weight encodes a previously unknown RING-type E3 ubiquitin ligase. *Nat. Genet.* 39, 623–630. doi: 10.1038/ng2014
27. Song, X. J., Kuroha, T., Ayano, M., Furuta, T., Nagai, K., Komeda, N., et al. (2015b). Rare allele of a previously unidentified histone H4 acetyltransferase enhances grain weight, yield, and plant biomass in rice. *Proc. Natl. Acad. Sci. U. S. A.* 112, 76–81. doi: 10.1073/pnas.1421127112
28. Sun, Q., Li, T. Y., Li, D. D., Wang, Z. Y., Li, S., Li, D. P., et al. (2019). Overexpression of *Loose Plant Architecture 1* increases planting density and resistance to sheath blight disease via activation of PIN-FORMED 1a in rice. *Plant Biotechnol. J.* 17, 855–857. doi: 10.1111/pbi.13072
29. Tian, L., Dai, L. L., Yin, Z. J., Fukuda, M., Kumamaru, T., Dong, X. B., et al. (2013). *Small GTPase Sar1* is crucial for proglutelin and α-globulin export from the endoplasmic reticulum in rice endosperm. *J. Exp. Bot.*64, 2831–2845. doi: 10.1093/jxb/ert128
30. Wang, S., Lei, C., Wang, J., Ma, J., Tang, S., Wang, C., et al. (2017). *SPL33*, encoding an eEF1A-like protein, negatively regulates cell death and defense responses in rice. *J. Exp. Bot.* 68, 899–913. doi: 10.1093/jxb/erx001
31. Wang, S., Li, S., Liu, Q., Wu, K., Zhang, J., Wang, S., et al. (2015a). The *OsSPL16-GW7* regulatory module determines grain shape and simultaneously improves rice yield and grain quality. *Nat. Genet.* 47, 949–954. doi: 10.1038/ng.3352
32. Wang, S., Wu, K., Yuan, Q., Liu, X., Liu, Z., Lin, X., et al. (2012). Control of grain size, shape and quality by *OsSPL16* in rice. *Nat. Genet*. 44, 950–954. [doi: 10.1038/ng.2327](https://doi.org/10.1038/ng.2327)
33. Wang, Z., Wang, Y., Hong, X., Hu, D., Liu, C., Yang, J., et al. (2015b). Functional inactivation of *UDP-N-acetylglucosamine pyrophosphorylase 1 (UAP1)* induces early leaf senescence and defence responses in rice. *J. Exp. Bot.* 66, 973–987. doi: 10.1093/jxb/eru456
34. Wu, S., Xie, Y., Zhang, J., Ren, Y., Zhang, X., Wang, J., et al. (2015). *VLN2* regulates plant architecture by affecting microfilament dynamics and polar auxin transport in rice. *Plant Cell* 27, 2829–2845. doi: 10.1105/tpc.15.00581
35. Wuriyanghan, H., Zhang, B., Cao, W. H., Ma, B., Lei, G., Liu, Y. F., et al. (2009). The ethylene receptor *ETR2* delays floral transition and affects starch accumulation in rice. *Plant Cell* 21, 1473-1494. doi: 10.1105/tpc.108.065391
36. Xu, J. J., Zhang, X. F., and Xue, H. W. (2016). Rice aleurone layer specific *OsNF-YB1* regulates grain filling and endosperm development by interacting with an ERF transcription factor. *J. Exp. Bot.* 67, 6399–6411. doi: 10.1093/jxb/erw409
37. Xu, R., Duan, P., Yu, H., Zhou, Z., Zhang, B., Wang, R., et al. (2018). Control of grain size and weight by the OsMKKK10-OsMKK4-OsMAPK6 signaling pathway in rice. *Mol. Plant* 11, 860-873. doi: 10.1016/j.molp.2018.04.004
38. Yan, D., Zhou, Y., Ye, S., Zeng, L., Zhang, X., and He, Z. (2013). *Beak-shaped grain 1/TRIANGULAR HULL 1*, a DUF640 gene, is associated with grain shape, size and weight in rice. *Sci. China Life Sci.* 56, 275–283. doi: 10.1007/s11427-013-4449-5
39. Ying, J. Z., Ma, M., Bai, C., Huang, X. H., Liu, J. L., Fan, Y. Y., et al. (2018). *TGW3*, a major QTL that negatively modulates grain length and weight in rice. *Mol. Plant* *11*, 750–753. doi: 10.1016/j.molp.2018.03.007
40. Zhang, X., Wang, J., Huang, J., Lan, H., Wang, C., Yin, C., et al. (2012). Rare allele of *OsPPKL1* associated with grain length causes extra-large grain and a significant yield increase in rice. *Proc. Natl. Acad. Sci. U. S. A.* *109*, 21534–21539. doi: 10.1073/pnas.1219776110
41. Zhao, Y. F., Peng, T., Sun, H. Z., Teotia, S., Wen, H. L., Du, Y. X., et al. (2019). miR1432-*OsACOT* (Acyl-CoA thioesterase) module determines grain yield via enhancing grain filling rate in rice. *Plant Biotechnol. J.* 17, 712–723. doi: 10.1111/pbi.13009
42. Zhu, X., Liang, W., Cui, X., Chen, M., Yin, C., Luo, Z., et al. (2015). Brassinosteroids promote development of rice pollen grains and seeds by triggering expression of carbon starved anther, a MYB domain protein. *Plant J. Cell Mol. Biol.* 82, 570–581. doi: 10.1111/tpj.12820
